# A method combining a random forest-based technique with the modeling of linkage disequilibrium through latent variables, to run multilocus genome-wide association studies

**DOI:** 10.1186/s12859-018-2054-0

**Published:** 2018-03-27

**Authors:** Christine Sinoquet

**Affiliations:** LS2N, UMR CNRS 6004, Université de Nantes, 2 rue de la Houssinière, BP 92208, Nantes Cedex, 44322 France

**Keywords:** Genome-wide association study, GWAS, Multilocus approach, Random forest-based approach, Linkage disequilibrium modeling, Forest of latent tree models, Bayesian network with latent variables, Hybrid approach, Integration of biological knowledge to GWAS

## Abstract

**Background:**

Genome-wide association studies (GWASs) have been widely used to discover the genetic basis of complex phenotypes. However, standard single-SNP GWASs suffer from lack of power. In particular, they do not directly account for linkage disequilibrium, that is the dependences between SNPs (Single Nucleotide Polymorphisms).

**Results:**

We present the comparative study of two multilocus GWAS strategies, in the random forest-based framework. The first method, T-Trees, was designed by Botta and collaborators (Botta et al., PLoS ONE 9(4):e93379, 2014). We designed the other method, which is an innovative hybrid method combining T-Trees with the modeling of linkage disequilibrium. Linkage disequilibrium is modeled through a collection of tree-shaped Bayesian networks with latent variables, following our former works (Mourad et al., BMC Bioinformatics 12(1):16, 2011). We compared the two methods, both on simulated and real data. For dominant and additive genetic models, in either of the conditions simulated, the hybrid approach always slightly performs better than T-Trees. We assessed predictive powers through the standard ROC technique on 14 real datasets. For 10 of the 14 datasets analyzed, the already high predicted power observed for T-Trees (0.910-0.946) can still be increased by up to 0.030. We also assessed whether the distributions of SNPs’ scores obtained from T-Trees and the hybrid approach differed. Finally, we thoroughly analyzed the intersections of top 100 SNPs output by any two or the three methods amongst T-Trees, the hybrid approach, and the single-SNP method.

**Conclusions:**

The sophistication of T-Trees through finer linkage disequilibrium modeling is shown beneficial. The distributions of SNPs’ scores generated by T-Trees and the hybrid approach are shown statistically different, which suggests complementary of the methods. In particular, for 12 of the 14 real datasets, the distribution tail of highest SNPs’ scores shows larger values for the hybrid approach. Thus are pinpointed more interesting SNPs than by T-Trees, to be provided as a short list of prioritized SNPs, for a further analysis by biologists. Finally, among the 211 top 100 SNPs jointly detected by the single-SNP method, T-Trees and the hybrid approach over the 14 datasets, we identified 72 and 38 SNPs respectively present in the top25s and top10s for each method.

**Electronic supplementary material:**

The online version of this article (10.1186/s12859-018-2054-0) contains supplementary material, which is available to authorized users.

## Background

The etiology of genetic diseases may be elucidated by localizing genes conferring disease susceptibility and by subsequent biological characterization of these genes. Searching the genome for small DNA variations that occur more frequently in subjects with a peculiar disease (cases) than in unaffected individuals is the key to association studies. These DNA variations are observed at characterized locations - or loci - of the genome, also called genetic markers. Nowadays, genotyping technologies allow the description of case and control cohorts (a few thousand to ten thousand individuals) on the genome scale (hundred thousands to a few million of genetic markers such as Single Nucleotide Polymorphisms (SNPs)). The search for associations (i.e. statistical dependences) between one or several of the markers and the disease is called an association study. Genome-wide association studies (GWASs) are also expected to help identify DNA variations that affect a subject’s response to drugs or influence interactions between genotype and environment in a way that may contribute to the on-set of a given disease. Thus, improvement in the prediction of diseases, patient care and achievement of personalized medicine are three major aims of GWASs applied to biomedical research.

Exploiting the existence of statistical dependences between neighbor SNPs is the key to association studies [1, 2]. Statistical dependences within genetical data define linkage disequilibrium (LD). To perform GWASs, geneticists rely on a set of genetic markers, say SNPs, that cover the whole genome and are observed for any genotyped individual of a studied population. However, it is highly unlikely that a causal variant (i.e. a genetic factor) coincides with a SNP. Nevertheless, due to LD, a statistical dependence is expected between any SNP that flanks the unobserved genetic factor and the latter. On the other hand, by definition, a statistical dependence exists between the genetic factor responsible for the disease and this disease. Thus, a statistical dependence is also expected between the flanking SNP and the studied disease.

A standard single-SNP GWAS considers each SNP on its own and tests it for association with the disease. GWASs considering binary affected/unaffected phenotypes rely on standard contingency table tests (chi-square test, likelihood ratio test, Fisher’s exact test). Linear regression is broadly used for quantitative phenotypes.

The lack of statistical power is one of the limitations of single-SNP GWASs. Thus, multilocus strategies were designed to enhance the identification of a region on the genome where a genetical factor might be present. In the scope of this article, a “multilocus” strategy has to be distinguished from strategies aiming at epistasis detection. Epistatic interactions exist within a given set of SNPs when a dependence is observed between this combination of SNPs and the studied phenotype, whereas no marginal dependence may be evidenced between the phenotype and any SNP within this combination. Underlying epistasis is the concept of biological interactions between loci acting in concert as an organic group. In this article, a multilocus GWAS approach aims at focusing on interesting regions of the genome, through a more thorough exploitation of LD as in single SNP-GWASs.

**Fig. 1:**
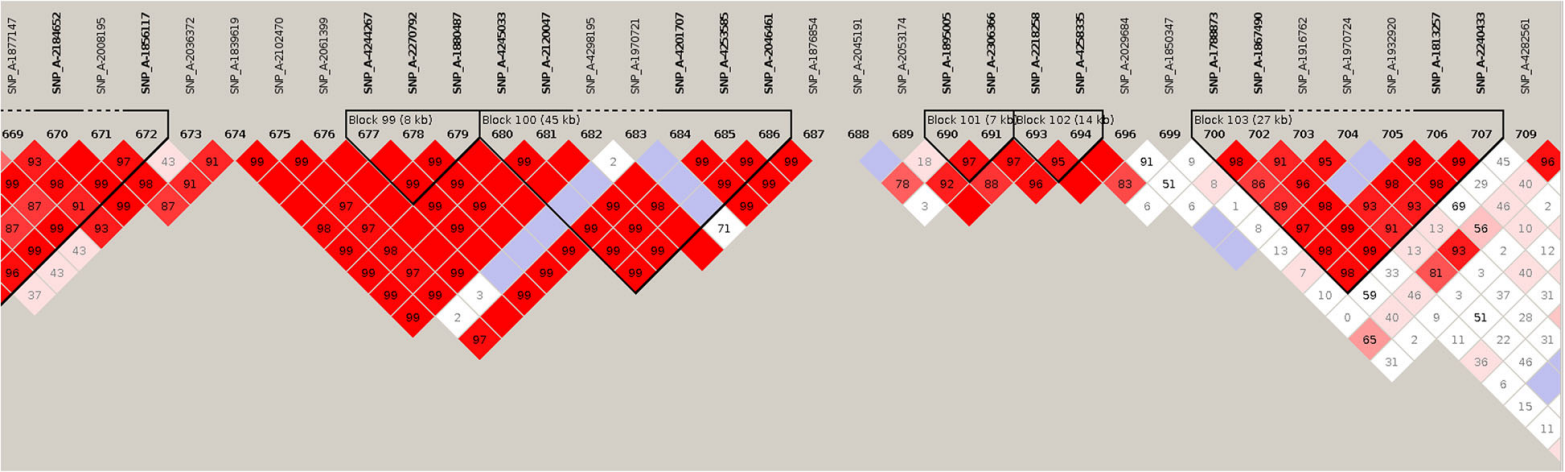
Illustration of linkage disequilibrium. Human chromosome 22. The focus is set on a region of 41 SNPs. Various color shades indicate the strengths of the correlation between the pairs of SNPs. The darkest (red) shade points out the strongest correlations. The white color indicates the smallest correlations. Blocks of pairwise highly correlated SNPs are highlighted in black. For instance, the block on the far right encompasses 7 SNPs in linkage disequilibrium

When inheriting genetic material from its parents, an individual is likely to receive entire short segments identical to its parents’ - called haplotypes -. Thus, as a manifestation of linkage disequilibrium - namely dependences of loci along the genome -, in a short chromosome segment, only a few distinct haplotypes may be observed over an entire population (see Fig. 1). Chromosomes are mosaics where extent and conservation of mosaic pieces mostly depend on recombination and mutation rates, as well as natural selection. Thus, the human genome is highly structured into the so-called “haplotype block structure” [3].

The most basic approach in the field of multilocus strategies, haplotype testing, relies on contingency tables to study haplotype distributions in the case and cohort groups. The traditional haplotype-based tests used in case-control studies are goodness-of-fit tests to detect a contrast between the case and control haplotype distributions [4]. Theoretical studies have shown that multi-allelic haplotype-based approaches can provide superior power to discriminate between cases and controls, compared to single-SNP GWASs, in mapping disease loci [5]. Besides, the use of haplotypes in disease association studies achieves data dimension reduction as it decreases the number of tests to be carried out.

However, one limitation is that haplotype testing requires the inference of haplotypes - or phasing -, a challenging computational task at genome scale [6, 7]. Another limitation is that when there are many haplotypes, there are many degrees of freedom and thus the power to detect association can be weak. Besides, the estimates for the rare haplotypes can be prone to errors as the null distribution may not follow a chi-square distribution. To cope with these issues, some works have considered haplotype similarity to group haplotypes into clusters. Thus, using a small number of haplotype clusters reduces the number of degrees of freedom and alleviates the inconvenience related to rare haplotypes. In this line, a variable length Markov chain model was designed by Browning and Browning to infer localized haplotype clustering and subsequently carry out an haplotype-based GWAS [8].

To accelerate haplotype-based GWASs, some authors rely on phase known references [9]. Allele prediction is achieved using a reference population with available haplotype information. To boost haplotype inference, Wan and co-authors only estimate haplotypes in relevant regions [10]. For this purpose, a sliding-window strategy partitions the whole genome into overlapping short windows. The relevance of each such window is analyzed through a two-locus haplotype-based test. Hardware accelerators are also used in the works reported in [11], to speed up the broadly used PHASE haplotype inference method [12].

The formidable challenge of GWASs demands algorithms that are able to cope with the size and complexity of genetical data. Machine learning approaches have been shown to be promising complements to standard single-SNP and multilocus GWASs [13, 14]. Machine learning techniques applied to GWASs encompass but are not limited to penalized regression (e.g. LASSO [15], ridge regression [16]), support vector machines [17], ensemble methods (e.g. random forests), artificial neural networks [18] and Bayesian network-based analyses [19, 20]. In particular, random forest-based methods were shown very attractive in the context of genetical association studies [21]. Random forest classification models can provide information on importance of variables for classification, in our case for classification between affected and unaffected subjects.

In this paper, we compare a variant of the random forest technique specifically designed for GWASs, T-Trees, and a novel approach combining T-Trees with the modeling of linkage disequilibrium through latent variables. The modeling relies on a probalistic graphical framework, using the FLTM (Forest of latent tree models) model. The purpose of the present work is to examine how the already high performances of T-Trees are affected when combining T-Trees with a more refined modeling of linkage disequilibrium than through blocks of contiguous SNPs as is done in T-Trees. In our innovative proposal, linkage disequilibrium is modeled into a collection of tree-shaped Bayesian networks each rooted in a latent variable. In this framework, these latent variables roughly play the role of haplotypes. In the remainder of this paper, we focus on binary phenotypes (i.e. affected/unaffected status).

The random forest technique settles the grounds of an ensemble method relying on the decision tree concept. In machine learning, a decision tree is a model used for classification purpose. However, building a decision tree often entails model overfitting, with detrimental consequences on the subsequent use of this model. Breiman thus introduced the random forest concept, to design an ensemble method to subsequently average prediction over a set of decision trees [22]: a random forest is thus a collection of decision trees built from variables that best determine between two classes. In the GWAS field, the two classes correspond to affected and unaffected statuses, and the variables involved in the trees are good candidate to explain the disease. Random forests have proven useful to analyze GWAS data [23].

However, the necessity to handle high-dimensional data has led to the proposal of variants. In [24], a two-stage procedure only allows pre-filtered SNPs as explanatory variables in the forest’s trees. Filtering separates informative and irrelevant SNPs in two groups, based on their *p*-values. In [25], the entire genome is randomly divided into subsets. A random forest is fit for each subset, to compute subranks for the SNPs. The definite ranks of the SNPs are defined based on these subranks and are then iteratively improved.

Among the GWAS strategies focused on random forests, the works of Botta and collaborators are specific in that they attempt to acknowledge linkage disequilibrium [26]. These works have resulted in the T-Trees model, an embedded model where the nodes in the trees of a random forest are themselves trees. From now on, we will refer to meta-trees having meta-nodes, together with embedded trees and nodes. Basic biological information is integrated in these internal trees, for which the variables (SNPs) to be chosen are selected from adjacent windows of same width covering the whole genome. However, a more refined multilocus approach can be designed, that drops the principle of windows, to better model linkage disequilibrium. Our proposal is to combine the T-Trees approach with another machine learning model, able to infer a map of SNP clusters. Such clusters of SNPs are meant to extend the notion of haplotype blocks to genotype clusters.

Many efforts have been devoted to model linkage disequilibrium. To achieve this aim at the genome scale, machine learning techniques involving probabilistic graphical models have been proposed in particular (see [27] and [28] for surveys). In this line, decomposable Markov random fields have been investigated through the works on interval graph sampling and junction tree sampling of Thomas and co-workers ([29] and [30], respectively), those of Verzilli and co-workers [20] and Edwards’ works [31]. Investigations focused on Bayesian networks with latent variables have resulted in two models: the hidden Markov model of Scheet and Stephens [12] underlying the PHASE method on the one hand, and the forest of latent tree models (FLTM) developed by Mourad and co-workers [32], on the other hand.

The aim of this methodological paper is to compare the original T-Trees method proposed by Botta and collaborators to the same method augmented with more refined biological knowledge. The blocks of SNPs are replaced with clusters of SNPs resulting from the modeling of linkage disequilibrium in the first layer of the FLTM model of Mourad and co-workers. This study is necessary to assess whether the T-Trees approach with LD integration provides similar or complementary results with respect to the original T-Trees strategy. In addition, these two multilocus strategies are compared to a standard single-SNP GWAS. The comparison is performed on fourteen real GWAS datasets made available by the WTCCC (Wellcome Trust Case Control Consortium) organization (https://www.wtccc.org.uk/).

## Methods

The first subsection provides a gentle introduction to the standard random forest framework. The objective is to pave the way for further explaining the workings of the more advanced T-Trees and hybrid FLTM / T-Trees methods. The second subsection presents T-Trees in a progressive way. It leads the reader through the two embedded levels (and according learning algorithms) of the T-Trees model. The FLTM model is presented in the third subsection, together with a sketch of its learning algorithm. The fourth subsection depicts the hybrid FLTM / T-Trees approach. Strong didactical concerns have motivated the unified presentation of all learning algorithms, to allow full understanding for both non-specialists and specialists. A final subsection focuses on the design of the comparative study reported in this paper.

### A random forest framework to run genome-wide association studies

Growing a decision tree is a supervized task involving a learning set. It is a recursive process where tree node creation is governed by cut-point identification. A cut-point is a pair involving one of the available variables, *v*^∗^, and a threshold value *θ*. Over all available variables, this cut-point best discriminates the observations of the current learning set with respect to the categories *c*_1_ and *c*_2_ of some binary categorical variable of interest *c* (the affected/unaffectetd status in GWASs). At the tree root, the first cut-point allows to split the initial learning set into two complementary subsets, respectively satisfying *v*^∗^≤*θ* and *v*^∗^>*θ*, for some identified pair ($\phantom {\dot {i}\!}v^{*}, \theta $). If the discrimination power of cut-point (*v*^∗^,*θ*) is high enough, one should encounter a majority of observations belonging to category *c*_1_ and category *c*_2_ (or symmetrically), for both subsets respectively. However, at some node, if all observations in the current learning set belong to the same category, the node needs no further splitting and recursion locally ends in this leaf. Otherwise, recursion will be continued, on both novel learning subsets resulting from splitting. Thus will be provided two subtrees, to be grafted to the current node under creation.







The generic scheme of the standard learning algorithm for decision trees is provided in Algorithm 1. Its ingredients are: a test to terminate recursion (line 1), recursion termination (line 2), and recursion preceded by cut-point identification (lines 4 to 7). We will rely on this reference scheme to highlight the differences with variants further considered in this paper. Recursion termination is common to this learning algorithm and the aforementioned variants. Algorithm 2 shows the instantiation of the former general scheme, in the case of standard decision tree growing. The conditions for recursion termination are briefly described in Algorithm 2 (see caption).

In the learning algorithm of a decision tree, exact optimization is performed (Algorithm 2, line 6 to 9): for each variable *v* in *V* and for each of the *i*_*v*_ values in its value domain $Dom(v) = \{ \theta _{v1}, \theta _{v2}, \cdots \theta _{v{i_{v}}} \}\phantom {\dot {i}\!}$, the discrimination power of cut-point (*v*, *θ*_*vi*_) is evaluated. If the cut-point splits the current learning set *D* into *D*_*ℓ*_ and *D*_*r*_, the quality of this candidate cut-point is commonly assessed based on the conditional entropy measure : *discriminatingScore*(*cut*−*point*, *D*, *c*)=*H*(*D*/*c*)−*w*_*ℓ*_×*H*(*D*_*ℓ*_/*c*)−*w*_*r*_×*H*(*D*_*r*_/*c*), where *H*(*X*/*Y*) denotes the conditional entropy ($H(X/Y) = \sum _{{x\in Dom(X),y\in Dom(Y)}}p(x,y)\log {\frac {p(x)}{p(x,y)}}$), *c* is the binary categorical variable, and *w*_*ℓ*_ and *w*_*r*_ denote relative sample set sizes. Thus, an optimal cut-point is provided for each variable *v* in *V*, through the maximization of *discriminatingScore* (Algorithm 2, line 7). Finally, the best optimal predicate over all variables in *V* is identified (Algorithm 2, line 9).

Single decision trees are subject to several limitations, and in particular a (very) high variance which makes them often suboptimal predictors in practice. A technique called bagging was proposed by Breiman to bring robustness in machine learning algorithms with regard to this aspect ([33]). Bagging conjugates bootstrapping and aggregating. The reader is reminded that bootstrapping is a resampling technique consisting in sampling with replacement from the original sample set. Bootstrapping allows to generate an ensemble of predictors learned from slightly different versions of the original learning set. Thus, in a prediction framework, robustness is brought through the aggregation of predictions across the predictors in the ensemble. Bagging was one of the motivations to design the ensemble technique yielding a random forest.

On the other hand, other searchers investigated the idea of building tree-based models through a stochastic tree-growing algorithm instead of a deterministic one, as in decision trees. The idea of combining bagging with randomization led to the random forest model [22]. In the random forest model consisting of *T* trees, two kinds of randomization are introduced [34, 35]: (i) global, through the generation of *T* bootstrap copies; (ii) local, at the node level, for which the computation of the optimal cut-point is no more performed exactly, namely over all variables in *V*, but instead over *K* variables selected at random in *V*. The second randomization source both aims at decreasing complexity for large datasets, and diminishing the variance.

Two of the three methods compared in the present study, T-Trees and the hybrid FLTM/T-Trees approach, are variants of random forests. For further reference, Algorithm 3 outlines the simple generic sketch that governs the growing of an ensemble of tree-based models, in the random forest context. It has to be noted that a novel set of *K* variables is sampled at each node, to compute the cut-point at this node. It follows that the instantiations of generic Algorithm 1 (**growTree**) into **growDecisionTree** (Algorithm 2), and **growRFTree** adapted to the random forest framework (Appendix, Algorithm 7), only differ in the cut-point identifications. Table 1(A) and 1(B) show the difference between **growDecisionTree** and **growRFTree**. For the report, the full learning procedure **growRFTree** is depicted in Algorithm 7 in Appendix.

**Table 1:** Differences between the implementations of cut-point identification at a current node, for various instantiations of **growTree**

	

(A) **growDecisionTree**. (B) **growRFTree**. (C) **growExtraTree**. Functions **growDecisionTree**, **growRFTree** and **growExtraTree** are the instantiations of the generic function **growTree** (Algorithm 1), in the standard decision tree learning context, the random forest learning context, and the Extremely randomized tree (Extra-tree) context, respectively. Functions **growDecisionTree** and **growRFTree** are respectively detailed in Algorithm 2 (main text) and Algorithm 7 (Appendix). Complexity decreases across the three compared functions from exact optimization on the whole set *V* of variables, through exact optimization restrained to a random subset *V*_*aleat*_ of *V*, and to optimization over the cut-points selected at random for the variables in a random subset *V*_*aleat*_

**Fig. 2:**
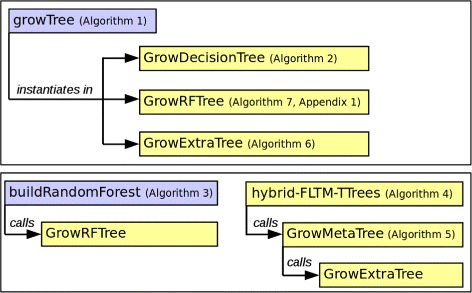
Synoptic view of the relationships between the algorithms introduced in the article. Rectangles filled with the darkest (blue) color shade indicate generic algorithms. Rectangles filled with the lightest (yellow) color shade indicate detailed instances of the algorithms

For a gradual introduction to the hybrid FLTM / T-Trees approach, we will refer to various algorithms in the remaining of the paper. The relationships between these algorithms are described in Fig. 2.

### The T-Trees approach

**Fig. 3:**
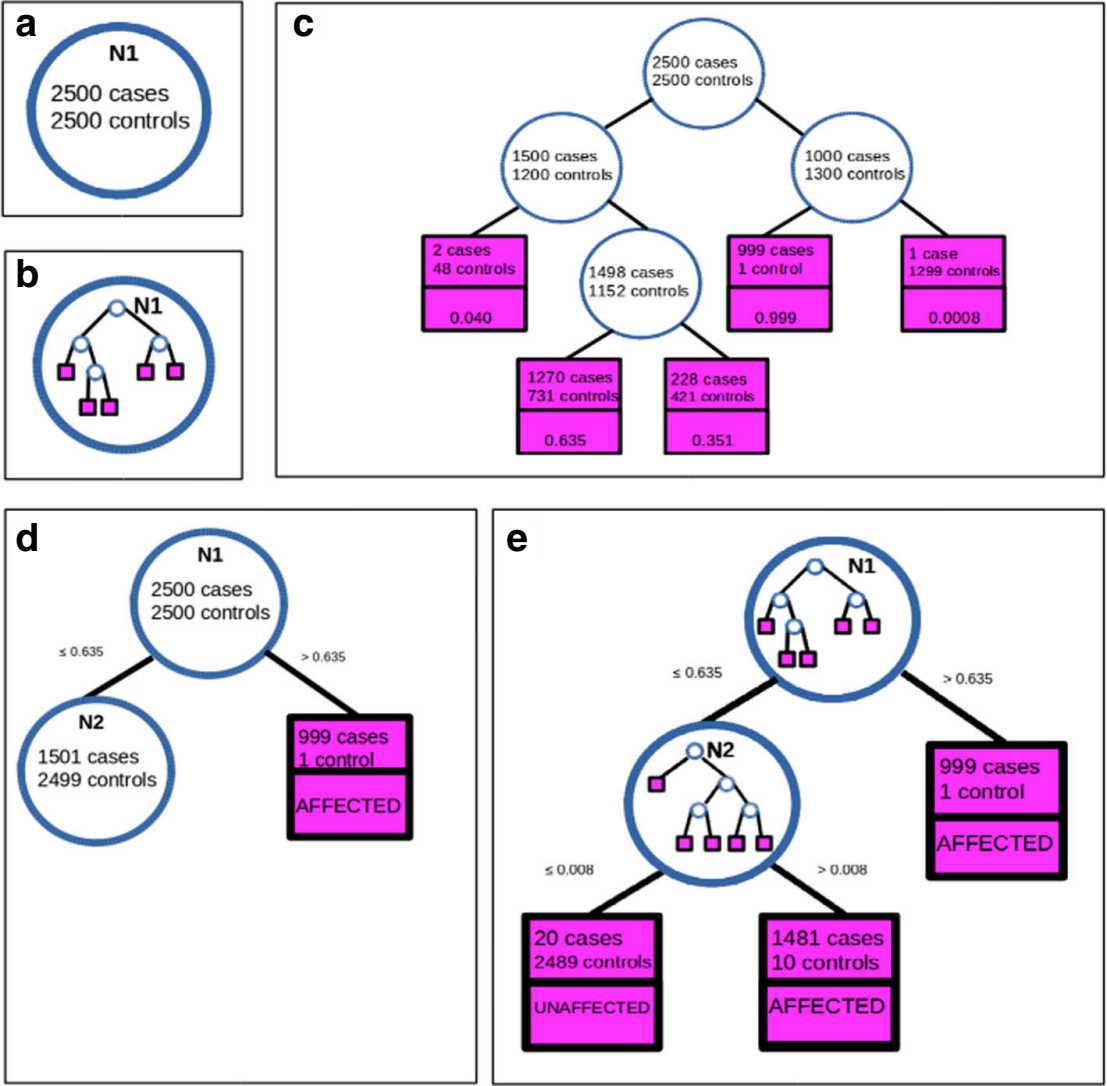
The embedded structure of the T-Trees model. The focus is set on the expansion of one meta-tree. **a** First meta-node N1. **b** Extra-tree embedded in meta-node N1. **c** Details of the Extra-tree embedded in meta-node N1. The value indicated in each leaf is the probability to be a case in this leaf. The five values 0.0008, 0.040, 0.351, 0.635 and 0.999 define the value domain of the meta-variable that corresponds to meta-node N1. **d** Threshold 0.635 is the best threshold among the five values of the meta-variable to discrimate between affected and unaffected subjects. Node N1 is splitted accordingly. As regards the left subtree expansion of N1, a novel meta-node N2 is created. Right subtree expansion of N1 ends in a meta-leaf (number of subjects below threshold 2000). **e** Whole meta-tree grown with its two embedded trees

The novelty in the T-Trees approach is that it treats more than one variable at each of the nodes, in the context of association studies [36]. In the GWAS context, the reason to modify the splitting process lies in the presence of dependences within the SNPs (i.e. within the variables in *V*), called linkage disequilibrium. This peculiar structure of the data entails an expectation of limited haplotype diversity, locally on the genome. Based on the physical order of the SNPs along the genome, the principle of T-Trees approach is to partition the set of variables *V* into blocks of *B* contiguous and (potentially highly) correlated variables. Each split will then be made on a block of SNPs instead of a single SNP, taking advantage of the local information potentially carried by the region covered by the corresponding block. However, addressing node splitting based on several variables was quite a challenge. For this purpose, Botta and collaborators customized a random forest model where each node in any tree embeds itself a tree. This “trees inside trees” model is abbreviated in T-Trees. Figure 3 describes the structure of a T-Trees model. Basically, the splitting process used in any node (or rather meta-node) of the random forest is now modified as follows: it involves a block of *B* variables, selected from *K* candidate blocks, instead of a single variable selected from *K* candidate variables as in random forests. In the case of GWASs, each block consists of *B* consecutive SNPs. For each meta-node, an embedded tree is then learned from a subset of *k* variables selected at random from the former block of *B* variables. Thus, it has to be noted that an additional source of randomization is brought to the overall learning algorithm: *k* plays in embedded tree learning the same role as the aforementioned parameter *K* plays in learning the trees at the random forest level. Only, to lower the complexity, *k* is much smaller than *K* (e.g. *K* is in the order of magnitude 10^3^, *k* is less than few tens). Above all, overall T-Trees learning tractability is achieved through the embedding of trees that are weak learners. Aggregating multiple weak learners is often the key to ensemble strategies’ efficiency and tractability [37]. The weak embedded learner used by Botta and co-workers is inspired from the one used in the ensemble Extremely randomized tree framework proposed by Geurts and co-workers [38]. Following these authors, the abbreviation for Extremely randomized tree is Extra-tree.

In the Extra-tree framework, a key to diminishing the variance is the combination of explicit randomization of cut-points with ensemble aggregation. Just as importantly, explicit randomization of cut-points also intends to diminish the learning complexity for the whole ensemble model, as compared to the standard random forest model. We now focus of the basic brick, the (single) Extra-tree model, when embedded in the T-Trees context. The Extra-tree model drops the idea of identifying an optimal cut-point for each of the *k* variables selected at random among the *B* variables in a block. Instead, this method generates the *k* candidate cut-points at random and then identifies the best one. Table 1(C) highlights the differences with the cut-point identifications in **growDecisionTree** and **growRFTree** (Table 1(A) and 1(B)). However, embedding trees presents a challenge for the identification of the cut-point at a meta-node (for each meta-node of the random forest, in the T-Trees context). So far, we know that, at a meta-node *n* with current learning set *D*_*n*_, the solution developed in the T-Trees framework selects at random *K* blocks $\mathcal {B}_{1} \cdots \mathcal {B}_{K}$ of *B* variables each, and accordingly learns *K* Extra-trees *ET*_1_⋯*ET*_*K*_. In turn, each Extra-tree *ET*_*b*_ is learned based on *k* variables selected from block $\mathcal {B}_{b}$. Now the challenge consists in being able to split the current learning set *D*_*n*_, based on some cut-point involving a *meta-variable* to be inferred. This novel numerical feature has to reflect the variables exhibited in Extra-tree *ET*_*b*_. Botta and co-workers define this novel numerical feature *ν* as follows: for Extra-tree *ET*_*b*_, the whole current learning set *D*_*n*_ (of observations) has been distributed into *ET*_*b*_’s leaves; each leaf is then labeled with the probability to belong to, say, category *c*_1_ (e.g. 0.3); therefore, for each observation *o* in *D*_*n*_ reaching leaf $\mathcal {L}$, this meta-variable is assigned $\mathcal {L}$’s label (e.g. *ν*(*o*)=0.3); consequently, the domain of the meta-variable can be defined (*Dom*(*ν*)={*ν*(*o*), *o*∈*observations*(*D*_*n*_)}); finally, it is straightforward to identify a threshold *θ*_*b*_ that optimally discriminates *D*_*n*_ over the domain value of the meta-variable. The previous process described to identify the threshold *θ*_*b*_ for a meta-variable plays the role of function **OptimalCutPoint** in the generic scheme of random forest learning (line 8 of Algorithm 7, Appendix). We wish to emphasize here that the vast performance assessment study of the T-Trees method conducted by Botta [36] evidenced high predictive powers (i.e. AUCs over 0.9 - The concept of AUC will be further recalled in Section Methods / Study design / Road map). Since the T-Trees method was empirically shown efficient, the explanation for such high performances lies in the core principles underlying T-Trees design: (i) transformation of the original input space into blocks of variables corresponding to contiguous SNPs potentially highly correlated, due to linkage disequilibrium and (ii) replacement of the classical univariate linear splitting process by a multivariate non-linear splitting scheme of several variables belonging to a same block.

### The FLTM approach

In contrast with the “ensemble method” meaning of “forest” in the two previous subsections, the Forest of Latent Tree Models (FLTM) we now focus on is a tree-shaped Bayesian network with discrete observed and latent variables.

A Bayesian network is a graphical model that encodes probabilistic relationships among *n* variables, each described for *p* observations. The nodes of the Bayesian network represent the variables, and the directed edges in the graph represent direct dependences between variables. A probability distribution over the *p* observations is associated to each node. If the node corresponding to variable *v* has parents *Pa*_*v*_, this distribution is conditional (*P*(*v*/*Pa*_*v*_)). Otherwise, this distribution is marginal (*P*(*v*)). The collection of probability distributions over all nodes is called the parameters.

**Fig. 4:**
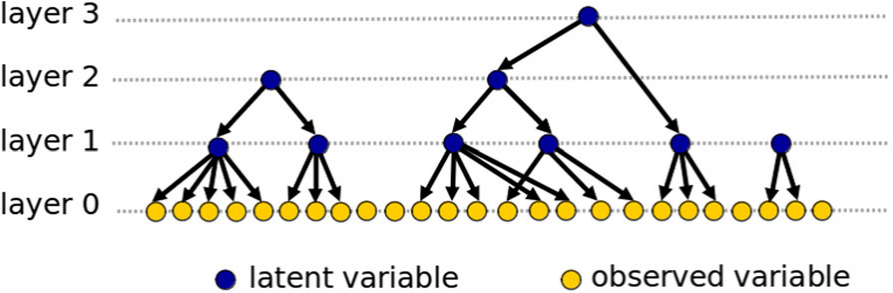
The forest of latent tree models (FLTM). This forest consists of three latent trees, of respective heights 2, 3 and 1. The observed variables are shown in light shade whereas the dark shade points out the latent variables

The FLTM model was designed by Mourad and collaborators for the purpose of modeling linkage disequilibrium (LD) at the genome scale. Indeed, the frontiers between regions of LD are fuzzy and a hierarchical model allows to account for such fuzziness. LD is learned from an *n*×*p* matrix (i.e. *n* SNPs ×*p* individuals). FLTM-based LD modeling consists in building a specific kind of Bayesian network with the *n* observed variables as tree leaves and latent variables as internal nodes in the trees. The structure of an FLTM model is depicted in Fig. 4.

**Fig. 5:**
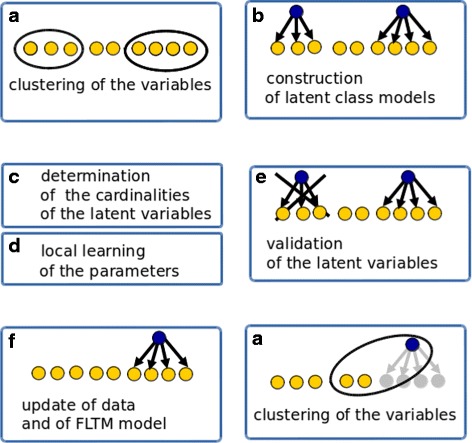
Principle of the learning algorithm of the FLTM model. Illustration for first iteration. **a** Given some scalable clustering method, the observed variables are clustered into disjoint clusters. **b** For each cluster *C* of size at least 2, a latent class model (LCM) is straightforwardly inferred. An LCM simply connects the variables in cluster *C* to a new single latent variable *L*. **c** The cardinality of this single latent variable is computed as an affine function of the number of child nodes in the LCM, controled with a maximum cardinality. **d** The EM algorithm is run on the LCM, and provides the LCM’s parameters (i.e. the probability distributions of the LCM’s nodes). **e** Now the probability distribution is known for *L*, the quality of the latent variable is assessed as follows: the average mutual information between *L* and any child in *C*, normalized by the maximum of entropies of *L* and any child in *C*, is compared to a user-specified threshold (*τ*); with mutual information defined as $MI(X,Y) = \sum _{x\in Dom(X)}\ \sum _{y\in Dom(Y)}\ \mathbb {P}(x,y)\log {\frac {\mathbb {P}(x,y)}{\mathbb {P}(x)\mathbb {P}(y)}}$, and entropy defined as $H(X) = - \sum _{x\in Dom(X)} \mathbb {P}(x)\log \mathbb {P}(x)$. **f** If the latent variable is validated, the FLTM model is updated: in the FLTM under construction, a novel node representing *L* is connected to the variables in *C*; the former probability distribution $\mathbb {P}(ch)$ of any child variable *ch* in *C* is replaced with $\mathbb {P}(ch / L)$. The probability distribution $\mathbb {P}(L)$ is stored. Finally, the variables in *C* are no more referred to in the data, latent variable L in considered instead. The updated graph and data are now ready for the next iteration. This process is iterated until all remaining variables are subsumed by one latent variable or no new valid latent variable can be created. For any latent variable *L*, and any observation *j*, data can be inferred through sampling based on probability distribution $\mathbb {P}(L / C)$ for *j*’s values of child variables in cluster *C*.

Learning a latent tree is challenging in the high dimensional case. There exist $O\left (2^{3n^{2}}\right)$ candidate structures for a latent tree derived from *n* observed variables [39]. Learning the tree structure can only be efficiently addressed through iterative ascending clustering of the variables [40]. A similarity measure based on mutual information is usually used to cluster discrete variables. On the other hand, parameter learning requires time-consuming procedures such as the Expectation-Maximization (EM) algorithm in the case of missing data. Dealing with latent variables represents a subcase of the missing data case. The FLTM learning algorithm is sketched and commented in Fig. 5.

To allow a faithful representation of linkage disequilibrium, a great flexibility of FLTM modeling was an objective of Mourad and collaborators’ works: (i) No fixed cluster size is required; (ii) The SNPs allowed in the same cluster are not necessarily contiguous on the genome, which allows long range disequilibrium modeling (iii) In the FLTM model, no two latent variables are constrained to share some user-specified cardinality. The reason of the FLTM learning algorithm tractability is four-fold: (i) Variables are allowed in the same cluster provided that there are located within a specified physical distance on the genome. Handling a sparse similarity matrix is affordable whereas using a pairwise matrix would not; (ii) Local learning of latent class model (LCM) has a complexity linear in the number of LCM’s child nodes; (iii) A heuristics in constant time provides the cardinality required by EM for the latent variable of each LCM; (iv) There are at most 3 *n* latent variables in a latent tree built from *n* observed variables.

### The hybrid FLTM / T-Trees approach

Now the ingredients to depict the hybrid approach developed in this paper are in place. In T-Trees, the blocks of *B* contiguous SNPs are a rough approximation of linkage disequilibrium. In contrast, each latent variable in layer 1 of the FLTM model pinpoints a region of LD. The connection between the FLTM and T-Trees models is achieved through LD mapping. The block map required by T-Trees in the original proposal is replaced with the cluster map associated with the latent variables in layer 1. It has to be emphasized that this map consisting of clusters of SNPs is not the output of a mere clustering process: in Fig. 5e, a latent variable and thus its corresponding cluster are validated following a procedure involving EM learning for Bayesian network parameters.

The hybrid approach is fully depicted and commented in Algorithms 4, 5 and 6. Hereinafter, we provide a broad brush description. In Algorithm 4, the generic random forest scheme of Algorithm 3 achieving global randomization is enriched with the generation of the LD map through FLTM modeling (lines 1 and 2). This map is one of the parameters of the function **growMetaTree** (Algorithm 4, line 6). The other parameters of **growMetaTree** will respectively contribute to shape the meta-trees in the random forest (*S*_*n*_,*S*_*t*_, *K*) and the embedded trees (*s*_*n*_,*s*_*t*_, *k*) associated to the meta-nodes. Both parameters *K* and *k* achieve local randomization. In addition, function **growMetaTree** differs from **growRFTree** (Appendix, Algorithm 7) in two points: it must expand an embedded tree through function **growExtraTree** (Algorithm 5, line 8) for each of *K* clusters drawn from the LD map; it must then infer data for the meta-variable defined by each of the *K* Extra-trees, to compute the optimal cut-point for each such meta-variable (optimalCutPointTTrees, Algorithm 5, line 9). Algorithm 6 fully details function **growExtraTree**, in which identification of cut-points achieves a further step of randomization (line 8).

In a random forest-based approach, the notion of variable importance used for decision trees is modified to include in *Nodes*(*v*) the set of all nodes, over all *T* trees, where *v* is used to split. As such, this measure is however dependent on the number *T* of trees in the forest. Normalization is used to divide the previous measure (over the *T* trees) by the sum of importances over all variables. Alternatively, dividing by the maximum importance over all variables may be used.

**Fig. 6:**
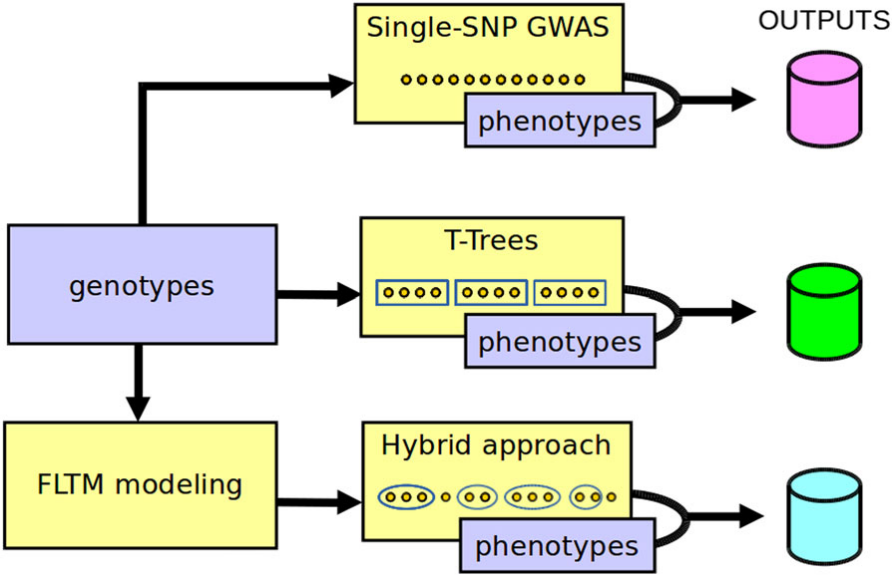
Outline of the study. In the single SNP GWAS, SNPs are tested one at a time for association with the disease. In the T-Trees method, the cut-point in any meta-node of the T-Trees random forest is computed based on blocks of, say, 20 contiguous SNPs. In the hybrid FLTM / T-Trees approach, FLTM modeling is used to provide a map of clusters; the cut-point in any meta-node of the hybrid random forest is calculated from clusters of SNPs output by the FLTM model



In the GWAS context, the differences between standard single-SNP GWAS, the T-Trees approach and the hybrid FLTM / T-Trees approach are schematized in Fig. 6.

### Study design

In this last subsection, we first present the data used in our comparative analysis. Then, details are provided regarding software implementation, including considerations about the validation of the software parallelization. We next describe the parameter setting for the methods involved in the comparative study. Finally, we provide the road map of our methodological analysis.

#### Simulated data

To simulate realistic genotypic data and an association between one of these SNPs and the disease status, we relied on one of the most widely-used software programs, HAPGEN (http://mathgen.stats.ox.ac.uk/genetics_software/hapgen/hapgen2.html) [41]. To control the effect size of the causal SNPs, three ingredients were combined: severity of the disease expressed as genotype relative risks (GRRs) for various genetic models (GMs), minor allele frequency (MAF) of the causal SNP. The genetic model was specified among additive, dominant or recessive. Three genotype relative risks were considered (1.2, 1.5 or 1.8). The range of the MAF at the causal SNP was specified within one of the three intervals [0.05-0.15], [0.15-0.25] or [0.25-0.35]. The disease prevalence (percentage of cases observed in a population) specified to HAPGEN was set to 0.01. These choices are justified as standards used for simulations in association genetics.

HAPGEN was run on a reference haplotype set of the HapMap phase II coming from U.S. residents of northern and western European ancestry (CEU). Datasets of 20000 SNPs were generated for 2000 cases and 2000 controls. Each condition (GM, GRR, MAF) was replicated 30 times. For each replicate, we simulated 10 causal SNPs. Standard quality control for genotypic data was carried out: we removed SNPs with MAF less than 0.05 and SNPs deviant from Hardy-Weinberg Equilibrium with a *p*-value below 0.001.



#### Real data

The GWAS data we used was made available by the WTCCC (Wellcome Trust Case Control Consortium) organization (https://www.wtccc.org.uk/). The WTCCC provides GWAS data for seven pathologies: bipolar disorder (BD), coronary artery disease (CAD), Crohn’s disease (CD), hypertension (HT), rheumatoid arthritis (RA), Type 1 diabetes (T1D) and Type 2 diabetes (T2D). The data from the two cohort controls provided by the WTCCC was also included. For each pathology, we carried out a comparative study on two datasets corresponding to two chromosomes.

The NHGRI-EBI Catalog of published genome-wide association studies (https://www.ebi.ac.uk/gwas/) allowed us to select these two chromosomes: for each pathology, we retained the chromosomes respectively showing the highest and lowest numbers of published associated SNPs so far. Table 2 recapitulates the description of the 14 WTCCC datasets selected. A quality control phase based on specifications provided by the WTCCC Consortium was performed [42]. In particular, SNPs were dismissed based on three rules: missing data percentage greater than 5%, missing data percentage greater than 1% together with frequency of minor allele (MAF) less than 5%; *p*-value for exact Hardy-Weinberg equilibrium test less than 5.7×10^−7^; *p*-value threshold for trend test (1 ddl) equal to 5.7×10^−7^ and *p*-value threshold for general test (2 ddl) equal to 5.7×10^−7^.

**Table 2:** Description of the 14 GWAS datasets selected

Pathology	Chromosome	Number	Number of	Number of associated
		of SNPs	individuals	SNPs published
BD	Chr03	31554	4806	37
	Chr21	6645		3
CAD	Chr05	29946	4864	11
	Chr06	28085		−
CD	Chr01	37267	4686	31
	Chr20	11586		1
HT	Chr10	26635	4890	8
	Chr14	14640		−
RA	Chr06	28085	4798	59
	Chr19	5845		2
T1D	Chr02	38730	4901	16
	Chr13	17999		1
T2D	Chr10	26635	4862	51
	Chr21	6645		3

The last column refers to the associated SNPs published in the NHGRI-EBI Catalog of published genome-wide association studies (https://www.ebi.ac.uk/gwas/). BD: bipolar disorder. CAD: coronary artery disease. CD: Crohn’s disease. HT: hypertension. RA: rheumatoid arthritis. T1D: Type 1 diabetes. T2D: Type 2 diabetes



#### Implementation

The T-Trees (sequential) software written in C++ was provided by Botta. A single run is highly time-consuming for GWASs in the orders of magnitude we have to deal with. For example, on a processor INTEL Xeon 3.3 GHz, running T-Trees on chromosome 1 for Crohn’s disease requires around 3 days. In these conditions, a 10-fold cross-validation (to be further described) would roughly require a month. On the other hand, around 5 GB are necessary to run T-Trees with the parameter values recommended by Botta [36], which restrains the number of executions in parallel. The only lever of action left was to speed up T-Trees software through parallelization. We parallelized Botta’s code using the OpenMP application programming interface for parallel programming (http://www.openmp.org/).

For the purpose of this study we also developed the third version of our FLTM learning algorithm [43]. It was written in C++ and relies on the ProBT library dedicated to Bayesian networks (http://www.probayes.com/fr/, http://www.probayes.com/fr/recherche/probt/, [44, 45]). In this version, the user is proposed a choice of three clustering methods. The one used in the present work is DBSCAN (Density-Based Spatial Clustering of Applications with Noise) [46]. In clustering methods belonging to the density-based category, a cluster is defined as a dense component able to grow in any direction density leads to. In this category, DBSCAN was chosen as it meets two essential criteria: non-specification of the number of clusters and ability to scale well. The theoretical runtime complexity of DBSCAN is *O*(*n*^2^), where *n* denotes the number of items to be grouped into clusters. Nonetheless, the empirical complexity is known to be lower. DBSCAN requires two parameters: *R*, the maximum radius of the neighborhood to be considered to grow a cluster, and *N*_*min*_, the minimum number of neighbors required within a cluster. Details about the DBSCAN algorithm are available in [47] (page 526, http://www-users.cs.umn.edu/~kumar/dmbook/ch8.pdf).

Finally, we wrote scripts (in Python) to automatize the comparison of the results provided by the three GWAS strategies: single-SNP GWAS, T-Trees, and hybrid FLTM/ T-Trees approach.

#### Validation of the software parallelization

Parallelization is known to potentially entail biases in results. We therefore took great care to check whether this was the case for the parallelization of T-Trees software. We recall that T-Trees is stochastic, with four sources of randomization. Thus, we controled randomization through fixing random grains and we compared the sequential and parallelized versions of T-Trees on five datasets. The comparison shows that importance measures are practically indentical, with some rare minor discrepancies (results not shown). In any case, SNP ranking is conserved from sequential to parallelized version.

#### Parameter setting

T-Trees and the hybrid FLTM / T-Trees approach share 7 parameters (T, *S*_*n*_,*S*_*t*_, *K*, *s*_*n*_,*s*_*t*_, *k*). We tuned them after the experiments and remarks reported in [36]. In T-Trees, the size of the blocks of SNPs was set to 20. The FLTM learning algorithm requires 6 parameters ($\phantom {\dot {i}\!}\alpha, \beta, card_{max}, \tau, nb-EM-restarts, \delta $). All 13 parameters are depicted in Table 3. The values of *α*,*β*,*τ* and $nb-EM-restarts\phantom {\dot {i}\!}$ were set after the indications in [32]. The value of *card*_*max*_ was set according to our own experimental feedback. The value for *δ* was chosen to control the running time in the case of high dimensional data. In addition, the clustering procedure DBSCAN plugged into FLTM learning requires 2 parameters (*R*, *N*_*min*_). To avoid questionable empirical setting of the *R* parameter, for each of the 14 datasets analyzed, we ran the FLTM learning algorithm for a wide range of possible values of *R*. For each dataset, we retained the FLTM model with the *R* parameter that optimized an entropy-based criterion. Table 3 recapitulates these 15 parameters and indicates the parameter setting or the parameter interval chosen for the present study.

**Table 3:** Parameter setting for the experimentations. The T-Trees parameters were tuned according to the experiments and remarks reported in [36]. In the hybrid FLTM / T-Trees approach, *k* was automatically adjusted to the current cluster size. The values of the FLTM parameters were set after indications from [32], except for *card*_*max*_ and *δ* which were tuned according to our own experience. The cardinality of a latent variable *L* is computed as an affine function of the number of child SNPs, *n*_*c*_: *card*(*L*)=*min*(*α*+*β*×*n*_*c*_, *card*_*max*_). To avoid questionable empirical setting of DBSCAN’s *R* parameter, for each of the 14 datasets analyzed, we ran the FLTM learning algorithm for a wide range of possible values of *R*. For each dataset, we retained the FLTM model with the *R* parameter that optimized an entropy-based criterion. The value of the other DBSCAN’s parameter, *N*_*min*_, was set to the minimum

Method	Parameter	Description	Value
T-Trees and hybrid approach		Size for the blocks of contiguous SNPs (T-Trees)	20
	*T*	Number of meta-trees in the random forest	1000
	*S*_*n*_	Threshold size (in number of observations), to control meta-tree leaf size	2000
	*S*_*t*_	Threshold size (in number of meta-nodes), to forbid expanding a meta-tree beyond this size	*∞*
	*K* (T-Trees)*K* (hybrid)	Number of contiguous blocks of SNPs, or number of clusters in LDMap, to be selected at random at each meta-node, to compute its cut-point	1000
	*s*_*n*_	Threshold size (in number of observations), to control embedded tree leaf size	1
	*s*_*t*_	Threshold size (in number of nodes), to forbid expanding an embedded tree beyond this size	5
	*k*	Number of variables in a block (T-Trees) or cluster (hybrid), to be selected at random, at each node, to compute its cut-point	size of block or of cluster
FLTM	*α*	Three parameters to model the cardinality of each	0.2
	*β*	latent variable as an affine function with a maximum	2
	*c* *a* *r* *d*_*max*_	threshold	10
	*τ*	Threshold to control the quality of latent variables	0.3
	*nb*−*EM*−*restarts*	Number of random restarts for the EM algorithm	10
	*δ*	Maximal physical distance (bp), to allow two SNPs in the same cluster	50×10^3^
DBSCAN	*R*	Maximum radius of the neighborhood to be considered to grow a cluster	value selected in 0.05 to 0.9, step 0.05
	*N*_*min*_	Minimum number of points required within a cluster	2

#### Road map

Our methodological analysis consisted of four main tasks.

#### Comparison of the performances of T-Trees and the hybrid approach on simulated data

We computed the percentage of the causal SNPs found among the top results, over all replicates related to the same condition (GM, GRR, MAF). We successively computed this percentage for the top 25, top 50, top 100, top 200 and top 1000 results.

#### Comparison of the predictive powers of T-Trees and the hybrid approach on real data

ROC (Receiver operator characteristic) curves help quantify and compare the powers of classifiers [48]. In the context of disease prediction, positives (P) and negatives (N) respectively stand for affected and unaffected. The ROC curve involves four proportions: TPs (true positives), FNs (false negatives), TNs (true negatives), and FPs (false positives). The ROC curve plots sensitivity (*TP*/*P*=*TP*/(*TP*+*FN*)) against 1 − specificity (*TN*/*N*=*TN*/(*TN*+*FP*)). Assuming positive statuses rank higher than negative statuses, the area under the curve (AUC) is equal to the probability that the prediction tool under analysis will rank a randomly chosen affected subject higher than a randomly chosen unaffected one. A high AUC is expected for a good classifier. We compared the AUCs obtained for T-Trees and the hybrid FLTM / T-Trees approach on 14 datasets, following a 10-fold cross validation scheme. In this scheme, the initial dataset *D* is split into 10 smaller datasets of equal size {*D*_*i*_, 1≤*i*≤10}. The model is trained on nine tenths of the data (training set) and tested on the remaining tenth (testing set). This process is iterated ten times. The *i*^*th*^ iteration involves testing set *D*_*i*_. It is important to note that in the hybrid FLTM / T-Trees approach, the LD map provided by FLTM is the same for all 10 iterations.

#### Comparison of the distributions of variable importances obtained from T-Trees and the hybrid approach on real data

A third task consisted in comparing the distributions of variable importances for the two random forest-based methods. Besides descriptive statistics, a peer analysis relying on Wilcoxon rank sum test and Pearson correlation was performed.

#### Analysis of the SNP sets jointly identified by any two among the three, or the three methods compared on real data

In contrast to single-SNP GWASs, random forest-based GWASs entail heavy computational burden. Therefore, it is not affordable to assess the statistical significance of the importance measure based on an empirical distribution *H*_0_, to provide a *p*-value. Dealing with *p*-values on the one hand, and importance values on the other hand, our comparative study focused on the 100 top ranked SNPs, for each of the single-SNP, T-Trees, and hybrid FLTM / T-Trees approaches.

## Results and discussion

This section presents and discusses the results obtained following the road map abovedepicted.

### Comparison of the performances of T-Trees and the hybrid approach on simulated data

**Fig. 7:**
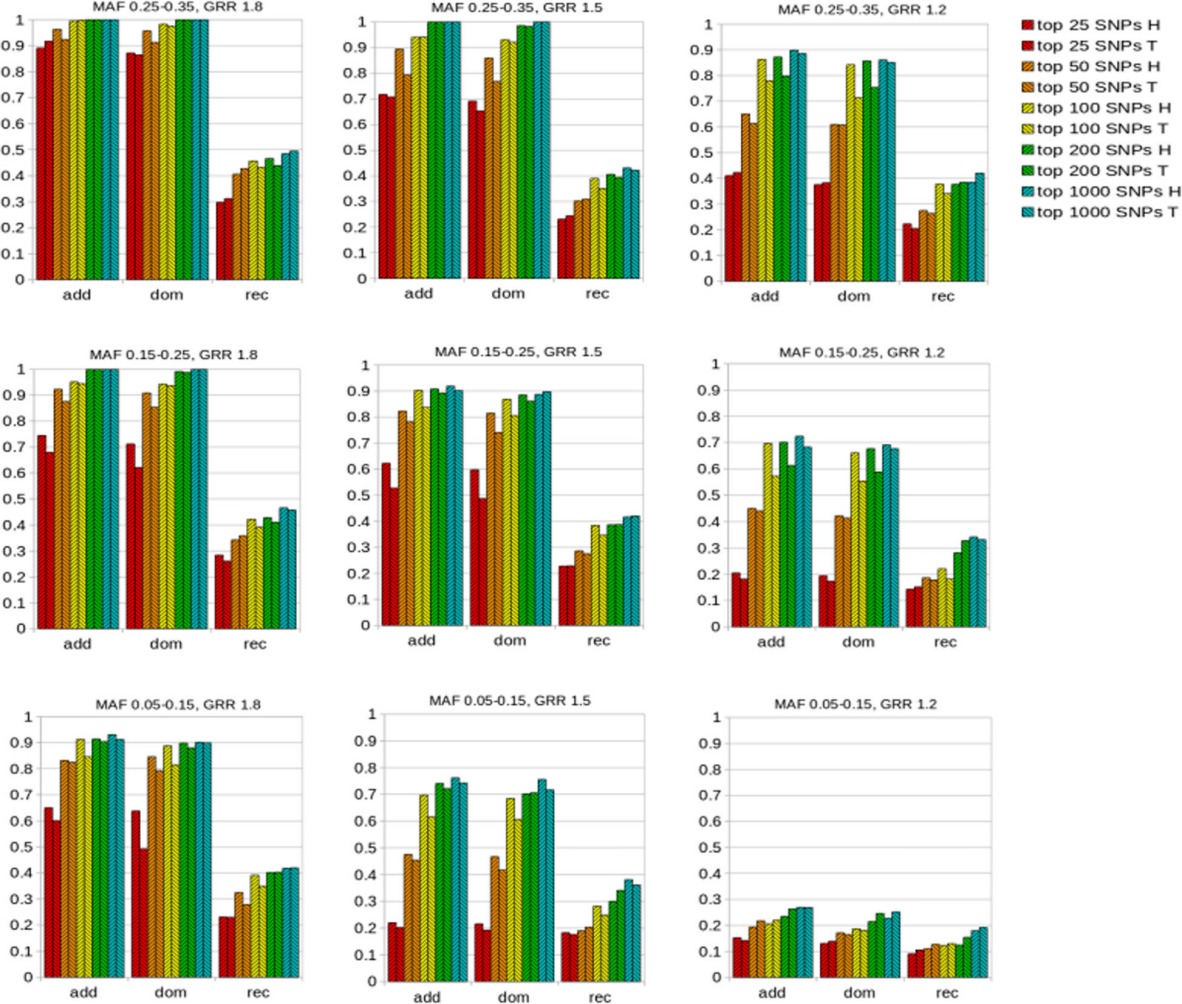
Comparison of the performances of T-Trees and the hybrid approach on simulated data. We computed the percentage of the causal SNPs found among the top results, over all replicates related to the same condition (GM, GRR, MAF). GM: genetic model (add, dom, rec); GRR: genetic relative risk; MAF: minor allele frequency of the causal SNP; H: hybrid approach, T: T-Trees approach. We successively computed this percentage for the top 25, top 50, top 100, top 200 and top 1000 results

Figure 7 allows to compare the performances of T-Trees and the hybrid approach under each of the 27 conditions simulated. For the additive and dominant genetic models, we find that the hybrid approach almost always outperforms T-Trees when small sets of top results are examined. Then, sooner or later, the discrepancy between the methods diminishes. For a first illustration, we examine condition (GM: add, GRR: 1.5, MAF: 0.25-0.35). Regarding the top 25 set, the hybrid approach slightly outperforms T-Trees, with a percentage of simulated causal SNPs retrieved equal to 71.7% (*versus* 70.8% for T-Trees). The discrepancy is higher for the top 50 set, for which the hybrid method is able to retrieve 89.4% of the causal SNPs, in contrast to the relatively low percentage of 79.3% for T-Trees. As from the top 100 set, both methods show quasi similar performances. Under this condition, the top 100 set contains around 94% of the causal SNPs. For a second illustration, we now focus on condition (GM: add, GRR: 1.5, MAF: 0.15-0.25). The percentage of simulated causal SNPs retrieved in the top 25 set is 62.2% for the hybrid method. This percentage is only 52.7% for T-Trees. Both methods present a top 1000 percentage around 90% (respectively 91.0% and 90.2% for the hybrid method and T-Trees). Under this condition, a difference between the two methods exists up to the top 100 set (90.3% *versus* 83.7%).

For the additive and dominant models, we observe a more or less smooth degradation of the performances as the MAF and GRR decrease. However, the hardest case (GRR: 1.2, MAF: 0.05-0.15) is an exception, for which even the top 100 to top 1000 percentages are low (below 30%). In constrast, as expected, in all conditions, the performances are poor for the recessive model.

Regarding the recessive model, the trend is mitigated, with T-Trees performing better than the hybrid approach or *vice versa*. Over the 9 (GRR, MAF) conditions, there are only two cases for which the hybrid approach slightly outperforms T-Trees. In 2 of the most 3 difficult conditions, (GRR: 1.2, MAF: 0.15-0.25) and (GRR: 1.5, MAF: 0.05-0.15), the hybrid approach outperforms T-Trees up to top 100 set included (with the exception of top 25 for the first condition previously mentioned). Table 4 recapitulates the main trends observed.

**Table 4:** Main trends observed in the comparison of the performances of T-Trees and the hybrid approach on simulated data. See Fig. 7 for abbreviations and details

	

As the trends for the additive and dominant genetic models are comparable for each of the 9 conditions simulated (GRR, MAF), we only focus here on the additive and recessive models

To conclude, these first experiments show that combining T-Trees with the modeling of linkage disequilibrium improves the original T-Trees method.

### Comparison of the predictive powers of T-Trees and the hybrid approach on real data

The parallelization of T-Trees allowed to decrease the running time from around 3 days to around 3 hours. Thus, the 10-fold cross-validation time was reduced from around one month to less than 40 hours. The AUCs for T-Trees and the hybrid FLTM / T-Trees approach are displayed in Table 5. The first conclusion to draw is that both methods perform similarly, with, on average, a slight improvement of the hybrid method over T-Trees in 10 cases over 14. Globally, the absolute difference in AUCs between the two methods ranges from 0.6 to 3.0%. For the 10 situations showing improvement, the average improvement amounts to 1.74% and the standard deviation is 0.85%. It was not a foregone result that we could improve the performance of T-Trees as it was already high. We conclude that the sophistication of T-Trees through finer linkage disequilibrium modeling is shown beneficial in the majority for the 14 datasets analyzed.

**Table 5:** Comparison of performances for T-Trees and the hybrid FLTM / T-Trees approach

Pathology	Chromosome	AUC T-Trees	AUC hybrid FLTM /
			T-Trees approach
BD	Chr03	0.928	**0.934**
	Chr21	0.933	**0.958**
CAD	Chr05	**0.953**	0.934
	Chr06	**0.968**	0.947
CD	Chr01	0.943	**0.952**
	Chr20	0.917	**0.944**
HT	Chr10	0.910	**0.940**
	Chr14	0.921	**0.932**
RA	Chr06	**0.957**	0.950
	Chr19	0.946	**0.962**
T1D	Chr02	**0.956**	0.939
	Chr13	0.935	**0.957**
T2D	Chr10	0.942	**0.961**
	Chr21	0.927	**0.936**

The bold face characters highlight the highest AUC observed between T-Trees and the hybrid approach

### Comparison of the distributions of variable importances obtained from T-Trees and the hybrid approach on real data

For a complementary analysis, we compared the distributions of variable importances across the SNPs, obtained from T-Trees and the hybrid FLTM / T-Trees approach. Additional file 1 in Supplementary data provides the results of this thorough analysis. Table 1 in Additional file 1 displays the minimum, maximum, average and standard deviation values obtained for both T-Trees and hybrid approaches. The extract shown in Table 6 is representative of the trend observed over the 14 datasets. For T-Trees, importance measures vary from non-detectable to orders of magnitude of 10^−2^ for 7 datasets and 10^−1^ for the 7 other datasets. As regards the hybrid approach, importance measures vary from non-detectable to orders of magnitude 10^−2^ and 10^−1^ respectively for 6 and 8 datasets. Except for one dataset, (CD, Chr20), the maximal importance values are in the same order of magnitude for both methods. Besides, for T-Trees and the hybrid approach, averages across SNPs are always in the same order of magnitude (10^−5^, exceptionally 10^−4^ for 3 datasets ((BD, Chr21), (RA, Chr19) and (T2D, Chr21)). The orders of magnitude for the standard deviations are in the range [ 10^−4^−10^−3^]. Again, the trend observed is similarity in the orders of magnitude for T-Trees and the hybrid approach, except for 3 datasets ((BD, Chr03), (BD, Chr21) and (CD, Chr20)).

**Table 6:** Range of variation, average and standard deviation for the distributions of variable importances across the SNPs, obtained from T-Trees and the hybrid FLTM / T-Trees approach. Excerpt of Table 1 in Additional file 1 (Supplementary data)

Pathology	Method	Minimum	Maximum	Average	Standard
Chromosome					deviation
TD2, Chr10	T-Trees	0	0.019	3.8e-05	2.5e-04
	hybrid	0	0.089	3.2e-05	6.4e-04
RA, Chr19	T-Trees	0	0.372	1.7e-04	4.9e-03
	hybrid	0	0.391	2.1e-04	5.3e-03
CD, Chr20	T-Trees	0	**0.030**	5.9e-05	**8.6e-05**
	hybrid	0	**0.119**	8.6e-05	**1.2e-03**

Convention : 0 stands for non-detectable at 10^−8^ threshold. The two first datasets are representative of the general trend observed over the 14 datasets analyzed: same order of magnitude for maxima, averages and standard deviations, respectively. Besides, dataset (T2D, Chr10) is among the datasets showing the smallest maxima. Dataset (RA, Chr19) shows the highest maxima. (CD, Chr20) represents the unique case of discrepencies between the orders of magnitude of the maxima (in favor of the hybrid approach). Dataset (CD, Chr20) is also one of the three cases showing a discrepancy in the standard deviations’ orders of magnitude

However, complementary statistics shed more light on the distributions compared. From Table 2 in Additional file 1 (Supplementary data), we observe that the correlation coefficient varies from 0.13 to 0.98. Moreover, the Wilcoxon test rejects the null hypothesis of similarity for the two distributions, in all 14 cases. An extract of this table is shown in Table 7. Since, according to the Wilcoxon test, the mean ranks between the distributions differ, the conclusion to draw is that, in particular, the top SNPs are likely to differ between the two methods compared.

**Table 7:** Wilcoxon rank sum test and Pearson correlation coefficient, to compare the distributions of variable importances across the SNPs, obtained from T-Trees and the hybrid FLTM / T-Trees approach. Excerpt of Table 2 in Additional file 1 (Supplementary data)

Pathology,	Wilcoxon rank sum test	Pearson correlation
chromosome		coefficient
T1D, Chr13	W = 212926668, *p*-value < 2.2e-16	0.9770057
HT, Chr10	W = 442501186, *p*-value < 2.2e-16	0.7079702
CD, Chr20	W = 83225566, *p*-value < 2.2e-16	0.3365107
T1D, Chr02	W = 883236380, *p*-value < 2.2e-16	0.1247845

The four cases shown here encompass the wide range of variation observed for the Pearson correlation coefficient. The Wilcoxon test always indicates that the two distributions are not similar

Tables 3 to 16 in Additional file 1 (Supplementary data) help decipher the origin of the discrepancies between the T-Trees and hybrid approaches’ distributions. For each dataset (pathology, chromosome), the corresponding table compares the 25%, 50%, 75% and 100% quantiles obtained for T-Trees and the hybrid approach. Importantly, a focus is also set on the 6 quantiles that correspond to the top 300, top 200, top 100, top 50, top 20 and top 10 SNPs output by each method. A first observation is that up to the 50% quantile, for both methods, the importance measures vary in the range of from non-detectable (i.e. below 10^−8^) to the order of magnitude 10^−5^ at most (exceptionally 10^−4^ at most for T-Trees and datasets (BD, Chr21) and (RA, Chr19)). We also observe a constant trend: up to 75% quantile included, the hybrid’s method quantiles are always lower than T-Tree’s quantiles. In contrast, for 12 datasets out of 14, the hybrid’s method quantiles are higher than T-Trees’ quantiles as from some threshold quantile. To fix ideas, we show in Table 8 the quantiles relative to dataset (RA, Chr19). In this case, more “important” SNPs are likely to be found in the hybrid’s top 300 SNPs than in the T-Trees’ top 300s (*quantile*_*hybrid*_=1.5×10^−2^*versus* *quantile*_*T*−*Trees*_=6.0×10^−3^).

**Table 8:** Comparison of ten quantiles for the distributions of the variable importances, across the SNPs, for T-Trees and the hybrid FLTM / T-Trees approach. Illustration with the case of Rheumatoid arthritis, chromosome 19

			top300	top200	top100	top50	top20	top10	max
25%	50%	75%	99.99439%	99.99626%	99.99813%	99.99907%	99.99963%	99.99981%	100%
Quantiles T-Trees									
**1.7e-05**	**5.0e-05**	**1.1e-04**	6.0e-03	1.4e-02	2.8e-02	2.0e-01	3.0e-01	3.4e-01	3.7e-01
Quantiles hybrid approach									
1.8e-06	1.7e-05	6.1e-05	**1.5e-02**	**2.6e-02**	**1.0e-01**	**2.5e-01**	**3.3e-01**	**3.6e-01**	**3.9e-01**

The bold face characters highlight the highest value observed between T-Trees and the hybrid approach

As biologists expect a short list of prioritized SNPs to be further analyzed, it is important to study whether one of the two methods yields higher importance values than the other for the top SNPs. Table 9 subsumes the tendencies observed from Tables 3 to 16 in Additional file 1 (Supplementary data).

**Table 9:** Quantile from which the quantiles for one method are always greater than those of the other method, regarding the distributions of variable importances, across the SNPs, for T-Trees and the hybrid FLTM / T-Trees approach. For example, for (T1D, Chr13) dataset, the hybrid quantiles are always greater than the T-Trees quantiles as from top100 quantile. Moreover, the hybrid quantiles are an order of magnitude higher as from top20 quantile

Pathology	Chromosome	Constantly higher as of quantile		One order of magnitude higher as of quantile
		T-Trees	hybrid approach	
BD	Chr03		top200	—
	Chr21		top300	—
CAD	Chr05		top10	—
	Chr06		top100	—
CD	Chr01		top300	—
			(except for top100)	
	Chr20		top200	top200 (except for top100 and top50)
HT	Chr10		top200	—
	Chr14		top300	—
			(except for top100)	
RA	Chr06		top10	—
	Chr19		top300	—
T1D	Chr02	top25%		—
		(except for top300)		
	Chr13		top100	top20
T2D	Chr10		top300	—
	Chr21	top25%		—
		(except for top300 and top200)		

Hintertho we knew that the two distributions of the variable importances differed between T-Trees and the hybrid approach. The conclusion to draw now is that, except for 2 datasets out of 14, the hybrid method outputs top ranked SNPs with relatively higher importances than in T-Trees, which is our focus in GWASs. Therefore, the search space seems to be more efficiently explored. Potentially, the hybrid approach allows us to group the SNPs in clusters in a manner more efficient to target the putative associated SNPs. In T-Trees, artificial blocks of 20 contiguous SNPs attempt to approximate linkage disequilibrium. In the hybrid FLTM / T-Trees approach, clustering based on linkage disequilibrium produces singletons and clusters of pairwise highly correlated SNPs. First, it has to be emphasized that regarding the draws of SNPs contributing to Extra-trees, T-Trees and the hybrid approach are put on an equal footing: *k* was set to either the block’s size (T-Trees) or was dynamically adapted to any cluster’s size (hybrid approach). Thus, in this GWAS framework, any SNP in a block (respectively cluster) will be considered to contribute to the Extra-tree built for this block (respectively cluster). However, in T-Trees, the artificial delimitation into blocks may lessen the chance of testing SNPs from the same real haplotype block within the Extra-tree scope. It is likely that the multivariate split produced from a cluster of SNPs tagging the putative influential SNP, or best capturing association with the phenotype, will be the optimal split (Algorithm 5, line 11), for any such cluster draw (at any meta-node of the *T* meta-trees). In T-Trees, a block is also likely to be drawn at any meta-node of the *T* meta-trees. However, if the SNPs that best tag the influential SNP, or best capture association with the phenotype, are spread over two or more blocks, none of these blocks is likely to produce an optimal multivariate split.

Prediction performance measured through the AUC of a ROC curve relies on data describing both true and predicted statuses for the individuals. We now examine whether, for a given dataset, a method with a higher AUC than the other method (Table 5) would also be the approach showing inflated top SNPs’ importance quantiles (Table 9). Indeed, in 10 cases out of 14, we observe consistency between Tables 5 and 9. Inconsistencies are pinpointed for datasets (CAD, Chr05), (CAD, Chr06), (RA, Chr06) and (T2D, Chr21). First, for 6 out of 10 cases for which the AUC is the highest for the hybrid approach, we observe that the highest importances distribution tail for the hybrid approach is over that of T-Trees as from top200 or top300 quantile. In contrast, when the hybrid approach distribution is over T-Trees’ as only from top10 quantile, we observe a higher AUC for T-Trees (inconsistencies for (CAD, Chr05) and (RA, Chr06)). In the intermediary top quantiles (top100), the situation is mitigated, with dataset (CAD, Chr06) showing the lowest AUC for the hybrid approach (inconsistency) whereas it is the contrary for dataset (T1D, Chr13). To attempt to explain the fourth inconsistency, (T2D, Chr21), we compare this case with that of (T1D, Chr02). In the second case, T-Trees’ importance values are inflated as from top25% quantile (except for top300 quantile) and T-Trees’ AUC is the highest. The inconsistency of dataset (T2D, Chr21) might be explained by the fact that even though T-Trees’s importance values are also inflated as from top25% quantile, top300 and top200 quantiles are missing. However, the explanation is not so simple as it would well explain the inconsistency for (CAD, Chr06) (both inflated distribution as from top100 quantile and lowest AUC for the hybrid approach) but not the consistency of (T1D, Chr13) (inflated distribution as from top100 quantile for the hybrid approach and highest AUC for the hybrid approach). With this latter unique restriction, for the datasets analyzed, we are inclined to draw two conclusions: the method with the highest importance distribution tail would also show the highest predictive power; the importances of the variables in the rank intervals top300 and top200 might be crucial for the method’s predictive power.

An additional conclusion is that both methods can be used to pinpoint various top SNPs, in a complementary way. However, complementarity can also be used to reinforce confidence in top SNPs. In the sequel, we will then focus on the SNPs identified in common by several of the compared methods, including the single-SNP GWAS.

### Analysis of the SNP sets jointly identified by any two among the three, or the three methods compared on real data

**Fig. 8:**
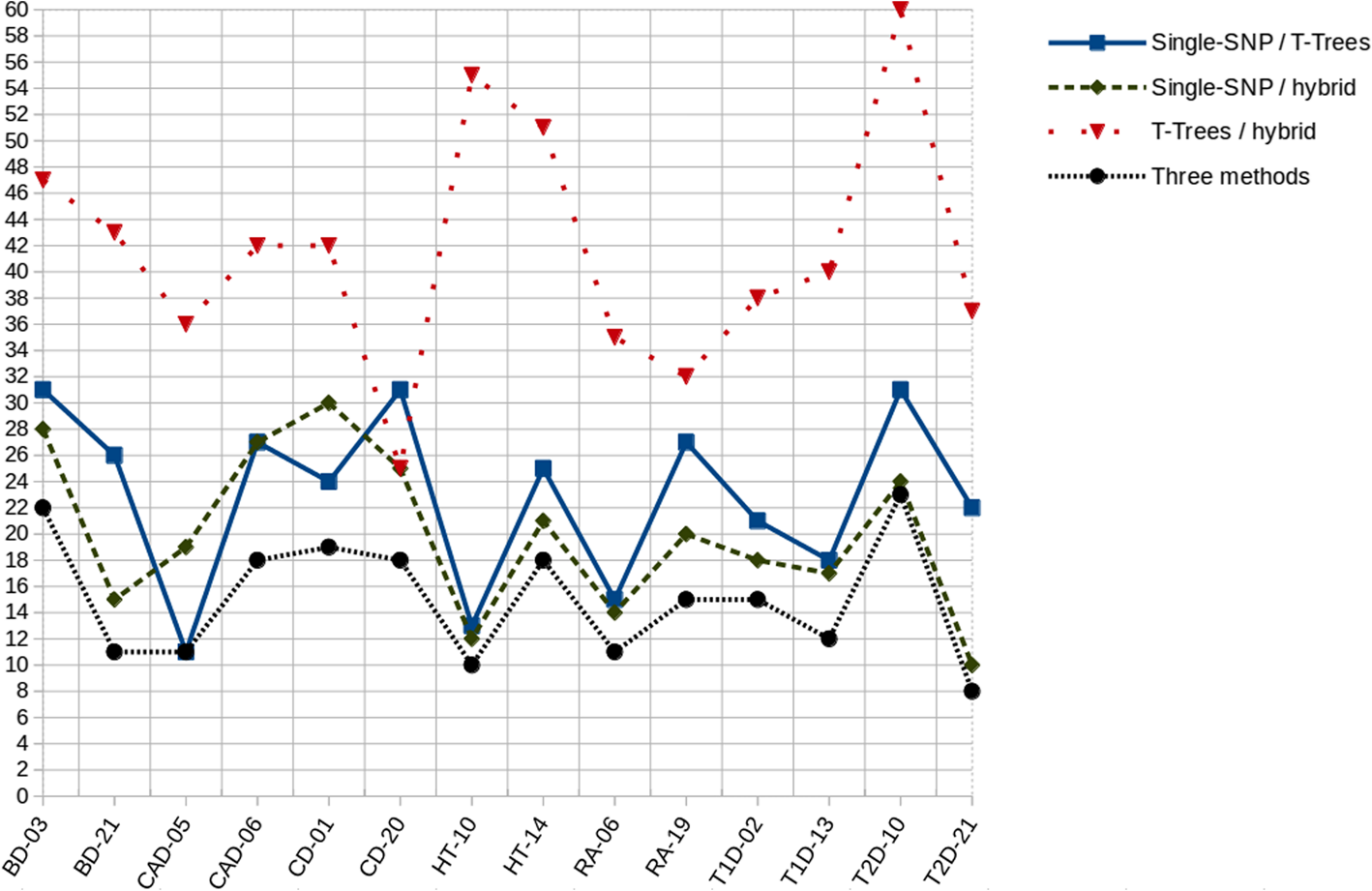
Number of SNPs jointly identified in the top 100s by any two or all three methods studied, for 14 pairs (pathology, chromosome): single-SNP GWAS *versus* T-Trees, single-SNP GWAS *versus* hybrid approach, T-Trees *versus* hybrid approach, and the three methods. The labels on the x-axis represent the pairs (pathology, chromosome number)

**Fig. 9:**
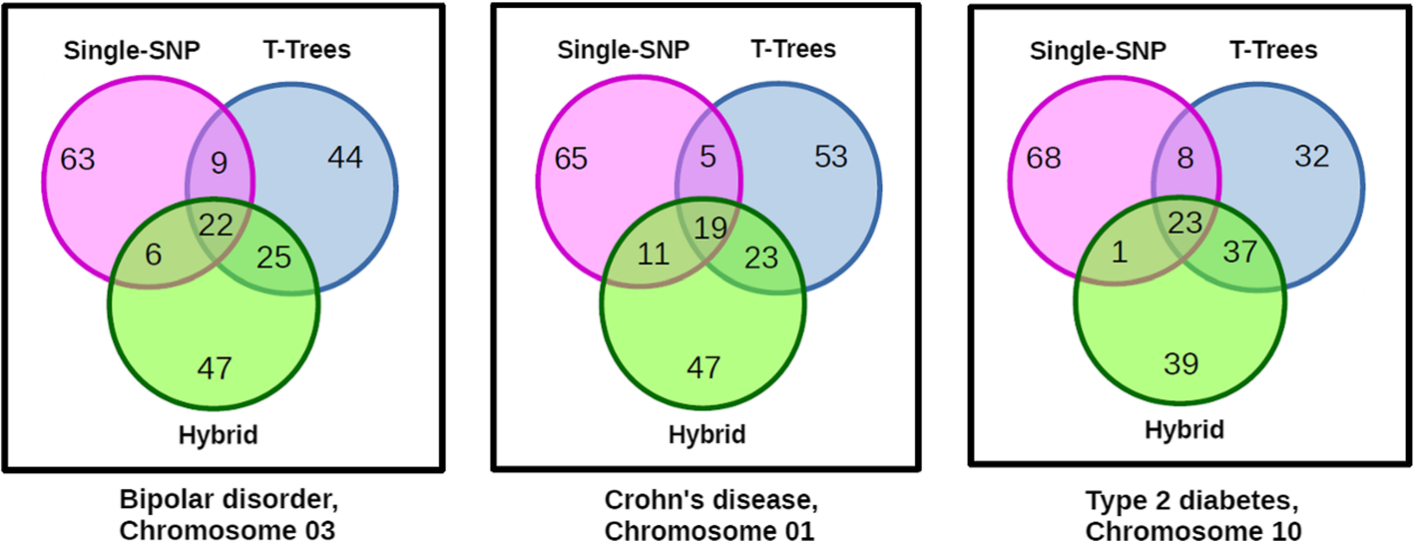
Venn diagrams for three datasets, describing the SNPs jointly identified in the top 100s by any two or all three methods studied: single-SNP GWAS *versus* T-Trees, single-SNP GWAS *versus* hybrid approach, T-Trees *versus* hybrid approach, and the three methods. (T2D, Chr10) is the dataset observed with the highest number of top 100 SNPs common to the three methods (23), over the 14 datasets. Dataset (BD, Chr03) is one of the three datasets that show the largest number of top 100 SNPs common to single-SNP GWAS and T-Trees (31). The largest number of top 100 SNPs common to Single-SNP GWAS and the hybrid approach (30) is observed for (CD, Chr01). Finally, (T2D, Chr10) is the dataset for which the number of top 100 SNPs common to T-Trees and the hybrid approach is the highest (60)

We reported the SNPs jointly identified in the top 100s by any two or all three methods studied : single-SNP GWAS *versus* T-Trees, single-SNP GWAS *versus* hybrid approach, T-Trees *versus* hybrid approach, and the three methods. Additional file 2 (Supplementary data) describes these results for each of the 14 pairs (pathology, chromosome) analyzed. Figure 8 plots the cardinalities of the 3 pairwise intersections and of the three-way intersection for the 14 datasets analyzed. The Venn diagrams in Additional file 3 (Supplementary data) provide an intuitive insight of the trends observed across the 14 datasets analyzed. For an illustration, Fig. 9 focuses on the Venn diagrams of (BD, Chr03), (CD, Chr01) and (T2D, Chr10). (T2D, Chr10) is the dataset observed with the highest number of top 100 SNPs common to the three methods (23), over the 14 datasets. Together with datasets (CD, Chr20) and (T2D, Chr10), dataset (BD, Chr03) shows the largest number of top 100 SNPs common to single-SNP GWAS and T-Trees (31). The largest number of top 100 SNPs common to Single-SNP GWAS and the hybrid approach (30) is observed for (CD, Chr01). Finally, (T2D, Chr10) is again the dataset for which the number of top 100 SNPs common to T-Trees and the hybrid approach is the highest (60). The reader interested in a more thorough analysis of the number of top 100 SNPs common to any or the three methods is reported to the end of Additional file 3.

It was expected that the number of top 100 SNPs common to each T-Tree-based strategy and the Single-SNP GWAS would be smaller than the number of SNPs shared with the other T-Tree-based strategy. Indeed, the two categories of approaches, T-Trees-based and Single-SNP, resort on quite different detection mechanisms. Random forest-based methods are certainly complementary to the Single-SNP approach. On the other hand, as the powers are quite similar and relatively high for T-Trees and the hybrid approach as shown in the section relative to AUC comparison, we can also conclude that both methods are complementary. Any top 100 SNP detected by one of the two T-Trees-based methods is anyway amongst the most relevant to discriminate between cases and controls. Furthermore, a SNP jointly pinpointed by T-Trees and the hybrid approach should be prioritized for further biological analysis. Interestingly, among the latter SNPs, top 100s jointly identified by the three methods must be paid attention to. The number of such SNPs varies in the range [8, 23] over the 14 datasets analyzed.

Table 1 in Additional file 4 (Supplementary data) describes the characteristics of the SNPs jointly identified in the top 100s by each of the three methods. Additional file 4 then discusses these results in details. In the sequel, a SNP jointly identified in the top *n* SNPs by each of the three GWAS strategies is called a top*n SNP. For instance, a SNP identified in the top 20 SNPs by each method is called a top*20 SNP. For each dataset, we are interested in identifying the maximal integer *m* (*m*≤100), such that the top*100 SNPs are also top*m SNPs.

A summary of Additional file 4 is provided in Table 10. We observe that each time a top*1 SNP is detected (that is for datasets (BD,Chr03), (CAD,Chr05), (CD,Chr01), (RA,Chr06), (RA,Chr19) and (T2D,Chr21)), it is significantly associated with the disease, according to the Single-SNP GWAS strategy. Besides, 62 other top*m SNPs are also characterized with a significant *p*-value. In addition, Table 10 allows to prioritize top*100 SNPs for further biological investigation: among the 211 top*100 SNPs detected over the 14 datasets, we identified 72 top*25 SNPs including 38 top*10 SNPs. Thus, an additional insight of our study is the interest to select top*m SNPs to prioritize a list of SNPs.

**Table 10:** Summary of the top 100 SNPs common to single-SNP GWAS, T-Trees and hybrid FLTM / T-Trees approaches. A SNP jointly identified in the top *n* SNPs by each of the three GWAS strategies is called a top*n SNP

	

Finally, Table 11 displays the number of top 100 SNPs common to T-Trees and the hybrid approach. For 8 datasets over 14, the correlation coefficient of the corresponding variable importances is greater than 0.93. Regarding the 6 other datasets, the correlation coefficient varies between 0.58 and 0.87.

**Table 11:** Correlation analysis for the variable importances of the common top 100 SNPs identified by T-Trees and the hybrid FLTM / T-Trees approach

Pathology	Chromosome	Number of common	Correlation
		top 100 SNPs	coefficient
BD	Chr03	47	0.99305
	Chr21	43	0.87334
CAD	Chr05	36	0.99160
	Chr06	42	0.81184
CD	Chr01	42	0.99484
	Chr20	25	0.94999
HT	Chr10	55	0.98335
	Chr14	51	0.58097
RA	Chr06	35	0.93001
	Chr19	32	0.99948
T1D	Chr02	38	0.73068
	Chr13	40	0.97849
T2D	Chr10	60	0.70314
	Chr21	37	0.69802

### Current limitation for applicability of the hybrid approach on a genome-wide scale

The bottleneck to extend the hybrid approach to genome-scale is the FLTM algorithm. In the machine learning domain, learning a latent tree is challenging in the high dimensional case. There exist $O\big (2^{3n^{2}}\big)$ candidate structures to build a latent tree derived from *n* observed variables [39]. Learning the tree structure can only be efficiently addressed through iterative ascending clustering of the variables. Mourad and co-workers examined various such clustering-based approaches and their limitations [40]. In the latter work, 15 methods were compared, including FLTM (named CFHLC in the paper cited) ([40], page 183). FLTM was the method with the highest scalability. On the other hand, it has to be noted that in its very first version (i.e. a window-based version), FLTM was also tested on still larger datasets as in [40] (e.g. describing 100,000 variables [32]). For information, the WTCCC dataset (Crohn’s disease, chromosome 1) describes 37,267 SNPs. It is important to emphasize that the FLTM algorithm used in the present paper does not coerce the latent trees’ structure to binary structure, does not impose a user-defined shared cardinality for all latent variables, and does not require contiguity (on the genome) for the variables to be clustered. A flexible and thus faithful modeling of linkage disequilibrium comes at a cost in the present (yet optimized) version of FLTM: high memory consumption and high running time not allowing scalability to genome scale.

## Conclusions and perspectives

In this paper, we put forth an hybrid approach combining two machine learning models, T-Trees and FLTM, to enhance genome-wide association studies. We compared the performances of the integrated approach and of T-Trees based on simulated realistic genetical data. The integrated approach was shown to perform slightly better than T-Trees for the additive and dominant genetic models. There is no subtantial advantage shown for the recessive model, except in few cases. We then performed a comparative analysis of the predictive powers and SNPs’ scores distributions, for T-Trees and the hybrid FLTM / T-Trees approach, on real datasets. The sophistication of T-Trees through finer linkage disequilibrium modeling derived from FLTM is shown beneficial: on the datasets analyzed, the already high predicted power observed for T-Trees is increased in the majority. The distributions of SNPs’ scores generated by T-Trees and the hybrid approach are shown statistically different. In particular, in a vast majority of cases, the hybrid method outputs top ranked SNPs with relatively higher importances than in T-Trees. Thus are pinpointed more interesting SNPs than in T-Trees, to be provided as a short list of prioritized SNPs, for a further analysis by biologists. Not only did we show that both methods can be used to pinpoint various top SNPs, in a complementary way. Complementarity can also be used to reinforce confidence in top SNPs. Therefore, we analyzed the pairwise and three-way intersections of SNPs ranked in the top 100s, for the standard single-SNP GWAS, T-Trees and the hybrid method. In particular, among the 211 top 100 SNPs jointly detected by the three methods, over the 14 datasets analyzed, we identified 72 and 38 SNPs respectively present in the top25s and top10s for each method.

In future work, we will extend the comparative study of T-Trees and the hybrid approach to more GWAS datasets. In particular, we will examine whether a method with a higher predictive power than the other method also tends to show inflated top SNPs’ importance quantiles. This paper was fully dedicated to the thorough comparison of T-Trees and the FLTM / T-Trees approach. In the future, we plan the design of a vast study, to compare the hybrid approach to a panel of other approaches derived from machine learning such as logistic and penalized regressions, gradient boosting machines, ensemble methods, artificial neural networks, support vector machines and Bayesian network-based analysis. Besides, since the FLTM model is the key to the improvement brought by the hybrid approach over T-Trees, several directions focused on FLTM learning need be explored for future work. At a methodological level, we first plan to study (at a larger scale) whether the choice of the clustering algorithm used in FLTM learning impacts the conclusions of the present study. Second, a challenging perspective for future methodological work is to integrate consensus clustering in FLTM learning, especially as the downstream analysis at stake is a GWAS. Again, we plan to assess whether consensus clustering impacts the conclusion of the present study. Finally, at a technical level, the bottleneck to apply the hybrid approach on a genome-wide scale is the scalability of the FLTM learning algorithm. Efforts will be deployed to break this technological limitation.

## Appendix



## Additional files

Additional file 1 Comparison of the distributions of variable importances across the SNPs, obtained from T-Trees and the hybrid FLTM / T-Trees approach. **Table S1:** Range of variation, average and standard deviation for the distributions of variable importances across the SNPs, obtained from T-Trees and the hybrid FLTM / T-Trees approach. **Table S2:** Wilcoxon rank sum test and Pearson correlation coefficient, to compare the distributions of variable importances across the SNPs, obtained from T-Trees and the hybrid FLTM / T-Trees approach. **Table S3** to **S6:** Comparison of the distributions of the variable importances, across the SNPs, for T-Trees and the hybrid FLTM / T-Trees approach, for the 14 datasets analyzed. (PDF 67.8 kb)

Additional file 2 SNPs jointly identified in the top 100s by two or three methods among single-SNP, T-Trees and hybrid FLTM / T-Trees approaches. For each of the 14 datasets analyzed, 4 tables provide the ranks, variable importances and *p*-values of the SNPs in the top 100s jointly identified by: Single-SNP and T-Trees approaches; Single-SNP and hybrid approaches; T-Trees and hybrid approaches; Single-SNP, T-Trees and hybrid approaches. (PDF 202 kb)

Additional file 3 SNPs jointly identified in the top 100s by two or three methods among single-SNP, T-Trees and hybrid FLTM / T-Trees approaches. Venn diagrams. For each of the 14 datasets analyzed, a Venn diagram shows the number of top 100 SNPs jointly identified by any two among the three, or the three methods considered. (PDF 122 kb)

Additional file 4 Characteristics of the SNPs jointly identified as top 100s by the Single-SNP, T-Trees and hybrid FLTM / T-Trees approaches. Table 1: Characteristics of the SNPs jointly identified as top 100s by the Single-SNP, T-Trees and hybrid FLTM / T-Trees approaches. (PDF 81.6 kb)

## Abbreviations

AUC: Aera under the curve
DNA: Deoxyribonucleic acid
EM: Expectation-maximization
FLTM: Forest of latent tree models
GM: Genetic model
GRR: Genetic relative risk
GWAS: Genome-wide association study
LCM: Latent class model
MAF: Minor allele frequency
ROC: Receiver operator characteristic
SNP: Single nucleotide polymorphism
WTCCC: Wellcome trust case control consortium
