# Supplementary material for: A method combining a random forest-based technique with the modeling of linkage disequilibrium through latent variables, to run multilocus genome-wide association studies
Source: BMC Bioinformatics. 2018 Mar 27;19:106. doi: 10.1186/s12859-018-2054-0 (PMC5870262; doi:10.1186/s12859-018-2054-0)
Supplement: Supplementary file 1 — Comparison of the distributions of variable importances across the SNPs, obtained from T-Trees and the hybrid FLTM / T-Trees approach. Table S1: Range of variation, average and standard deviation for the distributions of variable importances across the SNPs, obtained from T-Trees and the hybrid FLTM / T-Trees approach. Table S2: Wilcoxon rank sum test and Pearson correlation coefficient, to compare the distributions of variable importances across the SNPs, obtained from T-Trees and the hybrid FLTM / T-Trees approach. Table S3 to S6: Comparison of the distributions of the variable importances, across the SNPs, for T-Trees and the hybrid FLTM / T-Trees approach, for the 14 datasets analyzed. (PDF 67.8 kb) [file 12859_2018_2054_MOESM1_ESM.pdf]

Supplementary data
Additional file 1

Comparison of the distributions of variable importances across the SNPs, obtained from T-Trees and the hybrid FLTM / T-Trees approach.

Table 1. Range of variation, average and standard deviation for the distributions of variable importances across the SNPs, obtained from T-Trees and the hybrid FLTM / T-Trees approach.

		min	max	avg	std
BD Chr03	T-Trees	0	0.156	3.2e-05	**9.3e-04**
	hybrid	0	0.178	2.2e-05	**1.1e-03**
BD Chr21	T-Trees	0	0.038	1.5e-04	**8.4e-04**
	hybrid	0	0.073	2.4e-04	**1.5e-03**
CAD Chr05	T-Trees	0	0.257	2.8e-05	2.0e-03
	hybrid	0	0.262	4.1e-05	2.0e-03
CAD Chr06	T-Trees	0	0.040	3.6e-05	3.9e-04
	hybrid	0	0.086	3.0e-05	6.2e-04
CD Chr01	T-Trees	0	0.223	2.7e-05	1.5e-03
	hybrid	0	0.228	1.9e-05	1.5e-03
CD Chr20	T-Trees	0	**0.030**	5.9e-05	**3.5e-04**
	hybrid	0	**0.119**	8.6e-05	**1.2e-03**
HT Chr10	T-Trees	0	0.084	3.3e-05	5.5e-04
	hybrid	0	0.087	2.9e-05	7.8e-04
HT Chr14	T-Trees	0	0.065	6.5e-05	1.0e-03
	hybrid	0	0.095	6.8e-05	1.0e-03
RA Chr06	T-Trees	0	0.159	2.8e-05	1.6e-03
	hybrid	0	0.171	3.6e-05	1.5e-03
RA Chr19	T-Trees	0	0.372	1.7e-04	4.9e-03
	hybrid	0	0.391	2.1e-04	5.3e-03
T1D Chr02	T-Trees	0	0.077	2.3e-05	5.7e-04
	hybrid	0	0.059	2.6e-05	3.7e-04
T1D Chr13	T-Trees	0	0.282	5.2e-05	2.9e-03
	hybrid	0	0.373	4.6e-05	3.7e-03
T2D Chr10	T-Trees	0	0.019	3.8e-05	2.5e-04
	hybrid	0	0.089	3.2e-05	6.4e-04
T2D Chr21	T-Trees	0	0.247	2.7e-04	3.8e-03
	hybrid	0	0.211	1.5e-04	3.1e-03

Convention : 0 stands for non detectable at 10^{-8} threshold.
The discrepancies between the orders of magnitude are highlighted in bold.

Table 2. Wilcoxon rank sum test and Pearson correlation coefficient, to compare the distributions of variable importances across the SNPs, obtained from T-Trees and the hybrid FLTM / T-Trees approach.

	Wilcoxon rank sum test	Pearson correlation coefficient
BD, Chr03	W = 633282423, p-value < 2.2e-16	**0.9146631**
BD, Chr21	W = 27963038, p-value < 2.2e-16	0.6908916
CAD, Chr05	W = 501406014, p-value < 2.2e-16	**0.9768666**
CAD, Chr06	W = 474691834, p-value < 2.2e-16	0.4090784
CD, Chr01	W = 818447416, p-value < 2.2e-16	**0.9732679**
CD, Chr20	W = 83225566, p-value < 2.2e-16	0.3365107
HT, Chr10	W = 442501186, p-value < 2.2e-16	**0.7079702**
HT, Chr14	W = 134413060, p-value < 2.2e-16	0.5400283
RA, Chr06	W = 476388077, p-value < 2.2e-16	**0.9326264**
RA, Chr19	W = 22488098, p-value < 2.2e-16	**0.9647289**
T1D, Chr02	W = 883236380, p-value < 2.2e-16	0.1247845
T1D, Chr13	W = 212926668, p-value < 2.2e-16	**0.9770057**
T2D, Chr10	W = 459024044, p-value < 2.2e-16	0.4181679
T2D, Chr21	W = 27173084, p-value < 2.2e-16	**0.7188555**

The Pearson correlation coefficients over 0.70 are highlighted in bold.

Conventions:

T-Trees quantile higher than hybrid approach quantile		Hybrid approach quantile higher than T-Trees quantile	
At least one order of magnitude higher for T-Trees			At least one order of magnitude higher for hybrid approach

Table 3. Comparison of ten quantiles for the distributions of the variable importances, across the SNPs, for T-Trees and the hybrid FLTM / T-Trees approach. Bipolar Disorder, Chromosome 03.

				top300	top200	top100	top50	top20	top10	max
BD Chr03	25%	50%	75%	99.99049%	99.99366%	99.99683%	99.99842%	99.99937%	99.99968%	100%
	Quantiles T-Trees									
	2.0e-06	**7.8e-06**	**2.0e-05**	**2.2e-02**	2.4e-02	2.7e-02	9.2e-02	1.3e-01	1.4e-01	1.4e-01
	Quantiles hybrid approach									
	0	2.4e-06	1.3e-05	1.7e-02	**3.0e-02**	**6.8e-02**	***1.2e-01***	**1.6e-01**	**1.7e-01**	**1.8e-01**

Table 4. Comparison of ten quantiles for the distributions of the variable importances, across the SNPs, for T-Trees and the hybrid FLTM / T-Trees approach. Bipolar Disorder, Chromosome 21.

				top300	top200	top100	top50	top20	top10	max
BD Chr21	25%	50%	75%	99.95485%	99.9699%	99.98495%	99.99248%	99.99699%	99.9985%	100%
	Quantiles T-Trees									
	2.3e-05	**6.1e-05**	***1.4 e-04***	1.3e-02	2.4e-02	3.61e-02	3.69e-02	3.73e-02	3.75e-02	3.76e-02
	Quantiles hybrid approach									
	2.1e-06	2.5e-05	9.8e-05	**2.9e-02**	**4.1e-02**	**7.315e-02**	**7.323e-02**	**7.329e-02**	**7.330e-02**	**7.332e-02**

Table 5. Comparison of ten quantiles for the distributions of the variable importances, across the SNPs, for T-Trees and the hybrid FLTM / T-Trees approach. Coronary Artery Disease, Chromosome 05.

				top300	top200	top100	top50	top20	top10	max
CAD Chr05	25%	50%	75%	99.98998%	99.99332%	99.99666%	99.99833%	99.99933%	99.99967 %	100%
	Quantiles T-Trees									
	0	***2.2e-06***	**7.4e-06**	5.0e-02	**1.4e-01**	**1.7e-01**	**2.1e-01**	**2.40e-01**	2.49e-01	2.57e-01
	Quantiles hybrid approach									
	0	6.8e-07	6.9e-06	**5.9e-02**	1.3e-01	1.5e-01	2.0e-01	2.39e-01	**2.50e-01**	**2.62e-01**

Table 6. Comparison of ten quantiles for the distributions of the variable importances, across the SNPs, for T-Trees and the hybrid FLTM / T-Trees approach. Coronary artery disease, Chromosome 06.

				top300	top200	top100	top50	top20	top10	max
CAD Chr06	25%	50%	75%	99.98932%	99.99288%	99.99644%	99.99822%	99.99929%	99.99964%	100%
	Quantiles T-Trees									
	2.2e-06	**9.8e-06**	**2.7e-05**	**1.6e-02**	**2.5e-02**	3.2e-02	3.6e-02	3.81e-02	3.89e-02	4.0e-02
	Quantiles hybrid approach									
	0	4.3e-06	2.0e-05	1.3e-02	1.4e-02	**4.3e-02**	**6.4e-02**	**7.8e-02**	**8.2e-02**	**8.6e-02**

Table 7. Comparison of ten quantiles for the distributions of the variable importances, across the SNPs, for T-Trees and the hybrid FLTM / T-Trees approach. Crohn's disease, Chromosome 01.

				top300	top200	top100	top50	top20	top10	max
CD Chr01	25%	50%	75%	99.99195%	99.99463%	99.99732%	99.99866%	99.99946%	99.99973%	100%
	Quantiles T-Trees									
	0	***2.4e-06***	**8.2e-06**	3.1e-02	5.0e-02	**1.49e-01**	1.860e-01	2.08e-01	2.15e-01	2.23e-01
	Quantiles hybrid approach									
	0	4.1e-09	6.1e-06	**5.8e-02**	**6.2e-02**	1.46e-01	**1.867e-01**	**2.11e-01**	**2.20e-01**	**2.28e-01**

Table 8. Comparison of ten quantiles for the distributions of the variable importances, across the SNPs, for T-Trees and the hybrid FLTM / T-Trees approach. Crohn's disease, Chromosome 20.

				top300	top200	top100	top50	top20	top10	max
CD Chr20	25%	50%	75%	99.97411%	99.98274%	99.99137%	99.99568%	99.99827%	99.99914%	100%
	Quantiles T-Trees									
	1.4e-05	**4.2e-05**	**9.6e-05**	**9.2e-03**	9.3e-03	1.0e-02	2.0e-02	2.6e-02	2.8e-02	3.0e-02
	Quantiles hybrid approach									
	1.1e-07	2.0e-05	6.8e-05	7.6e-03	***1.8e-02***	**4.5-02**	**8.2e-02**	***1.0e-01***	***1.1e-01***	***1.2e-01***

Table 9. Comparison of ten quantiles for the distributions of the variable importances, across the SNPs, for T-Trees and the hybrid FLTM / T-Trees approach. Hypertension, Chromosome 10.

HT Chr10				top300	top200	top100	top50	top20	top10	max
	25%	50%	75%	99.98874%	99.99249%	99.99625%	99.99812%	99.99925%	99.99962%	100%
	Quantiles T-Trees									
	2.8e-06	***1.2e-05***	**3.0e-05**	***9.9e-03***	1.17e-02	1.6e-02	5.0e-02	7.1e-02	7.7e-02	8.4e-02
	Quantiles hybrid approach									
	0	4.2e-06	2.0e-05	1.1e-02	**1.22e-02**	**8.654e-02**	**8.657e-02**	**8.6585e-02**	**8.6590e-02**	**8.6596e-02**

Table 10. Comparison of ten quantiles for the distributions of the variable importances, across the SNPs, for T-Trees and the hybrid FLTM / T-Trees approach. Hypertension, Chromosome 14.

HT Chr14				top300	top200	top100	top50	top20	top10	max
	25%	50%	75%	99.97951%	99.98634%	99.99317%	99.99658%	99.99863%	99.99932%	100%
	Quantiles T-Trees									
	3.7e-06	***1.3e-05***	**3.4e-05**	3.2e-02	3.7e-02	**6.0e-02**	6.2e-02	6.36e-02	6.40e-02	6.45e-02
	Quantiles hybrid approach									
	0	5.2e-06	2.2e-05	**4.4e-02**	**5.815e-02**	5.821e-02	**7.6e-02**	**8.7e-02**	**9.1e-02**	**9.5e-02**

Table 11. Comparison of ten quantiles for the distributions of the variable importances, across the SNPs, for T-Trees and the hybrid FLTM / T-Trees approach. Rheumatoid arthritis, Chromosome 06.

RA Chr06				top300	top200	top100	top50	top20	top10	max
	25%	50%	75%	99.98932%	99.99288%	99.99644%	99.99822%	99.99929%	99.99964%	100%
	Quantiles T-Trees									
	0	***1.6e-06***	**7.5e-06**	6.3e-02	**8.4e-02**	1.50e-01	**1.54e-01**	**1.57e-01**	1.58e-01	1.59e-01
	Quantiles hybrid approach									
	0	0	4.2e-06	**7.0e-02**	7.3e-02	***9.1e-02***	1.3e-01	1.55e-01	**1.63e-01**	**1.7e-01**

Table 12. Comparison of ten quantiles for the distributions of the variable importances, across the SNPs, for T-Trees and the hybrid FLTM / T-Trees approach. Rheumatoid arthritis, Chromosome 19.

				top300	top200	top100	top50	top20	top10	max
RA Chr19	25%	50%	75%	99.94867%	99.96578%	99.98289%	99.99145%	99.99658%	99.99829%	100%
	Quantiles T-Trees									
	1.7e-05	**5.0e-05**	***1.1e-04***	6.0e-03	1.4e-02	2.8e-02	2.0e-01	3.0e-01	3.4e-01	3.7e-01
	1.8e-06	1.7e-05	6.1e-05	***1.5e-02***	**2.6e-02**	***1.0e-01***	**2.5e-01**	**3.3e-01**	**3.6e-01**	**3.9e-01**

Table 13. Comparison of ten quantiles for the distributions of the variable importances, across the SNPs, for T-Trees and the hybrid FLTM / T-Trees approach. Type 1 diabetes, Chromosome 02.

				top300	top200	top100	top50	top20	top10	max
T1D Chr02	25%	50%	75%	99.99225%	99.99484%	99.99742%	99.99871%	99.99948%	99.99974%	100%
	Quantiles T-Trees									
	1.10e-06	**6.8e-06**	**1.9e-05**	1.4e-02	**2.3e-02**	**7.1e-02**	**7.4e-02**	**7.53e-02**	**7.58e-02**	**7.7e-02**
	Quantiles hybrid approach									
	0	2.4e-06	1.71e-05	**1.73e-02**	1.85e-02	1.96-02	3.9e-02	5.1e-02	5.5e-02	5.9e-02

Table 14. Comparison of ten quantiles for the distributions of the variable importances, across the SNPs, for T-Trees and the hybrid FLTM / T-Trees approach. Type 1 diabetes, Chromosome 13.

				top300	top200	top100	top50	top20	top10	max
T1D Chr13	25%	50%	75%	99.98333%	99.98889%	99.99444%	99.99722%	99.99889%	99.99944%	100%
	Quantiles T-Trees									
	5.7e-07	***4.4e-06***	***1.3e-05***	**4.4e-02**	**5.2e-02**	2.5e-01	2.68e-01	2.77e-02	2.79e-02	2.82e-02
	Quantiles hybrid approach									
	0	1.1e-08	6.3e-06	2.2e-02	2.8e-02	**3.2e-01**	**3.5e-01**	***3.62e-01***	***3.67e-01***	***3.7e-01***

Table 15. Comparison of ten quantiles for the distributions of the variable importances, across the SNPs, for T-Trees and the hybrid FLTM / T-Trees approach. Type 2 diabetes, Chromosome 10.

T2D Chr10				top300	top200	top100	top50	top20	top10	max
	25%	50%	75%	99.98874 %	99.99249%	99.99625%	99.99812%	99.99925%	99.99962%	100%
	Quantiles T-Trees									
	3.9e-06	***1.4e-05***	**3.4e-05**	1.242e-02	1.247e-02	1.5e-02	1.65e-02	1.78e-02	1.82e-02	1.9e-02
	Quantiles hybrid approach									
	0	4.1e-06	2.15e-05	**1.7e-02**	**2.247e-02**	**2.251e-02**	**5.6e-02**	**7.6e-02**	**8.2e-02**	**8.9e-02**

Table 16. Comparison of ten quantiles for the distributions of the variable importances, across the SNPs, for T-Trees and the hybrid FLTM / T-Trees approach. Type 2 diabetes, Chromosome 21.

T2D Chr21				top300	top200	top100	top50	top20	top10	max
	25%	50%	75%	99.95485%	99.9699%	99.98495%	99.99248%	99.99699%	99.9985%	100%
	Quantiles T-Trees									
	1.2e-05	**3.4e-05**	**7.8e-05**	1.8e-02	4.5e-02	***1.8e-01***	**2.2e-01**	**2.3e-01**	**2.41e-01**	**2.47e-01**
	Quantiles hybrid approach									
	3.4e-07	1.6e-05	5.9e-05	**6.3e-02**	**6.4e-02**	7.7e-02	1.4e-01	1.8e-01	2.0e-01	2.1e-01
