# Supplementary material for: A method combining a random forest-based technique with the modeling of linkage disequilibrium through latent variables, to run multilocus genome-wide association studies
Source: BMC Bioinformatics. 2018 Mar 27;19:106. doi: 10.1186/s12859-018-2054-0 (PMC5870262; doi:10.1186/s12859-018-2054-0)
Supplement: Supplementary file 2 — SNPs jointly identified in the top 100s by two or three methods among single-SNP, T-Trees and hybrid FLTM / T-Trees approaches. For each of the 14 datasets analyzed, 4 tables provide the ranks, variable importances and p-values of the SNPs in the top 100s jointly identified by: Single-SNP and T-Trees approaches; Single-SNP and hybrid approaches; T-Trees and hybrid approaches; Single-SNP, T-Trees and hybrid approaches. (PDF 202 kb) [file 12859_2018_2054_MOESM2_ESM.pdf]

Supplementary data
Additional file 2

SNPs jointly identified in the top 100s by two or three methods among Single-SNP, T-Trees and hybrid FLTM / T-Trees approaches

Conventions:
For each approach, the SNP's rank among the top 100s is indicated.
p : p-value (corrected for multiple testing)
vi: variable importance.

1 Bipolar disorder, Chromosome 03

Table 1.1: Bipolar disorder, Chromosome 03. Single-SNP and T-Trees approaches.

SNP identifier	Location	single-SNP; rank	T-Trees; rank	single-SNP; p	T-Trees; vi
rs7653441	173374426	1	1	0	0.1561931011
rs3762678	133781248	2	4	0	0.0220474069
rs7628245	53247984	3	7	0	0.0112056913
rs3845903	66600360	4	3	0	0.0237618665
rs17046143	6848446	5	8	0.001	0.0099882401
rs17064749	61267686	6	39	0.008	0.0012765635
rs2202154	26393098	7	6	0.022	0.0114248320
SNP A-1957995	171063896	11	16	0.054	0.0044285995
rs2886966	62908574	13	41	0.058	0.0012622906
rs2019695	42006698	17	24	0.181	0.0023423896
rs711715	22985394	22	72	0.57	0.0007964621
rs711716	22985426	23	96	0.621	0.0005592881
rs33457	42378384	24	51	0.638	0.0009790758
rs16827563	158581672	25	56	0.65	0.0009621968
rs295470	140696593	26	87	0.692	0.0006224029
rs1872108	130722662	27	19	0.722	0.0034621434
rs10512862	133975632	28	36	0.795	0.0014089558
SNP A-2224409	123890289	31	89	0.848	0.0006170623
rs1485171	7626185	35	33	0.888	0.0015581042
rs17015351	77696778	36	30	0.915	0.0016450010
rs11920711	85585246	36	76	0.915	0.0007795188
rs33470	42383247	38	40	0.921	0.0012673112
rs10212203	80686487	39	31	0.921	0.0016212017
rs6805662	133942594	44	59	0.977	0.0009089244
rs6784028	134362402	46	88	0.986	0.0006179218
rs950443	71677160	49	69	0.994	0.0008427814
rs16863584	152928404	51	38	0.999	0.0013211409
rs507595	189125115	57	65	1	0.0008668038
rs1451518	32143653	73	47	1	0.0011227261
rs17786145	1480223	80	14	1	0.0048801424
rs13325263	66585897	83	9	1	0.0088900498

Table 1.2: Bipolar disorder, Chromosome 03. Single-SNP and hybrid approaches.

SNP identifier	Location	single-SNP; rank	hybrid; rank	single-SNP; p	hybrid; vi
rs7653441	173374426	1	1	0	0.1783291204
rs3762678	133781248	2	3	0	0.0303686009
rs7628245	53247984	3	7	0	0.0119339000
rs3845903	66600360	4	5	0	0.0150942910
rs17046143	6848446	5	6	0.001	0.0128369544
rs17064749	61267686	6	25	0.008	0.0032611066
rs2202154	26393098	7	4	0.022	0.0173279873
SNP A-1957995	171063896	11	23	0.054	0.0033596859
rs2886966	62908574	13	22	0.058	0.0034646266
rs2019695	42006698	17	47	0.181	0.0016284082
rs13074575	61558323	20	45	0.364	0.0016865986
rs9790193	125504913	21	38	0.387	0.0021321231
rs711715	22985394	22	88	0.57	0.0008602317
rs711716	22985426	23	48	0.621	0.0015439494
rs33457	42378384	24	68	0.638	0.0011299990
rs16827563	158581672	25	33	0.65	0.0026326921
rs295470	140696593	26	27	0.692	0.0031705375
rs1872108	130722662	27	58	0.722	0.0013304489
SNP A-2224409	123890289	31	79	0.848	0.0009709353
rs1485171	7626185	35	41	0.888	0.0019687488
rs17015351	77696778	36	57	0.915	0.0013339900
rs11920711	85585246	36	74	0.915	0.0010162714
rs10212203	80686487	39	52	0.921	0.0014675830
rs9290197	164227902	47	31	0.992	0.0027097052
rs9840380	152130086	62	40	1	0.0019877280
rs17205536	121308058	71	71	1	0.0010879324
rs17786145	1480223	80	9	1	0.0090698610
rs4447759	61555952	91	75	1	0.0010059063

Table 1.3: Bipolar disorder, Chromosome 03. T-Trees and hybrid approaches.

SNP identifier	Location	T-Trees; rank	hybrid; rank	T-Trees; vi	hybrid; vi
rs7653441	173374426	1	1	0.1561931011	0.1783291204
rs3845903	66600360	3	5	0.0237618665	0.0150942910
rs3762678	133781248	4	3	0.0220474069	0.0303686009
rs17193526	1475657	5	10	0.0163705796	0.0087365669
rs2202154	26393098	6	4	0.0114248320	0.0173279873
rs7628245	53247984	7	7	0.0112056913	0.0119339000
rs17046143	6848446	8	6	0.0099882401	0.0128369544
rs4856942	66602107	10	8	0.0067539560	0.0116956781
rs6809441	41469609	11	16	0.0060041387	0.0053272464
rs9864703	173371149	12	13	0.0057369068	0.0068450912
rs9859908	20464090	13	17	0.0054145750	0.0048949921
rs17786145	1480223	14	9	0.0048801424	0.0090698610
SNP A-1957995	171063896	16	23	0.0044285995	0.0033596859
rs1520050	20475108	17	14	0.0042827236	0.0061918899
rs1872108	130722662	19	58	0.0034621434	0.0013304489
rs7646760	53253693	20	19	0.0034251103	0.0046366975
rs13071246	73181413	21	12	0.0033859151	0.0071441128
rs939298	20452080	22	28	0.0029788743	0.0031388968
rs9843730	2900015	23	51	0.0024875827	0.0014759155
rs2019695	42006698	24	47	0.0023423896	0.0016284082
rs17019325	27093521	25	86	0.0020697947	0.0008744696
rs17046163	6863178	29	29	0.0017087791	0.0031264488
rs17015351	77696778	30	57	0.0016450010	0.0013339900
rs10212203	80686487	31	52	0.0016212017	0.0014675830
rs711714	22980154	32	24	0.0015660816	0.0033452713
rs1485171	7626185	33	41	0.0015581042	0.0019687488
rs5000487	96664292	37	46	0.0013686018	0.0016691328
rs17064749	61267686	39	25	0.0012765635	0.0032611066
rs2886966	62908574	41	22	0.0012622906	0.0034646266
rs17039498	1471493	43	44	0.0012076728	0.0017241232
rs33457	42378384	51	68	0.0009790758	0.0011299990
rs4857010	67350387	54	30	0.0009652780	0.0029109917
rs16827563	158581672	56	33	0.0009621968	0.0026326921
rs1456135	152131687	57	32	0.0009413176	0.0026597443
rs2929343	19956418	60	39	0.0009005791	0.0020570015
rs7630176	159312647	61	96	0.0008990264	0.0007780378
rs6599155	41464608	67	11	0.0008587607	0.0083089712
rs2693546	159385669	70	69	0.0008066451	0.0011138469
rs10049161	125419029	71	67	0.0008030398	0.0011500934
rs711715	22985394	72	88	0.0007964621	0.0008602317
rs1159226	17957322	75	42	0.0007811895	0.0019450430
rs11920711	85585246	76	74	0.0007795188	0.0010162714
rs4683558	142309524	78	34	0.0007206566	0.0025873704
rs1045960	73200525	83	43	0.0006598702	0.0017674235
rs295470	140696593	87	27	0.0006224029	0.0031705375
SNP A-2224409	123890289	89	79	0.0006170623	0.0009709353
rs711716	22985426	96	48	0.0005592881	0.0015439494

Table 1.4: Bipolar disorder, Chromosome 03. Single-SNP, T-Trees and hybrid approaches.

SNP identifier	Location	single-SNP; rank	T-Trees; rank	hybrid; rank	single-SNP; p	T-Trees; vi	hybrid; vi
rs7653441	173374426	1	1	1	0	0.1561931011	0.1783291204
rs3762678	133781248	2	4	3	0	0.0220474069	0.0303686009
rs7628245	53247984	3	7	7	0	0.0112056913	0.0119339000
rs3845903	66600360	4	3	5	0	0.0237618665	0.0150942910
rs17046143	6848446	5	8	6	0.001	0.0099882401	0.0128369544
rs17064749	61267686	6	39	25	0.008	0.0012765635	0.0032611066
rs2202154	26393098	7	6	4	0.022	0.0114248320	0.0173279873
SNP A-1957995	171063896	11	16	23	0.054	0.0044285995	0.0033596859
rs2886966	62908574	13	41	22	0.058	0.0012622906	0.0034646266
rs2019695	42006698	17	24	47	0.181	0.0023423896	0.0016284082
rs711715	22985394	22	72	88	0.57	0.0007964621	0.0008602317
rs711716	22985426	23	96	48	0.621	0.0005592881	0.0015439494
rs33457	42378384	24	51	68	0.638	0.0009790758	0.0011299990
rs16827563	158581672	25	56	33	0.65	0.0009621968	0.0026326921
rs295470	140696593	26	87	27	0.692	0.0006224029	0.0031705375
rs1872108	130722662	27	19	58	0.722	0.0034621434	0.0013304489
SNP A-2224409	123890289	31	89	79	0.848	0.0006170623	0.0009709353
rs1485171	7626185	35	33	41	0.888	0.0015581042	0.0019687488
rs17015351	77696778	36	30	57	0.915	0.0016450010	0.0013339900
rs11920711	85585246	36	76	74	0.915	0.0007795188	0.0010162714
rs10212203	80686487	39	31	52	0.921	0.0016212017	0.0014675830
rs17786145	1480223	80	14	9	1	0.0048801424	0.0090698610

2 Bipolar disorder, Chromosome 21

Table 2.1: Bipolar disorder, Chromosome 21. Single-SNP and T-Trees approaches.

SNP identifier	Location	single-SNP; rank	T-Trees; rank	single-SNP; p	T-Trees; vi
rs16997735	39792304	1	1	0	0.0376263891
rs2837588	40669929	2	4	0.002	0.0130988202
rs7276641	25799569	3	5	0.019	0.0121321338
rs13340018	31477667	4	11	0.201	0.0080232522
rs2830549	27126375	5	27	0.248	0.0028724985
rs16987645	31349103	6	61	0.367	0.0012578748
rs980184	39978208	8	58	0.499	0.0012963971
rs999789	39348321	12	3	0.616	0.0239925579
rs9980699	34421519	16	38	0.725	0.0020291073
rs2833256	31366356	18	100	0.908	0.0008349837
rs9980970	17252547	21	42	0.966	0.0018001581
rs11910142	35738222	36	41	1	0.0018476841
rs724070	33353710	40	21	1	0.0043161571
rs16999738	40702268	45	77	1	0.0010315686
rs455304	40886245	46	30	1	0.0026384978
rs2834630	35048509	47	16	1	0.0049274373
rs403746	39392945	54	12	1	0.0079746198
rs2250910	28725372	55	79	1	0.0009855049
rs229040	27129261	58	81	1	0.0009771754
rs2836527	38869617	63	78	1	0.0009883290
rs2833309	31449008	71	36	1	0.0021036509
rs1492958	16564622	77	90	1	0.0008683172
rs2828670	24290625	79	33	1	0.0022362644
rs17768485	43547198	89	96	1	0.0008463041
rs2823980	16984585	93	95	1	0.0008542645
rs7279742	42662505	99	29	1	0.0026978579

Table 2.2: Bipolar disorder, Chromosome 21. Single-SNP and hybrid approaches.

SNP identifier	Location	single-SNP; rank	hybrid; rank	single-SNP; p	hybrid; vi
rs16997735	39792304	1	3	0	0.0407872915
rs2837588	40669929	2	57	0.002	0.0015214547
rs7276641	25799569	3	10	0.019	0.0106955958
rs13340018	31477667	4	21	0.201	0.0048628810
rs980184	39978208	8	38	0.499	0.0021083418
rs999789	39348321	12	4	0.616	0.0292488819
rs9980699	34421519	16	40	0.725	0.0020450120
rs12482209	46488703	24	98	0.996	0.0010388596
rs724070	33353710	40	65	1	0.0013548513
rs455304	40886245	46	15	1	0.0062447878
rs2834630	35048509	47	85	1	0.0011372774
rs2836525	38867719	52	91	1	0.0011127211
rs997724	24057640	65	75	1	0.0012331953
rs2828835	24407865	87	51	1	0.0016604209
rs2823980	16984585	93	88	1	0.0011260758

Table 2.3: Bipolar disorder, Chromosome 21. T-Trees and hybrid approaches.

SNP identifier	Location	T-Trees; rank	hybrid; rank	T-Trees; vi	hybrid; vi
rs16997735	39792304	1	3	0.0376263891	0.0407872915
rs8130402	39342279	2	1	0.0360838641	0.0733214590
rs999789	39348321	3	4	0.0239925579	0.0292488819
rs2837588	40669929	4	57	0.0130988202	0.0015214547
rs7276641	25799569	5	10	0.0121321338	0.0106955958
rs2837630	40740413	7	7	0.0106342742	0.0134262564
rs428424	39367189	8	35	0.0089987243	0.0023067724
rs9974286	37378702	9	9	0.0086580713	0.0107358640
rs2838864	45535902	10	8	0.0082222497	0.0113158681
rs13340018	31477667	11	21	0.0080232522	0.0048628810
rs999790	39348638	13	50	0.0063667008	0.0017111696
rs4816560	37409431	15	12	0.0050165562	0.0073868189
rs2834630	35048509	16	85	0.0049274373	0.0011372774
rs2837632	40741151	18	13	0.0044681310	0.0072270412
rs4817844	37368934	19	27	0.0043857154	0.0031985464
rs724070	33353710	21	65	0.0043161571	0.0013548513
rs7275582	37373000	22	11	0.0041952677	0.0083723553
rs2255774	45508279	23	17	0.0037829855	0.0057648000
rs9975007	24254669	24	89	0.0036704783	0.0011248410
rs464509	26510706	25	14	0.0034873267	0.0071886567
rs2827836	23380238	26	16	0.0032807053	0.0058030761
rs2007261	31450121	28	66	0.0027487143	0.0013388016
rs455304	40886245	30	15	0.0026384978	0.0062447878
rs1893382	16310882	31	5	0.0025766323	0.0159368056
rs2837654	40776531	32	22	0.0022860666	0.0042815415
rs8126930	45496143	35	29	0.0021283241	0.0030126037
rs9980699	34421519	38	40	0.0020291073	0.0020450120
rs9975168	37372427	43	80	0.0016990174	0.0012040487
rs467491	26519761	49	23	0.0015393797	0.0034570282
rs2828801	24377703	51	30	0.0015133328	0.0029320817
rs461094	40888729	53	32	0.0014654228	0.0026709509
rs2300390	34891385	56	20	0.0014073335	0.0050305003
rs2823416	15944080	57	24	0.0013442458	0.0034256866
rs980184	39978208	58	38	0.0012963971	0.0021083418
rs7279935	16304787	60	6	0.0012704028	0.0135584535
rs2409950	38330845	64	34	0.0011506354	0.0024932470
rs181146	27726545	65	46	0.0011495699	0.0017748185
rs463481	40888309	68	26	0.0010837656	0.0033210437
SNP A-2019458	39887600	71	68	0.0010612114	0.0013060428
rs459139	40887696	76	64	0.0010346442	0.0013971396
rs1847893	23381454	85	25	0.0009298437	0.0033869267
rs2823980	16984585	95	88	0.0008542645	0.0011260758
rs17241907	17099851	98	33	0.0008393119	0.0025304831

Table 2.4: Bipolar disorder, Chromosome 21. Single-SNP, T-Trees and hybrid approaches.

SNP identifier	Location	single-SNP; rank	T-Trees; rank	hybrid; rank	single-SNP; p	T-Trees; vi	hybrid; vi
rs16997735	39792304	1	1	3	0	0.0376263891	0.0407872915
rs2837588	40669929	2	4	57	0.002	0.0130988202	0.0015214547
rs7276641	25799569	3	5	10	0.019	0.0121321338	0.0106955958
rs13340018	31477667	4	11	21	0.201	0.0080232522	0.0048628810
rs980184	39978208	8	58	38	0.499	0.0012963971	0.0021083418
rs999789	39348321	12	3	4	0.616	0.0239925579	0.0292488819
rs9980699	34421519	16	38	40	0.725	0.0020291073	0.0020450120
rs724070	33353710	40	21	65	1	0.0043161571	0.0013548513
rs455304	40886245	46	30	15	1	0.0026384978	0.0062447878
rs2834630	35048509	47	16	85	1	0.0049274373	0.0011372774
rs2823980	16984585	93	95	88	1	0.0008542645	0.0011260758

3 Coronary artery disease, Chromosome 05

Table 3.1: Coronary artery disease, Chromosome 05. Single-SNP and T-Trees approaches.

SNP identifier	Location	single-SNP; rank	T-Trees; rank	single-SNP; p	T-Trees; vi
rs2416472	117033845	1	4	0	0.0497860206
rs17076079	172898758	1	1	0	0.2571704213
rs159171	21222083	1	3	0	0.1439776507
rs17136296	113485659	4	6	0	0.0209207111
rs6451780	44896214	5	85	0.003	0.0002783858
rs17068248	166645337	6	56	0.033	0.0004042143
SNP A-1982751	103802011	7	18	0.065	0.0032242807
rs16884665	10465682	18	72	0.748	0.0003168655
rs325857	41080373	23	82	0.92	0.0002885458
rs325870	41087696	24	77	0.926	0.0003034803
rs11135206	161776793	28	35	0.968	0.0010715547

Table 3.2: Coronary artery disease, Chromosome 05. Single-SNP and hybrid approaches.

SNP identifier	Location	single-SNP; rank	hybrid; rank	single-SNP; p	hybrid; vi
rs2416472	117033845	1	4	0	0.0589159163
rs17076079	172898758	1	1	0	0.2619770457
rs159171	21222083	1	2	0	0.1465241779
rs17136296	113485659	4	6	0	0.0200712296
rs6451780	44896214	5	18	0.003	0.0022685687
rs17068248	166645337	6	8	0.033	0.0049100173
SNP A-1982751	103802011	7	10	0.065	0.0045502730
rs2562544	36560514	12	64	0.365	0.0005052855
rs10076949	158592088	13	26	0.597	0.0014420404
rs16884665	10465682	18	33	0.748	0.0010517332
rs2964748	6280738	19	69	0.806	0.0004657849
rs16896571	27291525	22	45	0.917	0.0008107014
rs325857	41080373	23	44	0.92	0.0008485956
rs325870	41087696	24	37	0.926	0.0009675325
rs11135206	161776793	28	76	0.968	0.0004457188
rs2560413	5202769	35	56	0.995	0.0005814892
rs10065792	133065990	71	98	1	0.0003199621
rs7720552	127668844	91	81	1	0.0004035628
rs730925	12030349	99	67	1	0.0004784561

Table 3.3: Coronary artery disease, Chromosome 05. T-Trees and hybrid approaches.

SNP identifier	Location	T-Trees; rank	hybrid; rank	T-Trees; vi	hybrid; vi
rs17076079	172898758	1	1	0.2571704213	0.2619770457
rs17411921	117033876	2	3	0.1713919542	0.1340952991
rs159171	21222083	3	2	0.1439776507	0.1465241779
rs2416472	117033845	4	4	0.0497860206	0.0589159163
rs17136296	113485659	6	6	0.0209207111	0.0200712296
rs17403069	116972569	7	75	0.0166133546	0.0004497057
rs17142889	117068908	9	7	0.0072115821	0.0065536082
rs7714451	117037818	10	17	0.0056504113	0.0026636061
rs2131077	21217240	11	32	0.0054719628	0.0010855870
rs16888991	21204115	13	80	0.0041733192	0.0004107480
rs6869179	21231261	14	19	0.0040661830	0.0020824585
rs17346822	117058221	15	9	0.0040250003	0.0046028121
rs6421871	117068096	16	14	0.0037602052	0.0033879408
SNP A-1982751	103802011	18	10	0.0032242807	0.0045502730
rs4957159	41584367	20	15	0.0023877258	0.0033093224
rs9327047	116999827	21	11	0.0023803401	0.0041142653
rs9327048	117001096	24	12	0.0019670936	0.0040160744
rs822129	41581699	25	22	0.0019547289	0.0018241595
rs17346461	117042900	29	27	0.0012398525	0.0014334656
rs17052031	172901715	33	52	0.0011589024	0.0006604124
rs11135206	161776793	35	76	0.0010715547	0.0004457188
rs397925	116998548	36	47	0.0009280390	0.0007697121
rs822135	41590915	38	24	0.0007923320	0.0016373165
rs367474	117004156	39	39	0.0006156918	0.0008951565
rs2935260	54480637	40	49	0.0005806864	0.0007519003
rs715891	145966276	41	36	0.0005638067	0.0009769466
rs17068248	166645337	56	8	0.0004042143	0.0049100173
rs417769	71847234	59	38	0.0003898391	0.0009588668
rs16884665	10465682	72	33	0.0003168655	0.0010517332
rs1526882	117054771	74	53	0.0003125759	0.0006265763
rs325870	41087696	77	37	0.0003034803	0.0009675325
rs2992406	54480740	79	79	0.0003003675	0.0004251708
rs325857	41080373	82	44	0.0002885458	0.0008485956
rs6451780	44896214	85	18	0.0002783858	0.0022685687
rs7731936	157077770	87	30	0.0002755552	0.0012722295
rs322486	145989011	93	59	0.0002675635	0.0005489849

Table 3.4: Coronary artery disease, Chromosome 05. Single-SNP, T-Trees and hybrid approaches.

SNP identifier	Location	single-SNP; rank	T-Trees; rank	hybrid; rank	single-SNP; p	T-Trees; vi	hybrid; vi
rs2416472	117033845	1	4	4	0	0.0497860206	0.0589159163
rs17076079	172898758	1	1	1	0	0.2571704213	0.2619770457
rs159171	21222083	1	3	2	0	0.1439776507	0.1465241779
rs17136296	113485659	4	6	6	0	0.0209207111	0.0200712296
rs6451780	44896214	5	85	18	0.003	0.0002783858	0.0022685687
rs17068248	166645337	6	56	8	0.033	0.0004042143	0.0049100173
SNP A-1982751	103802011	7	18	10	0.065	0.0032242807	0.0045502730
rs16884665	10465682	18	72	33	0.748	0.0003168655	0.0010517332
rs325857	41080373	23	82	44	0.92	0.0002885458	0.0008485956
rs325870	41087696	24	77	37	0.926	0.0003034803	0.0009675325
rs11135206	161776793	28	35	76	0.968	0.0010715547	0.0004457188

4 Coronary artery disease, Chromosome 06

Table 4.1: Coronary artery disease, Chromosome 06. Single-SNP and T-Trees approaches.

SNP identifier	Location	single-SNP; rank	T-Trees; rank	single-SNP; p	T-Trees; vi
rs9478945	150205883	1	1	0	0.0396467499
rs519758	13522579	2	5	0	0.0110432778
rs17055974	96603273	3	4	0	0.0160990372
rs16870039	18188344	4	3	0.019	0.0245871598
rs1738358	49361481	5	14	0.037	0.0053064470
rs6907487	151294692	7	77	0.221	0.0008220107
SNP A-1987732	133950890	8	18	0.221	0.0037822355
rs7775358	91726062	9	7	0.273	0.0090638963
rs9372190	108996515	10	44	0.322	0.0014008532
SNP A-1910247	138615031	13	65	0.67	0.0009570504
rs10901001	7044907	14	17	0.729	0.0040971056
rs1488304	94649676	17	33	0.771	0.0020878102
rs12198351	102622143	19	93	0.908	0.0007064078
rs1474787	151295075	20	68	0.944	0.0009195968
rs6924328	127076726	21	37	0.957	0.0017783165
rs4398751	156779713	22	27	0.974	0.0023841409
rs1441891	165576222	24	94	0.987	0.0007055782
rs217540	108595042	33	47	1	0.0013161791
rs7760006	161642047	35	20	1	0.0033597996
rs17061307	101572337	36	23	1	0.0026369873
rs4510651	163027087	39	43	1	0.0014073220
rs12529488	106791146	40	99	1	0.0006855112
rs6924456	49624032	49	29	1	0.0022640553
rs9491940	99217375	49	75	1	0.0008422041
rs10484758	127105239	53	61	1	0.0010082295
rs8191855	160405050	55	51	1	0.0012556072
rs6927367	35539817	71	39	1	0.0015650691

Table 4.2: Coronary artery disease, Chromosome 06. Single-SNP and hybrid approaches.

SNP identifier	Location	single-SNP; rank	hybrid; rank	single-SNP; p	hybrid; vi
rs9478945	150205883	1	2	0	0.0426715157
rs519758	13522579	2	4	0	0.0134622735
rs17055974	96603273	3	7	0	0.0116332061
rs16870039	18188344	4	19	0.019	0.0041331297
rs1738358	49361481	5	3	0.037	0.0140398685
rs6922269	151294678	6	80	0.191	0.0011007743
SNP A-1987732	133950890	8	8	0.221	0.0103470304
rs7775358	91726062	9	6	0.273	0.0118215472
rs9372190	108996515	10	23	0.322	0.0036907444
rs9404338	96721113	11	53	0.338	0.0017050688
rs943081	43988645	12	93	0.662	0.0009428713
SNP A-1910247	138615031	13	79	0.67	0.0011065328
rs10901001	7044907	14	59	0.729	0.0014511133
rs654940	168831666	15	99	0.739	0.0008421649
rs1488304	94649676	17	50	0.771	0.0018044206
rs1474787	151295075	20	49	0.944	0.0018602245
rs6924328	127076726	21	46	0.957	0.0019619287
rs4398751	156779713	22	37	0.974	0.0023680818
rs9295585	23455618	23	96	0.981	0.0008702140
rs1441891	165576222	24	57	0.987	0.0015049742
rs217540	108595042	33	97	1	0.0008545309
rs12529488	106791146	40	73	1	0.0011745208
rs4714888	45993039	48	72	1	0.0011855643
rs6924456	49624032	49	83	1	0.0010637405
rs9468355	28449856	74	69	1	0.0012144063
rs16875837	85953512	95	65	1	0.0012856561
rs1776447	37710438	100	91	1	0.0009509587

Table 4.3: Coronary artery disease, Chromosome 06. T-Trees and hybrid approaches.

SNP identifier	Location	T-Trees; rank	hybrid; rank	T-Trees; vi	hybrid; vi
rs9478945	150205883	1	2	0.0396467499	0.0426715157
rs16870039	18188344	3	19	0.0245871598	0.0041331297
rs17055974	96603273	4	7	0.0160990372	0.0116332061
rs519758	13522579	5	4	0.0110432778	0.0134622735
rs6903505	91728665	6	9	0.0108839378	0.0083915359
rs7775358	91726062	7	6	0.0090638963	0.0118215472
rs6903322	84225687	8	13	0.0089829801	0.0061406168
rs10456820	18180845	9	24	0.0070987549	0.0036856639
rs945238	84222575	10	14	0.0061181572	0.0059751012
rs10943933	84228649	11	21	0.0054517437	0.0039902236
rs1738358	49361481	14	3	0.0053064470	0.0140398685
rs6454833	91748167	15	22	0.0045726327	0.0038057214
rs4708236	76999407	16	10	0.0042804011	0.0082723127
rs10901001	7044907	17	59	0.0040971056	0.0014511133
SNP A-1987732	133950890	18	8	0.0037822355	0.0103470304
rs7745251	84228475	21	32	0.0033579382	0.0028164179
rs9489754	98342750	22	12	0.0031785517	0.0064277741
rs2846530	162959035	24	20	0.0025890895	0.0041199966
rs9377535	104161850	26	15	0.0024192795	0.0050773517
rs4398751	156779713	27	37	0.0023841409	0.0023680818
rs1220445	91712833	28	43	0.0022717883	0.0020618240
rs6924456	49624032	29	83	0.0022640553	0.0010637405
rs9384677	108970125	31	31	0.0021448022	0.0028493994
rs7752426	165092140	32	25	0.0021059080	0.0035535597
rs1488304	94649676	33	50	0.0020878102	0.0018044206
rs9384678	108970234	36	27	0.0018172206	0.0032737769
rs6924328	127076726	37	46	0.0017783165	0.0019619287
rs1934811	148513254	38	34	0.0017381262	0.0026229711
rs9372190	108996515	44	23	0.0014008532	0.0036907444
rs2846515	162973728	46	51	0.0013437255	0.0017780613
rs217540	108595042	47	97	0.0013161791	0.0008545309
rs2803047	162959760	49	90	0.0012681101	0.0009637259
rs11752531	169163045	50	5	0.0012666092	0.0119444698
rs9481914	98320341	52	29	0.0012525041	0.0028653114
rs9377114	148514719	57	76	0.0010972942	0.0011675776
rs549419	49326307	64	30	0.0009846154	0.0028647165
SNP A-1910247	138615031	65	79	0.0009570504	0.0011065328
rs1474787	151295075	68	49	0.0009195968	0.0018602245
rs2883881	109004079	71	56	0.0008822374	0.0015067492
rs4260778	104178825	81	89	0.0007822112	0.0009748118
rs1441891	165576222	94	57	0.0007055782	0.0015049742
rs12529488	106791146	99	73	0.0006855112	0.0011745208

Table 4.4: Coronary artery disease, Chromosome 06. Single-SNP, T-Trees and hybrid approaches.

SNP identifier	Location	single-SNP; rank	T-Trees; rank	hybrid; rank	single-SNP; p	T-Trees; vi	hybrid; vi
rs9478945	150205883	1	1	2	0	0.0396467499	0.0426715157
rs519758	13522579	2	5	4	0	0.0110432778	0.0134622735
rs17055974	96603273	3	4	7	0	0.0160990372	0.0116332061
rs16870039	18188344	4	3	19	0.019	0.0245871598	0.0041331297
rs1738358	49361481	5	14	3	0.037	0.0053064470	0.0140398685
SNP A-1987732	133950890	8	18	8	0.221	0.0037822355	0.0103470304
rs7775358	91726062	9	7	6	0.273	0.0090638963	0.0118215472
rs9372190	108996515	10	44	23	0.322	0.0014008532	0.0036907444
SNP A-1910247	138615031	13	65	79	0.67	0.0009570504	0.0011065328
rs10901001	7044907	14	17	59	0.729	0.0040971056	0.0014511133
rs1488304	94649676	17	33	50	0.771	0.0020878102	0.0018044206
rs1474787	151295075	20	68	49	0.944	0.0009195968	0.0018602245
rs6924328	127076726	21	37	46	0.957	0.0017783165	0.0019619287
rs4398751	156779713	22	27	37	0.974	0.0023841409	0.0023680818
rs1441891	165576222	24	94	57	0.987	0.0007055782	0.0015049742
rs217540	108595042	33	47	97	1	0.0013161791	0.0008545309
rs12529488	106791146	40	99	73	1	0.0006855112	0.0011745208
rs6924456	49624032	49	29	83	1	0.0022640553	0.0010637405

5 Crohn's disease, Chromosome 01

Table 5.1: Crohn's disease, Chromosome 01. Single-SNP and T-Trees approaches.

SNP identifier	Location	single-SNP; rank	T-Trees; rank	single-SNP; p	T-Trees; vi
rs12078461	117273572	1	2	0	0.1491234057
rs1933641	216535584	1	1	0	0.2227965124
rs11209026	67478546	1	5	0	0.0303647416
rs10489629	67460937	5	22	0	0.0022728981
rs2201841	67466790	6	20	0	0.0025781386
rs12119179	67520003	7	35	0	0.0011869244
rs11209033	67517088	8	74	0	0.0005163786
rs17096872	58745260	9	8	0	0.0155397910
rs17130103	87923130	10	6	0	0.0212685584
rs7546245	67523062	11	54	0	0.0006809202
SNP A-2045577	67462196	12	48	0	0.0007778134
rs12141431	67519611	18	99	0.001	0.0004104127
rs17101358	78377669	19	15	0.002	0.0047450549
rs6660226	67517189	21	65	0.003	0.0005650935
rs6677092	238239905	23	11	0.017	0.0115811245
rs16850854	201223211	24	60	0.038	0.0005997367
SNP A-2111030	65186205	25	24	0.057	0.0021274066
rs2492958	28187010	26	83	0.179	0.0004693104
rs686190	12146426	31	29	0.29	0.0017360547
rs16847490	225488622	34	28	0.517	0.0018723262
rs17116805	58303794	37	70	0.626	0.0005289743
rs12409315	3219118	38	34	0.65	0.0011978668
rs13375635	219294692	56	67	0.972	0.0005583512
rs790636	67461021	98	18	1	0.0038877315

Table 5.2: Crohn's disease, Chromosome 01. Single-SNP and hybrid approaches.

SNP identifier	Location	single-SNP; rank	hybrid; rank	single-SNP; p	hybrid; vi
rs12078461	117273572	1	2	0	0.1457913381
rs1933641	216535584	1	1	0	0.2276438256
rs11209026	67478546	1	5	0	0.0300032480
rs11805303	67448104	4	41	0	0.0012314785
rs10489629	67460937	5	19	0	0.0052229238
rs2201841	67466790	6	40	0	0.0013295015
rs12119179	67520003	7	65	0	0.0004862244
rs17096872	58745260	9	7	0	0.0168155793
rs17130103	87923130	10	6	0	0.0223331608
SNP A-2045577	67462196	12	35	0	0.0016893976
rs17375018	67427735	13	59	0.001	0.0005387476
rs7515029	67368960	14	31	0.001	0.0020198491
rs4655679	67372245	16	96	0.001	0.0003283221
rs17101358	78377669	19	14	0.002	0.0071728527
rs6677092	238239905	23	11	0.017	0.0097547179
rs16850854	201223211	24	22	0.038	0.0036465419
SNP A-2111030	65186205	25	25	0.057	0.0034337091
rs2492958	28187010	26	34	0.179	0.0017411612
rs2484158	180256299	28	57	0.237	0.0005449385
rs686190	12146426	31	29	0.29	0.0020468331
rs16847490	225488622	34	36	0.517	0.0015801174
rs17672135	238512219	36	60	0.612	0.0005367955
rs17116805	58303794	37	52	0.626	0.0006797639
rs12409315	3219118	38	63	0.65	0.0004986035
rs10916631	222774675	48	83	0.852	0.0003983281
rs13375635	219294692	56	47	0.972	0.0008874373
rs17104225	48403527	66	90	0.985	0.0003721541
rs2253677	154437920	74	89	0.999	0.0003793783
rs4633297	104600152	78	48	0.999	0.0008780895
SNP A-1827111	77289597	94	21	1	0.0037285205

Table 5.3: Crohn's disease, Chromosome 01. T-Trees and hybrid approaches.

SNP identifier	Location	T-Trees; rank	hybrid; rank	T-Trees; vi	hybrid; vi
rs1933641	216535584	1	1	0.2227965124	0.2276438256
rs12078461	117273572	2	2	0.1491234057	0.1457913381
rs17036573	117272017	3	3	0.0503877425	0.0620549160
rs2806863	117271036	4	10	0.0312668956	0.0110092271
rs11209026	67478546	5	5	0.0303647416	0.0300032480
rs17130103	87923130	6	6	0.0212685584	0.0223331608
rs10802825	238243223	7	9	0.0178639581	0.0130229282
rs17096872	58745260	8	7	0.0155397910	0.0168155793
rs2254224	117259387	9	8	0.0143032462	0.0142332308
rs2806864	117271304	10	12	0.0126146472	0.0091589215
rs6677092	238239905	11	11	0.0115811245	0.0097547179
rs2250457	117267288	12	23	0.0049904738	0.0035304938
rs943371	117269213	13	17	0.0048972723	0.0060078891
rs17101358	78377669	15	14	0.0047450549	0.0071728527
rs2201841	67466790	20	40	0.0025781386	0.0013295015
rs10489629	67460937	22	19	0.0022728981	0.0052229238
rs2066300	238250409	23	26	0.0021418905	0.0025088334
SNP A-2111030	65186205	24	25	0.0021274066	0.0034337091
rs16847490	225488622	28	36	0.0018723262	0.0015801174
rs686190	12146426	29	29	0.0017360547	0.0020468331
rs6658302	77681573	30	74	0.0015682077	0.0004317437
rs1782127	90052930	32	15	0.0015229747	0.0064897502
rs12409315	3219118	34	63	0.0011978668	0.0004986035
rs12119179	67520003	35	65	0.0011869244	0.0004862244
rs909812	22475953	39	18	0.0011570421	0.0053914955
rs2250865	117264214	41	58	0.0010463773	0.0005413664
rs2806880	117280768	42	38	0.0010402890	0.0013923318
rs3917781	167836357	43	46	0.0010128916	0.0009049639
rs3768539	156327197	45	78	0.0008527195	0.0004278692
rs2251406	117246888	46	42	0.0008134451	0.0010183323
rs6674713	11473219	47	100	0.0008036643	0.0003180336
SNP A-2045577	67462196	48	35	0.0007778134	0.0016893976
rs4654792	22473259	51	20	0.0007218611	0.0040131784
rs6427106	165980140	55	99	0.0006590229	0.0003233416
rs10489628	67476695	57	24	0.0006518679	0.0034694361
rs16850854	201223211	60	22	0.0005997367	0.0036465419
rs2319403	29493343	63	33	0.0005747603	0.0017796365
rs1960306	88665551	64	85	0.0005693630	0.0003866812
rs13375635	219294692	67	47	0.0005583512	0.0008874373
rs17116805	58303794	70	52	0.0005289743	0.0006797639
rs2492958	28187010	83	34	0.0004693104	0.0017411612
rs7518159	29494047	90	30	0.0004465078	0.0020364094

Table 5.4: Crohn's disease, Chromosome 01. Single-SNP, T-Trees and hybrid approaches.

SNP identifier	Location	single-SNP; rank	T-Trees; rank	hybrid; rank	single-SNP; p	T-Trees; vi	hybrid; vi
rs12078461	117273572	1	2	2	0	0.1491234057	0.1457913381
rs1933641	216535584	1	1	1	0	0.2227965124	0.2276438256
rs11209026	67478546	1	5	5	0	0.0303647416	0.0300032480
rs10489629	67460937	5	22	19	0	0.0022728981	0.0052229238
rs2201841	67466790	6	20	40	0	0.0025781386	0.0013295015
rs12119179	67520003	7	35	65	0	0.0011869244	0.0004862244
rs17096872	58745260	9	8	7	0	0.0155397910	0.0168155793
rs17130103	87923130	10	6	6	0	0.0212685584	0.0223331608
SNP A-2045577	67462196	12	48	35	0	0.0007778134	0.0016893976
rs17101358	78377669	19	15	14	0.002	0.0047450549	0.0071728527
rs6677092	238239905	23	11	11	0.017	0.0115811245	0.0097547179
rs16850854	201223211	24	60	22	0.038	0.0005997367	0.0036465419
SNP A-2111030	65186205	25	24	25	0.057	0.0021274066	0.0034337091
rs2492958	28187010	26	83	34	0.179	0.0004693104	0.0017411612
rs686190	12146426	31	29	29	0.29	0.0017360547	0.0020468331
rs16847490	225488622	34	28	36	0.517	0.0018723262	0.0015801174
rs17116805	58303794	37	70	52	0.626	0.0005289743	0.0006797639
rs12409315	3219118	38	34	63	0.65	0.0011978668	0.0004986035
rs13375635	219294692	56	67	47	0.972	0.0005583512	0.0008874373

6 Crohn's disease, Chromosome 20

Table 6.1: Crohn's disease, Chromosome 20. Single-SNP and T-Trees approaches.

SNP identifier	Location	single-SNP; rank	T-Trees; rank	single-SNP; p	T-Trees; vi
rs6044514	16856038	1	1	0	0.0296018421
rs6125996	35788083	2	3	0.15	0.0092730573
rs6040103	10642742	4	2	0.462	0.0104239022
rs2424422	21916219	10	9	0.854	0.0031795414
rs16996531	9820603	12	10	0.912	0.0031713037
rs6012081	44992741	15	5	0.94	0.0065346687
rs13037694	13508308	19	34	0.994	0.0012633897
rs2207719	7040386	21	64	0.996	0.0008112721
rs6018339	45265004	24	22	1	0.0018285604
rs6031477	42315623	26	15	1	0.0023139428
rs6027005	57632613	29	16	1	0.0022826019
rs142092	7041432	30	71	1	0.0007718263
rs6011600	61183435	31	14	1	0.0023566532
rs16981111	19708553	34	30	1	0.0014034738
rs3026550	57345571	39	80	1	0.0007284617
rs12480690	19703487	40	29	1	0.0014082033
rs16993927	46625798	42	78	1	0.0007337512
rs16996688	9976725	43	12	1	0.0027739249
rs4246446	60569315	46	68	1	0.0007959241
rs2426052	46000175	50	17	1	0.0021083053
rs746219	57832814	58	8	1	0.0032709235
rs16992637	6762338	60	61	1	0.0008335761
rs6047596	2173481	66	11	1	0.0029700860
rs6118724	9694345	67	76	1	0.0007382174
rs17092750	33578374	68	66	1	0.0008036584
rs2865972	59058343	71	54	1	0.0009005044
rs2143607	42272126	82	47	1	0.0010197415
rs6086197	7705151	84	25	1	0.0016527316
rs2425860	44360284	89	99	1	0.0006573369
rs6127097	52157302	91	19	1	0.0019335780
rs844912	23088535	100	59	1	0.0008496331

Table 6.2: Crohn's disease, Chromosome 20. Single-SNP and hybrid approaches.

SNP identifier	Location	single-SNP; rank	hybrid; rank	single-SNP; p	hybrid; vi
rs6044514	16856038	1	2	0	0.0452794977
rs6125996	35788083	2	3	0.15	0.0174648770
rs6040103	10642742	4	5	0.462	0.0054486522
rs16992325	6432249	9	4	0.835	0.0076027652
rs2424422	21916219	10	20	0.854	0.0020684729
rs16996531	9820603	12	26	0.912	0.0018229790
rs6012081	44992741	15	8	0.94	0.0037916117
rs13037694	13508308	19	18	0.994	0.0021418804
rs2207719	7040386	21	71	0.996	0.0010382959
rs2206437	37426155	22	19	0.999	0.0021188581
rs6031477	42315623	26	10	1	0.0032512736
rs932385	37427596	27	64	1	0.0011040771
rs142092	7041432	30	45	1	0.0013375214
rs6011600	61183435	31	34	1	0.0014966467
rs16981111	19708553	34	50	1	0.0012974103
rs12480690	19703487	40	81	1	0.0009364693
rs16996688	9976725	43	76	1	0.0009822003
rs882807	805132	57	59	1	0.0011400386
rs6047596	2173481	66	40	1	0.0013643799
rs1701858	37705857	80	36	1	0.0014580212
rs6134907	13477662	83	87	1	0.0008881029
rs6086197	7705151	84	24	1	0.0019267975
rs6127097	52157302	91	37	1	0.0014499697
rs1160080	37705504	95	48	1	0.0013100585
rs844912	23088535	100	84	1	0.0008966419

Table 6.3: Crohn's disease, Chromosome 20. T-Trees and hybrid approaches.

SNP identifier	Location	T-Trees; rank	hybrid; rank	T-Trees; vi	hybrid; vi
rs6044514	16856038	1	2	0.0296018421	0.0452794977
rs6040103	10642742	2	5	0.0104239022	0.0054486522
rs6125996	35788083	3	3	0.0092730573	0.0174648770
rs6012081	44992741	5	8	0.0065346687	0.0037916117
rs910118	10604087	7	33	0.0033663924	0.0014981539
rs2424422	21916219	9	20	0.0031795414	0.0020684729
rs16996531	9820603	10	26	0.0031713037	0.0018229790
rs6047596	2173481	11	40	0.0029700860	0.0013643799
rs16996688	9976725	12	76	0.0027739249	0.0009822003
rs6011600	61183435	14	34	0.0023566532	0.0014966467
rs6031477	42315623	15	10	0.0023139428	0.0032512736
rs6027185	57908702	18	90	0.0019706803	0.0008708654
rs6127097	52157302	19	37	0.0019335780	0.0014499697
rs6086197	7705151	25	24	0.0016527316	0.0019267975
rs12480690	19703487	29	81	0.0014082033	0.0009364693
rs16981111	19708553	30	50	0.0014034738	0.0012974103
rs13037694	13508308	34	18	0.0012633897	0.0021418804
rs1555322	33312595	39	6	0.0011505576	0.0043098777
rs6053199	5131713	44	21	0.0010720996	0.0020347480
rs844912	23088535	59	84	0.0008496331	0.0008966419
rs2144933	5379173	60	14	0.0008417880	0.0024034690
rs2207719	7040386	64	71	0.0008112721	0.0010382959
rs142092	7041432	71	45	0.0007718263	0.0013375214
rs2425037	33319843	90	9	0.0006904516	0.0036876865
rs13045526	13543807	91	51	0.0006860640	0.0012854210

Table 6.4: Crohn's disease, Chromosome 20. Single-SNP, T-Trees and hybrid approaches.

SNP identifier	Location	single-SNP; rank	T-Trees; rank	hybrid; rank	single-SNP; p	T-Trees; vi	hybrid; vi
rs6044514	16856038	1	1	2	0	0.0296018421	0.0452794977
rs6125996	35788083	2	3	3	0.15	0.0092730573	0.0174648770
rs6040103	10642742	4	2	5	0.462	0.0104239022	0.0054486522
rs2424422	21916219	10	9	20	0.854	0.0031795414	0.0020684729
rs16996531	9820603	12	10	26	0.912	0.0031713037	0.0018229790
rs6012081	44992741	15	5	8	0.94	0.0065346687	0.0037916117
rs13037694	13508308	19	34	18	0.994	0.0012633897	0.0021418804
rs2207719	7040386	21	64	71	0.996	0.0008112721	0.0010382959
rs6031477	42315623	26	15	10	1	0.0023139428	0.0032512736
rs142092	7041432	30	71	45	1	0.0007718263	0.0013375214
rs6011600	61183435	31	14	34	1	0.0023566532	0.0014966467
rs16981111	19708553	34	30	50	1	0.0014034738	0.0012974103
rs12480690	19703487	40	29	81	1	0.0014082033	0.0009364693
rs16996688	9976725	43	12	76	1	0.0027739249	0.0009822003
rs6047596	2173481	66	11	40	1	0.0029700860	0.0013643799
rs6086197	7705151	84	25	24	1	0.0016527316	0.0019267975
rs6127097	52157302	91	19	37	1	0.0019335780	0.0014499697
rs844912	23088535	100	59	84	1	0.0008496331	0.0008966419

7 Hypertension, Chromosome 10

Table 7.1: Hypertension, Chromosome 10. Single-SNP and T-Trees approaches.

SNP identifier	Location	single-SNP; rank	T-Trees; rank	single-SNP; p	T-Trees; vi
rs11005510	58202995	1	1	0	0.0841503659
rs17157250	1913202	2	12	0.15	0.0047277850
rs11185734	91253844	3	47	0.231	0.0013029791
rs7905911	107754628	4	15	0.319	0.0032399618
rs1691907	29165925	27	29	0.943	0.0019557906
rs7081531	21355069	29	28	0.954	0.0020499962
rs7894018	92807259	33	43	0.984	0.0014401931
rs1617402	95157731	39	86	0.994	0.0007694068
rs2458705	2531377	41	23	0.996	0.0022880667
rs7901497	52313828	46	64	0.997	0.0009971235
rs6415963	21280349	48	93	0.999	0.0006914531
rs16922382	22829558	61	38	1	0.0015561471
rs10881889	92878888	96	21	1	0.0024471637

Table 7.2: Hypertension, Chromosome 10. Single-SNP and hybrid approaches.

SNP identifier	Location	single-SNP; rank	hybrid; rank	single-SNP; p	hybrid; vi
rs11005510	58202995	1	2	0	0.0865388191
rs17157250	1913202	2	19	0.15	0.0043408900
rs11185734	91253844	3	63	0.231	0.0013969302
rs7905911	107754628	4	22	0.319	0.0036558188
rs10904966	6870968	16	39	0.772	0.0020757080
rs1691907	29165925	27	20	0.943	0.0041493699
rs7081531	21355069	29	42	0.954	0.0018588073
rs7894018	92807259	33	7	0.984	0.0089155660
rs2458705	2531377	41	49	0.996	0.0017362040
rs6415963	21280349	48	88	0.999	0.0009673225
rs2895065	10320197	85	38	1	0.0021110533
rs10881889	92878888	96	21	1	0.0036700870

Table 7.3: Hypertension, Chromosome 10. T-Trees and hybrid approaches.

SNP identifier	Location	T-Trees; rank	hybrid; rank	T-Trees; vi	hybrid; vi
rs11005510	58202995	1	2	0.0841503659	0.0865388191
rs2393191	58394623	2	3	0.0161815827	0.0121433048
rs1608110	58285889	3	4	0.0117370524	0.0104891795
rs16909905	58410545	4	8	0.0098739411	0.0072998353
rs1915936	58202771	5	18	0.0091376341	0.0047335256
rs11591951	80221922	6	13	0.0069200738	0.0061658613
rs1566222	70189609	7	10	0.0058050432	0.0063961342
rs11005491	58177677	8	34	0.0055918598	0.0022238339
rs1955196	14820745	9	14	0.0054406325	0.0061321657
rs1925315	58261483	10	11	0.0052430820	0.0062704344
rs1162961	70167488	11	16	0.0048854590	0.0054694301
rs17157250	1913202	12	19	0.0047277850	0.0043408900
rs2601749	14821336	14	6	0.0034148606	0.0098171237
rs7905911	107754628	15	22	0.0032399618	0.0036558188
rs7084148	805221	17	12	0.0029898686	0.0061931007
rs2170862	118610986	18	27	0.0029322211	0.0031178725
rs10881889	92878888	21	21	0.0024471637	0.0036700870
rs2458705	2531377	23	49	0.0022880667	0.0017362040
rs9329290	2532751	25	47	0.0022607460	0.0017869770
rs2601751	14821179	26	48	0.0022348929	0.0017710699
rs7087685	118606527	27	26	0.0021476201	0.0032790753
rs7081531	21355069	28	42	0.0020499962	0.0018588073
rs1691907	29165925	29	20	0.0019557906	0.0041493699
rs10996895	67529571	30	17	0.0019200927	0.0049952242
rs7067899	92881044	31	30	0.0018820085	0.0026229898
rs2768703	14820322	32	32	0.0016971552	0.0023753657
rs1555989	14824092	33	36	0.0016963134	0.0021829716
rs11191996	106055721	34	25	0.0016463402	0.0033255506
rs11002673	80222159	35	29	0.0016219619	0.0028020266
rs1914595	58222299	40	50	0.0014817253	0.0016967738
rs7894018	92807259	43	7	0.0014401931	0.0089155660
rs11185734	91253844	47	63	0.0013029791	0.0013969302
rs1681752	118601393	52	44	0.0011999527	0.0018469495
rs7096109	67513478	55	40	0.0011629189	0.0018789548
rs2627197	118612118	56	100	0.0011449204	0.0008750361
rs11186914	94052611	57	57	0.0011363106	0.0015969476
rs1955195	14820794	59	55	0.0010754979	0.0016389621
rs1300253	70172294	61	59	0.0010165696	0.0014695011
rs11254798	6922666	62	79	0.0010016999	0.0010431666
rs1866305	65440899	63	54	0.0010003887	0.0016397429
rs11254836	6955867	65	24	0.0009964716	0.0034131172
rs2601750	14821242	66	60	0.0009804929	0.0014273035
rs2250544	84437807	67	68	0.0009617115	0.0012450397
rs17459521	80166482	68	15	0.0009380075	0.0056510359
rs10822723	67545462	71	64	0.0009067805	0.0013289473
rs1340137	16297748	72	28	0.0008803842	0.0028710142
rs2250844	65417479	77	69	0.0008301467	0.0012325760
rs7069728	808851	78	9	0.0008191812	0.0065504439
rs11254853	6967423	83	33	0.0007841912	0.0022369328
rs7075577	14821059	84	53	0.0007750793	0.0016557470
rs11254803	6932247	85	98	0.0007745813	0.0008860382
rs276207	106055454	92	31	0.0006950154	0.0024343721
rs6415963	21280349	93	88	0.0006914531	0.0009673225
rs11010228	35892021	95	56	0.0006684104	0.0016136887
rs11254838	6956687	97	87	0.0006504787	0.0009678617

Table 7.4: Hypertension, Chromosome 10. Single-SNP, T-Trees and hybrid approaches.

SNP identifier	Location	single-SNP; rank	T-Trees; rank	hybrid; rank	single-SNP; p	T-Trees; vi	hybrid; vi
rs11005510	58202995	1	1	2	0	0.0841503659	0.0865388191
rs17157250	1913202	2	12	19	0.15	0.0047277850	0.0043408900
rs11185734	91253844	3	47	63	0.231	0.0013029791	0.0013969302
rs7905911	107754628	4	15	22	0.319	0.0032399618	0.0036558188
rs1691907	29165925	27	29	20	0.943	0.0019557906	0.0041493699
rs7081531	21355069	29	28	42	0.954	0.0020499962	0.0018588073
rs7894018	92807259	33	43	7	0.984	0.0014401931	0.0089155660
rs2458705	2531377	41	23	49	0.996	0.0022880667	0.0017362040
rs6415963	21280349	48	93	88	0.999	0.0006914531	0.0009673225
rs10881889	92878888	96	21	21	1	0.0024471637	0.0036700870

8 Hypertension, Chromosome 14

Table 8.1: Hypertension, Chromosome 14. Single-SNP and T-Trees approaches.

SNP identifier	Location	single-SNP; rank	T-Trees; rank	single-SNP; p	T-Trees; vi
rs8011855	38673786	1	5	0	0.0298551353
rs1957779	62739400	2	7	0	0.0284964121
rs1457015	82704632	3	18	0.002	0.0092820857
rs6574988	87629745	4	9	0.004	0.0175690479
rs7142143	50473281	5	38	0.004	0.0028333437
rs2653550	78672810	6	29	0.026	0.0038620063
rs1253672	51459138	7	74	0.03	0.0007985511
SNP A-2203090	32214885	8	30	0.217	0.0037660919
rs6574590	80151227	9	61	0.443	0.0012010403
rs1278930	31486031	10	71	0.45	0.0009026637
rs17098915	61129684	11	41	0.486	0.0024481089
rs10483456	35105918	13	97	0.628	0.0006267321
rs4983425	105032574	18	81	0.842	0.0007446908
rs1958305	23342964	21	8	0.889	0.0187541270
rs1241649	24383933	22	68	0.916	0.0009770084
rs7145344	60766603	28	65	0.979	0.0011473412
rs435340	44004257	30	39	0.982	0.0027815511
rs7149343	67634464	35	40	0.998	0.0027254172
rs1957508	99403553	37	94	0.999	0.0006324696
rs7150537	92331535	52	54	1	0.0014284773
rs8016735	78732379	56	76	1	0.0007887189
rs2194622	44358898	62	63	1	0.0011662734
rs2400997	100796860	66	62	1	0.0011882915
rs17098327	31480919	67	42	1	0.0024214103
rs179350	44366743	86	91	1	0.0006550628

Table 8.2: Hypertension, Chromosome 14. Single-SNP and hybrid approaches.

SNP identifier	Location	single-SNP; rank	hybrid; rank	single-SNP; p	hybrid; vi
rs8011855	38673786	1	5	0	0.0406287873
rs1957779	62739400	2	13	0	0.0114005153
rs1457015	82704632	3	10	0.002	0.0129045232
rs6574988	87629745	4	8	0.004	0.0234725902
rs7142143	50473281	5	17	0.004	0.0097743195
rs2653550	78672810	6	14	0.026	0.0113375370
rs1253672	51459138	7	46	0.03	0.0018276704
SNP A-2203090	32214885	8	49	0.217	0.0016873485
rs6574590	80151227	9	44	0.443	0.0022137415
rs17098915	61129684	11	36	0.486	0.0038094737
rs10483456	35105918	13	29	0.628	0.0050535673
rs1958305	23342964	21	12	0.889	0.0122218227
rs1241649	24383933	22	40	0.916	0.0027470373
rs1189025	55941885	27	72	0.975	0.0010225101
rs435340	44004257	30	25	0.982	0.0062562922
rs7149343	67634464	35	32	0.998	0.0048198426
rs1245400	26713572	36	78	0.998	0.0009249570
rs7150537	92331535	52	38	1	0.0030667692
rs12590471	70070853	54	50	1	0.0016576418
rs2194622	44358898	62	97	1	0.0007270501
rs2400997	100796860	66	61	1	0.0013264366

Table 8.3: Hypertension, Chromosome 14. T-Trees and hybrid approaches.

SNP identifier	Location	T-Trees; rank	hybrid; rank	T-Trees; vi	hybrid; vi
rs7154773	59818871	1	2	0.0644925187	0.0582077909
rs10130695	59825737	2	22	0.0598629327	0.0074825819
rs4581640	87630108	3	11	0.0374141442	0.0124198735
rs8011227	59788029	4	6	0.0319095235	0.0331412378
rs8011855	38673786	5	5	0.0298551353	0.0406287873
rs1957779	62739400	7	13	0.0284964121	0.0114005153
rs1958305	23342964	8	12	0.0187541270	0.0122218227
rs6574988	87629745	9	8	0.0175690479	0.0234725902
rs2401743	87630502	10	3	0.0159588451	0.0581561843
rs17097262	59808110	11	24	0.0127828530	0.0062804165
rs7152965	62711170	12	100	0.0111512085	0.0007119917
rs7158657	59794810	15	26	0.0094915821	0.0061304217
rs11849674	59757776	16	28	0.0094767911	0.0051898687
rs2766696	99731073	17	9	0.0094259760	0.0191343532
rs1457015	82704632	18	10	0.0092820857	0.0129045232
rs8012816	59820118	19	15	0.0078603082	0.0107840397
rs8019531	59777164	20	4	0.0076175229	0.0434427656
rs10137732	59758962	22	19	0.0066940646	0.0087355265
rs6573298	59775222	23	23	0.0055413322	0.0068916880
rs2757528	99730830	25	21	0.0044831283	0.0081466932
rs188620	59758931	26	39	0.0041457529	0.0028056805
rs2653550	78672810	29	14	0.0038620063	0.0113375370
SNP A-2203090	32214885	30	49	0.0037660919	0.0016873485
rs12100601	23354013	32	7	0.0035134048	0.0263925920
rs10148587	59758913	36	34	0.0029708727	0.0044730268
rs2757527	99730686	37	18	0.0029456762	0.0091267080
rs7142143	50473281	38	17	0.0028333437	0.0097743195
rs435340	44004257	39	25	0.0027815511	0.0062562922
rs7149343	67634464	40	32	0.0027254172	0.0048198426
rs17098915	61129684	41	36	0.0024481089	0.0038094737
rs10142834	59804716	43	16	0.0021529844	0.0098938015
rs7154776	65614033	44	33	0.0021373241	0.0047158598
rs7147522	67646678	46	41	0.0018109249	0.0027072447
rs381820	44004352	47	37	0.0017307596	0.0031688352
rs7145505	59775328	48	56	0.0016618106	0.0014697335
rs986201	65606667	50	31	0.0016442436	0.0049919470
rs1887103	59812972	52	30	0.0015834835	0.0050215569
rs7150537	92331535	54	38	0.0014284773	0.0030667692
rs8008153	100809433	56	66	0.0012937491	0.0011721393
rs6574590	80151227	61	44	0.0012010403	0.0022137415
rs2400997	100796860	62	61	0.0011882915	0.0013264366
rs2194622	44358898	63	97	0.0011662734	0.0007270501
rs7157695	99966553	67	84	0.0010201699	0.0008718423
rs1241649	24383933	68	40	0.0009770084	0.0027470373
rs6573648	65617378	72	71	0.0008441621	0.0010807821
rs1253672	51459138	74	46	0.0007985511	0.0018276704
rs7156125	57200616	82	54	0.0007389873	0.0015019369
rs1189138	55955932	85	74	0.0006940654	0.0010146048
rs11628587	59777184	92	64	0.0006533327	0.0012385882
rs1341002	94432505	95	65	0.0006310478	0.0011735194
rs10483456	35105918	97	29	0.0006267321	0.0050535673

Table 8.4: Hypertension, Chromosome 14. Single-SNP, T-Trees and hybrid approaches.

SNP identifier	Location	single-SNP; rank	T-Trees; rank	hybrid; rank	single-SNP; p	T-Trees; vi	hybrid; vi
rs8011855	38673786	1	5	5	0	0.0298551353	0.0406287873
rs1957779	62739400	2	7	13	0	0.0284964121	0.0114005153
rs1457015	82704632	3	18	10	0.002	0.0092820857	0.0129045232
rs6574988	87629745	4	9	8	0.004	0.0175690479	0.0234725902
rs7142143	50473281	5	38	17	0.004	0.0028333437	0.0097743195
rs2653550	78672810	6	29	14	0.026	0.0038620063	0.0113375370
rs1253672	51459138	7	74	46	0.03	0.0007985511	0.0018276704
SNP A-2203090	32214885	8	30	49	0.217	0.0037660919	0.0016873485
rs6574590	80151227	9	61	44	0.443	0.0012010403	0.0022137415
rs17098915	61129684	11	41	36	0.486	0.0024481089	0.0038094737
rs10483456	35105918	13	97	29	0.628	0.0006267321	0.0050535673
rs1958305	23342964	21	8	12	0.889	0.0187541270	0.0122218227
rs1241649	24383933	22	68	40	0.916	0.0009770084	0.0027470373
rs435340	44004257	30	39	25	0.982	0.0027815511	0.0062562922
rs7149343	67634464	35	40	32	0.998	0.0027254172	0.0048198426
rs7150537	92331535	52	54	38	1	0.0014284773	0.0030667692
rs2194622	44358898	62	63	97	1	0.0011662734	0.0007270501
rs2400997	100796860	66	62	61	1	0.0011882915	0.0013264366

9 Rheumatoid arthritis, Chromosome 6

Table 9.1: Rheumatoid arthritis, Chromosome 6. Single-SNP and T-Trees approaches.

SNP identifier	Location	single-SNP; rank	T-Trees; rank	single-SNP; p	T-Trees; vi
rs10499044	107247988	1	4	0	0.0630765559
rs2677821	133963704	1	7	0	0.0520663246
rs2282859	167365129	1	9	0	0.0163503373
rs9271850	32703038	1	1	0	0.1585047003
rs3129768	32703061	1	3	0	0.0841176743
rs9272346	32712350	6	13	0	0.0067641638
rs9272723	32717405	7	22	0.003	0.0024888724
rs2876370	137958930	8	45	0.006	0.0006254908
rs943081	43988645	11	89	0.176	0.0002823740
rs7749323	138272082	15	58	0.48	0.0004864582
rs2523691	31528666	19	12	0.735	0.0088735015
rs2394102	28619996	20	25	0.806	0.0016567098
rs411136	33516520	25	38	0.934	0.0007800042
rs12174860	64974391	30	39	0.999	0.0007600187
rs6908950	42834327	66	67	1	0.0004135093

Table 9.2: Rheumatoid arthritis, Chromosome 6. Single-SNP and hybrid approaches.

SNP identifier	Location	single-SNP; rank	hybrid; rank	single-SNP; p	hybrid; vi
rs10499044	107247988	1	4	0	0.0697904323
rs2677821	133963704	1	3	0	0.0726410123
rs2282859	167365129	1	10	0	0.0286484387
rs9271850	32703038	1	1	0	0.1709058105
rs3129768	32703061	1	5	0	0.0553963826
rs9272346	32712350	6	14	0	0.0097011948
rs9272723	32717405	7	28	0.003	0.0041009373
rs2876370	137958930	8	18	0.006	0.0064546828
rs943081	43988645	11	34	0.176	0.0018685358
rs649371	101902099	13	68	0.386	0.0006856570
rs2523691	31528666	19	81	0.735	0.0005068029
rs10901001	7044907	21	48	0.85	0.0010537360
rs12174860	64974391	30	22	0.999	0.0048832518
rs3935973	153966274	82	64	1	0.0007215571

Table 9.3: Rheumatoid arthritis, Chromosome 6. T-Trees and hybrid approaches.

SNP identifier	Location	T-Trees; rank	hybrid; rank	T-Trees; vi	hybrid; vi
rs9271850	32703038	1	1	0.1585047003	0.1709058105
rs2677822	133965225	2	2	0.1499051565	0.0913054020
rs3129768	32703061	3	5	0.0841176743	0.0553963826
rs10499044	107247988	4	4	0.0630765559	0.0697904323
rs2798360	107255166	5	6	0.0621343160	0.0516132826
rs2636593	133969595	6	7	0.0577148653	0.0320767583
rs2677821	133963704	7	3	0.0520663246	0.0726410123
rs2677826	133968280	8	12	0.0287481707	0.0167622400
rs2282859	167365129	9	10	0.0163503373	0.0286484387
rs16899788	167351316	10	62	0.0156064698	0.0007507733
rs2677828	133969581	11	17	0.0147640241	0.0065733568
rs2523691	31528666	12	81	0.0088735015	0.0005068029
rs9272346	32712350	13	14	0.0067641638	0.0097011948
rs10947855	40204867	14	21	0.0066475358	0.0051079790
rs926925	107263554	15	19	0.0056781749	0.0061489462
rs6940205	107247939	16	26	0.0045098964	0.0042932206
rs9293855	72678221	17	8	0.0036580787	0.0318866196
rs10947857	40219493	20	13	0.0025168944	0.0119775528
rs2091113	40207363	21	25	0.0025049909	0.0046497451
rs9272723	32717405	22	28	0.0024888724	0.0041009373
rs940357	40220640	27	27	0.0014024941	0.0042633019
rs945238	84222575	29	16	0.0013529028	0.0086155938
rs2144375	107283239	30	32	0.0012705340	0.0020015335
rs10947856	40219405	36	23	0.0008406068	0.0048329116
rs2798361	107261138	37	49	0.0008063102	0.0010288992
rs12174860	64974391	39	22	0.0007600187	0.0048832518
rs2876370	137958930	45	18	0.0006254908	0.0064546828
rs9344825	64940889	66	45	0.0004240841	0.0013068128
rs1891698	72689749	70	15	0.0003964824	0.0086365575
rs195386	37441296	71	20	0.0003856604	0.0053995964
rs7745251	84228475	73	30	0.0003797863	0.0025254759
rs9449623	84237074	81	50	0.0003389565	0.0010005311
rs6903322	84225687	84	35	0.0003113477	0.0018601592
rs943081	43988645	89	34	0.0002823740	0.0018685358
rs4710471	64983570	90	38	0.0002798386	0.0015960259

Table 9.4: Rheumatoid arthritis, Chromosome 6. Single-SNP, T-Trees and hybrid approaches.

SNP identifier	Location	single-SNP; rank	T-Trees; rank	hybrid; rank	single-SNP; p	T-Trees; vi	hybrid; vi
rs10499044	107247988	1	4	4	0	0.0630765559	0.0697904323
rs2677821	133963704	1	7	3	0	0.0520663246	0.0726410123
rs2282859	167365129	1	9	10	0	0.0163503373	0.0286484387
rs9271850	32703038	1	1	1	0	0.1585047003	0.1709058105
rs3129768	32703061	1	3	5	0	0.0841176743	0.0553963826
rs9272346	32712350	6	13	14	0	0.0067641638	0.0097011948
rs9272723	32717405	7	22	28	0.003	0.0024888724	0.0041009373
rs2876370	137958930	8	45	18	0.006	0.0006254908	0.0064546828
rs943081	43988645	11	89	34	0.176	0.0002823740	0.0018685358
rs2523691	31528666	19	12	81	0.735	0.0088735015	0.0005068029
rs12174860	64974391	30	39	22	0.999	0.0007600187	0.0048832518

10 Rheumatoid arthritis, Chromosome 19

Table 10.1: Rheumatoid arthritis, Chromosome 19. Single-SNP and T-Trees approaches.

SNP identifier	Location	single-SNP; rank	T-Trees; rank	single-SNP; p	T-Trees; vi
rs11671119	19147077	1	1	0	0.3720436110
rs8112647	37846151	2	2	0	0.0275005327
rs7260239	51127649	3	5	0.071	0.0055668845
rs16968393	38869515	4	4	0.096	0.0060452119
rs10403038	24032496	5	35	0.187	0.0011845548
rs4646530	15596238	7	18	0.454	0.0018113494
rs443239	47197884	11	15	0.847	0.0021266099
rs1045354	9134300	12	56	0.874	0.0009004796
rs892188	10270793	18	74	0.993	0.0007008795
rs1076404	37384171	20	9	0.994	0.0046168709
rs7254234	60701027	21	41	0.996	0.0010862642
rs184583	35063430	23	50	0.996	0.0009567363
rs8112449	10381064	26	19	0.999	0.0016591026
rs3892630	37873324	30	10	1	0.0042354168
rs10418932	45492128	31	53	1	0.0009272069
rs310451	60742656	32	49	1	0.0009832866
rs8112226	6098939	34	46	1	0.0010408028
rs17774647	37323372	40	13	1	0.0022717052
rs16979989	50936008	44	70	1	0.0007252746
rs279223	11852638	50	75	1	0.0006980925
SNP A-1915656	303660	52	20	1	0.0016073725
rs1673894	57667055	53	24	1	0.0015184478
rs731674	37824235	63	42	1	0.0010802834
rs1673130	9996687	73	12	1	0.0023150843
rs11878322	37814726	76	36	1	0.0011725845
rs7249392	38782717	85	31	1	0.0012429847
rs17633799	57877343	94	72	1	0.0007047075

Table 10.2: Rheumatoid arthritis, Chromosome 19. Single-SNP and hybrid approaches.

SNP identifier	Location	single-SNP; rank	hybrid; rank	single-SNP; p	hybrid; vi
rs11671119	19147077	1	1	0	0.3901345027
rs8112647	37846151	2	3	0	0.0257925134
rs7260239	51127649	3	10	0.071	0.0045807162
rs16968393	38869515	4	22	0.096	0.0029202441
rs10403038	24032496	5	95	0.187	0.0005818320
rs4646530	15596238	7	7	0.454	0.0092447061
rs10409884	49398714	9	16	0.6	0.0036151720
rs443239	47197884	11	45	0.847	0.0013825310
rs1045354	9134300	12	60	0.874	0.0010616469
rs7249376	11691764	15	62	0.966	0.0009761742
rs1076404	37384171	20	86	0.994	0.0006455583
rs7254234	60701027	21	89	0.996	0.0006222316
rs184583	35063430	23	85	0.996	0.0006490994
rs677449	15660276	27	31	1	0.0024215733
rs10418932	45492128	31	56	1	0.0011442688
rs8112226	6098939	34	33	1	0.0021449315
rs12975585	46462071	45	64	1	0.0008909251
rs279223	11852638	50	74	1	0.0007193191
rs1673894	57667055	53	24	1	0.0027686316
rs7258789	20581344	58	51	1	0.0012513947

Table 10.3: Rheumatoid arthritis, Chromosome 19. T-Trees and hybrid approaches.

SNP identifier	Location	T-Trees; rank	hybrid; rank	T-Trees; vi	hybrid; vi
rs11671119	19147077	1	1	0.3720436110	0.3901345027
rs8112647	37846151	2	3	0.0275005327	0.0257925134
rs11668677	19202446	3	4	0.0139429729	0.0152035752
rs16968393	38869515	4	22	0.0060452119	0.0029202441
rs7260239	51127649	5	10	0.0055668845	0.0045807162
rs11672216	19205579	6	6	0.0053608038	0.0098478791
rs1076404	37384171	9	86	0.0046168709	0.0006455583
rs443239	47197884	15	45	0.0021266099	0.0013825310
rs7259415	53459838	16	19	0.0020417737	0.0032162383
rs4646530	15596238	18	7	0.0018113494	0.0092447061
SNP A-1945835	50240095	21	13	0.0015876964	0.0038014393
rs1673894	57667055	24	24	0.0015184478	0.0027686316
rs11882889	19748685	26	77	0.0013623158	0.0006973072
rs10403038	24032496	35	95	0.0011845548	0.0005818320
rs2304195	50254711	37	14	0.0011696061	0.0036885123
rs7254234	60701027	41	89	0.0010862642	0.0006222316
SNP A-4259473	33492335	44	42	0.0010535459	0.0016497394
rs8112226	6098939	46	33	0.0010408028	0.0021449315
rs8107912	18568048	47	20	0.0010401068	0.0030582223
rs184583	35063430	50	85	0.0009567363	0.0006490994
rs10418932	45492128	53	56	0.0009272069	0.0011442688
rs8106386	42195739	55	32	0.0009026200	0.0023474049
rs1045354	9134300	56	60	0.0009004796	0.0010616469
rs1433083	57085796	57	40	0.0008958585	0.0017883333
rs7258589	18562499	61	18	0.0008182686	0.0032744965
rs10403916	6852242	62	94	0.0007853434	0.0005881501
rs6508757	43214032	71	11	0.0007224421	0.0040859289
rs4805150	33496876	73	37	0.0007027044	0.0020130667
rs279223	11852638	75	74	0.0006980925	0.0007193191
rs2285669	62811576	79	27	0.0006574157	0.0025605626
rs4801754	53456669	91	35	0.0006159064	0.0020959084
rs12974182	50255095	98	17	0.0005909352	0.0034493822

Table 10.4: Rheumatoid arthritis, Chromosome 19. Single-SNP, T-Trees and hybrid approaches.

SNP identifier	Location	single-SNP; rank	T-Trees; rank	hybrid; rank	single-SNP; p	T-Trees; vi	hybrid; vi
rs11671119	19147077	1	1	1	0	0.3720436110	0.3901345027
rs8112647	37846151	2	2	3	0	0.0275005327	0.0257925134
rs7260239	51127649	3	5	10	0.071	0.0055668845	0.0045807162
rs16968393	38869515	4	4	22	0.096	0.0060452119	0.0029202441
rs10403038	24032496	5	35	95	0.187	0.0011845548	0.0005818320
rs4646530	15596238	7	18	7	0.454	0.0018113494	0.0092447061
rs443239	47197884	11	15	45	0.847	0.0021266099	0.0013825310
rs1045354	9134300	12	56	60	0.874	0.0009004796	0.0010616469
rs1076404	37384171	20	9	86	0.994	0.0046168709	0.0006455583
rs7254234	60701027	21	41	89	0.996	0.0010862642	0.0006222316
rs184583	35063430	23	50	85	0.996	0.0009567363	0.0006490994
rs10418932	45492128	31	53	56	1	0.0009272069	0.0011442688
rs8112226	6098939	34	46	33	1	0.0010408028	0.0021449315
rs279223	11852638	50	75	74	1	0.0006980925	0.0007193191
rs1673894	57667055	53	24	24	1	0.0015184478	0.0027686316

11 Type 1 diabetes, Chromosome 2

Table 11.1: Type 1 diabetes, Chromosome 2. Single-SNP and T-Trees approaches.

SNP identifier	Location	single-SNP; rank	T-Trees; rank	single-SNP; p	T-Trees; vi
rs17007623	61334969	1	5	0.001	0.0133742298
rs934776	85858744	2	11	0.005	0.0071723776
rs12476453	4118090	3	60	0.014	0.0006280073
rs903228	53545553	4	7	0.19	0.0110726358
rs17595217	205261744	7	96	0.633	0.0004820676
rs16857192	133613479	10	20	0.738	0.0019886588
SNP A-2312906	145636667	15	15	0.869	0.0031340497
rs6750794	117897430	21	26	0.967	0.0014973507
rs16845023	141433533	22	52	0.972	0.0006620969
rs2681035	23087764	25	17	0.979	0.0029656365
rs4848687	121639597	28	18	0.988	0.0024862257
rs4233826	169942170	37	64	1	0.0006067195
rs951840	49814673	46	43	1	0.0007557003
rs4266033	194870695	47	40	1	0.0008232962
rs16849921	213769267	48	1	1	0.0764485701
SNP A-1884336	215488779	51	46	1	0.0007323230
rs6546086	64625650	62	67	1	0.0005941744
rs2631835	54828975	73	79	1	0.0005236112
rs2216941	161966951	78	86	1	0.0005129494
rs4553871	134182116	82	68	1	0.0005913888
rs2218549	46291323	91	13	1	0.0042034781

Table 11.2: Type 1 diabetes, Chromosome 2. Single-SNP and hybrid approaches.

SNP identifier	Location	single-SNP; rank	hybrid; rank	single-SNP; p	hybrid; vi
rs17007623	61334969	1	2	0.001	0.0195961864
rs934776	85858744	2	4	0.005	0.0172738101
rs12476453	4118090	3	15	0.014	0.0027831733
rs903228	53545553	4	64	0.19	0.0011338601
rs13395488	26133533	5	37	0.259	0.0015279736
rs17595217	205261744	7	21	0.633	0.0023279291
rs16857192	133613479	10	20	0.738	0.0023510407
SNP A-2312906	145636667	15	36	0.869	0.0015337774
rs6750794	117897430	21	51	0.967	0.0012443585
rs2681035	23087764	25	38	0.979	0.0015269817
rs4848687	121639597	28	83	0.988	0.0008971243
rs6715456	160481745	43	90	1	0.0008462966
rs951840	49814673	46	91	1	0.0008458140
rs4266033	194870695	47	59	1	0.0011828586
rs6546086	64625650	62	77	1	0.0009348953
rs4675721	200187784	76	96	1	0.0008076576
rs4553871	134182116	82	79	1	0.0009172388
rs2218549	46291323	91	9	1	0.0065853168

Table 11.3: Type 1 diabetes, Chromosome 2. T-Trees and hybrid approaches.

SNP identifier	Location	T-Trees; rank	hybrid; rank	T-Trees; vi	hybrid; vi
rs350753	52727292	4	3	0.0135427010	0.0185427702
rs17007623	61334969	5	2	0.0133742298	0.0195961864
rs903228	53545553	7	64	0.0110726358	0.0011338601
rs16849743	213680911	8	49	0.0099360752	0.0012742642
rs281515	46283937	9	5	0.0091922578	0.0132344797
rs934776	85858744	11	4	0.0071723776	0.0172738101
rs6733006	12233521	12	6	0.0060456965	0.0090108980
rs2218549	46291323	13	9	0.0042034781	0.0065853168
rs12477230	195501468	14	10	0.0040155435	0.0060546039
SNP A-2312906	145636667	15	36	0.0031340497	0.0015337774
rs350747	52722160	16	8	0.0029667298	0.0068625049
rs2681035	23087764	17	38	0.0029656365	0.0015269817
rs4848687	121639597	18	83	0.0024862257	0.0008971243
rs16857192	133613479	20	20	0.0019886588	0.0023510407
rs6738339	36091824	22	41	0.0019447442	0.0013883102
rs6432267	12235917	23	13	0.0019243118	0.0032130392
rs6750794	117897430	26	51	0.0014973507	0.0012443585
rs1401798	151382117	29	18	0.0012615228	0.0025251333
rs10495773	29705592	32	82	0.0010374234	0.0009025027
rs17035249	109233135	35	11	0.0009324738	0.0048936780
rs12611555	195499865	39	23	0.0008389889	0.0021715346
rs4266033	194870695	40	59	0.0008232962	0.0011828586
rs10205397	29705768	42	27	0.0007932046	0.0020350401
rs951840	49814673	43	91	0.0007557003	0.0008458140
rs1564811	22620940	47	7	0.0007250596	0.0069881948
rs17439859	194266604	48	74	0.0007247955	0.0009769530
rs7422734	233980531	57	12	0.0006413106	0.0032500797
rs12476453	4118090	60	15	0.0006280073	0.0027831733
rs10930291	151390032	66	35	0.0006010024	0.0016383427
rs6546086	64625650	67	77	0.0005941744	0.0009348953
rs4553871	134182116	68	79	0.0005913888	0.0009172388
rs11892545	234003674	72	25	0.0005836156	0.0020484492
rs2568569	237461131	73	16	0.0005614004	0.0026016272
SNP A-2058063	16405194	78	56	0.0005319440	0.0012172192
rs2705725	183703781	88	40	0.0005031745	0.0014236292
rs2705762	183717921	90	44	0.0004935099	0.0013183771
rs2062339	22630513	92	19	0.0004902167	0.0023600238
rs17595217	205261744	96	21	0.0004820676	0.0023279291

Table 11.4: Type 1 diabetes, Chromosome 2. Single-SNP, T-Trees and hybrid approaches.

SNP identifier	Location	single-SNP; rank	T-Trees; rank	hybrid; rank	single-SNP; p	T-Trees; vi	hybrid; vi
rs17007623	61334969	1	5	2	0.001	0.0133742298	0.0195961864
rs934776	85858744	2	11	4	0.005	0.0071723776	0.0172738101
rs12476453	4118090	3	60	15	0.014	0.0006280073	0.0027831733
rs903228	53545553	4	7	64	0.19	0.0110726358	0.0011338601
rs17595217	205261744	7	96	21	0.633	0.0004820676	0.0023279291
rs16857192	133613479	10	20	20	0.738	0.0019886588	0.0023510407
SNP A-2312906	145636667	15	15	36	0.869	0.0031340497	0.0015337774
rs6750794	117897430	21	26	51	0.967	0.0014973507	0.0012443585
rs2681035	23087764	25	17	38	0.979	0.0029656365	0.0015269817
rs4848687	121639597	28	18	83	0.988	0.0024862257	0.0008971243
rs951840	49814673	46	43	91	1	0.0007557003	0.0008458140
rs4266033	194870695	47	40	59	1	0.0008232962	0.0011828586
rs6546086	64625650	62	67	77	1	0.0005941744	0.0009348953
rs4553871	134182116	82	68	79	1	0.0005913888	0.0009172388
rs2218549	46291323	91	13	9	1	0.0042034781	0.0065853168

12 Type 1 diabetes, Chromosome 13

Table 12.1: Type 1 diabetes, Chromosome 13. Single-SNP and T-Trees approaches.

SNP identifier	Location	single-SNP; rank	T-Trees; rank	single-SNP; p	T-Trees; vi
rs4254200	82213653	1	2	0	0.2538874379
rs16946406	91101609	2	15	0.069	0.0028103972
rs7317472	36700636	4	16	0.13	0.0025256870
rs3002099	18999964	5	57	0.13	0.0003716672
rs9514597	106408722	6	18	0.336	0.0020581485
rs12708382	101444814	7	25	0.344	0.0008447923
rs391286	92654382	10	95	0.673	0.0002249988
rs9315704	39038215	12	53	0.927	0.0004241478
rs2325562	73245093	20	33	0.999	0.0006724809
rs1764773	104213047	24	41	1	0.0005124660
rs288700	106336283	30	19	1	0.0014049832
rs1474085	36045906	44	59	1	0.0003579405
rs17710571	98350724	55	55	1	0.0003843752
rs2759295	36107815	59	30	1	0.0007320793
rs9559306	107804756	61	49	1	0.0004641363
rs17068485	36799218	85	23	1	0.0010976141
rs7326796	105075378	86	94	1	0.0002300097
rs1499482	62112341	86	44	1	0.0004760837

Table 12.2: Type 1 diabetes, Chromosome 13. Single-SNP and hybrid approaches.

SNP identifier	Location	single-SNP; rank	hybrid; rank	single-SNP; p	hybrid; vi
rs4254200	82213653	1	1	0	0.3727425083
rs16946406	91101609	2	8	0.069	0.0061699275
rs4769283	23199943	3	42	0.118	0.0007401564
rs7317472	36700636	4	17	0.13	0.0029329278
rs3002099	18999964	5	22	0.13	0.0020762461
rs9514597	106408722	6	19	0.336	0.0027848160
rs12708382	101444814	7	20	0.344	0.0027549027
rs4238171	23199897	8	94	0.42	0.0003128257
rs391286	92654382	10	55	0.673	0.0005268828
rs9315704	39038215	12	30	0.927	0.0011241682
rs7337081	59229164	13	92	0.927	0.0003146352
rs2325562	73245093	20	45	0.999	0.0007147068
rs1764773	104213047	24	50	1	0.0005994729
rs6563675	38597260	29	84	1	0.0003444495
rs288700	106336283	30	65	1	0.0004403043
rs9506153	19007717	35	97	1	0.0003053746
rs17068485	36799218	85	81	1	0.0003465394

Table 12.3: Type 1 diabetes, Chromosome 13. T-Trees and hybrid approaches.

SNP identifier	Location	T-Trees; rank	hybrid; rank	T-Trees; vi	hybrid; vi
rs7332105	82242107	1	2	0.2821058812	0.3183339312
rs4254200	82213653	2	1	0.2538874379	0.3727425083
rs9601986	82299099	3	9	0.0518548910	0.0059502062
rs9546237	82312847	4	4	0.0437315693	0.0221687262
rs9546233	82296702	5	7	0.0236612782	0.0082771597
rs9601965	82215515	6	6	0.0202709847	0.0088607060
rs2129717	82325708	7	5	0.0144343594	0.0133027603
rs4884202	82305944	8	11	0.0138125806	0.0050097536
rs4457887	82298113	9	88	0.0113226469	0.0003305281
rs17260020	82217462	10	13	0.0104194331	0.0036862327
rs7321737	82312521	11	15	0.0076412257	0.0034544181
rs9546265	82383339	13	34	0.0053496609	0.0010003217
rs16946406	91101609	15	8	0.0028103972	0.0061699275
rs7317472	36700636	16	17	0.0025256870	0.0029329278
rs4885849	82312815	17	25	0.0020655485	0.0016770407
rs9514597	106408722	18	19	0.0020581485	0.0027848160
rs288700	106336283	19	65	0.0014049832	0.0004403043
rs11619882	82202815	20	12	0.0012263563	0.0046073636
rs17068485	36799218	23	81	0.0010976141	0.0003465394
rs12708382	101444814	25	20	0.0008447923	0.0027549027
rs1336672	68589901	27	37	0.0008169773	0.0009645952
rs389862	113948331	31	23	0.0007132331	0.0019890787
rs2325562	73245093	33	45	0.0006724809	0.0007147068
rs17074143	49820774	35	36	0.0006228724	0.0009747575
rs568040	74814381	36	67	0.0006187147	0.0004374816
rs3955211	113932906	38	14	0.0005641328	0.0035711155
rs16946362	91096839	40	27	0.0005191700	0.0012098514
rs1764773	104213047	41	50	0.0005124660	0.0005994729
rs11842790	49822449	43	31	0.0004855199	0.0010998894
rs869878	74180896	46	26	0.0004689932	0.0012645742
rs9525291	113962630	47	10	0.0004676263	0.0055976782
rs9315704	39038215	53	30	0.0004241478	0.0011241682
rs3002099	18999964	57	22	0.0003716672	0.0020762461
rs16946371	91098220	62	33	0.0003354315	0.0010420930
rs12874278	49833923	64	63	0.0003262154	0.0004556969
rs2875569	68573704	65	69	0.0003089596	0.0004241528
rs1328369	92710991	75	71	0.0002748044	0.0004032215
rs9575092	81857944	85	74	0.0002369022	0.0003846520
rs9539942	63607715	89	24	0.0002355829	0.0017196686
rs391286	92654382	95	55	0.0002249988	0.0005268828

Table 12.4: Type 1 diabetes, Chromosome 13. Single-SNP, T-Trees and hybrid approaches.

SNP identifier	Location	single-SNP; rank	T-Trees; rank	hybrid; rank	single-SNP; p	T-Trees; vi	hybrid; vi
rs4254200	82213653	1	2	1	0	0.2538874379	0.3727425083
rs16946406	91101609	2	15	8	0.069	0.0028103972	0.0061699275
rs7317472	36700636	4	16	17	0.13	0.0025256870	0.0029329278
rs3002099	18999964	5	57	22	0.13	0.0003716672	0.0020762461
rs9514597	106408722	6	18	19	0.336	0.0020581485	0.0027848160
rs12708382	101444814	7	25	20	0.344	0.0008447923	0.0027549027
rs391286	92654382	10	95	55	0.673	0.0002249988	0.0005268828
rs9315704	39038215	12	53	30	0.927	0.0004241478	0.0011241682
rs2325562	73245093	20	33	45	0.999	0.0006724809	0.0007147068
rs1764773	104213047	24	41	50	1	0.0005124660	0.0005994729
rs288700	106336283	30	19	65	1	0.0014049832	0.0004403043
rs17068485	36799218	85	23	81	1	0.0010976141	0.0003465394

13 Type 2 diabetes, Chromosome 10

Table 13.1: Type 2 diabetes, Chromosome 10. Single-SNP and T-Trees approaches.

SNP identifier	Location	single-SNP; rank	T-Trees; rank	single-SNP; p	T-Trees; vi
rs4506565	114746031	1	27	0	0.0024009775
rs7901695	114744078	2	17	0	0.0034539411
rs7077039	114779067	3	13	0	0.0047124557
rs4132670	114757761	4	38	0	0.0014877667
rs10787472	114771287	5	75	0	0.0007878316
rs11196205	114797037	6	84	0	0.0007051230
rs4074720	114738487	9	53	0	0.0009835015
rs12243326	114778805	10	60	0	0.0008930770
rs2479037	114586715	11	1	0.001	0.0186489590
rs17094393	117981552	12	4	0.001	0.0124231173
rs11006039	59495478	14	3	0.004	0.0124704060
rs293284	52888865	16	7	0.14	0.0079004995
rs7898565	99965717	17	11	0.256	0.0049178430
rs7082404	77116071	21	5	0.371	0.0115409548
rs1691907	29165925	22	80	0.386	0.0007492030
rs11190376	101788618	24	46	0.43	0.0011934485
rs7894018	92807259	27	12	0.678	0.0048441347
rs7079515	75702417	31	39	0.752	0.0014343174
rs7067701	125762409	32	54	0.801	0.0009695387
rs3120134	132136144	34	45	0.91	0.0012026737
rs2813383	1611800	41	20	0.989	0.0031712388
rs1961317	120644012	47	25	0.999	0.0025425871
rs505699	99764066	53	88	0.999	0.0006645506
rs11011417	38311123	66	76	1	0.0007809889
rs12265675	84069725	67	68	1	0.0008582652
rs11185747	91289457	68	30	1	0.0017703881
rs7071056	112427620	70	66	1	0.0008655349
rs11193797	84083975	73	85	1	0.0007049151
rs1566222	70189609	76	14	1	0.0042274703
rs2250544	84437807	78	37	1	0.0014933012
rs2797887	14011662	90	64	1	0.0008748253

Table 13.2: Type 2 diabetes, Chromosome 10. Single-SNP and hybrid approaches.

SNP identifier	Location	single-SNP; rank	hybrid; rank	single-SNP; p	hybrid; vi
rs4506565	114746031	1	22	0	0.0043359677
rs7901695	114744078	2	26	0	0.0035669469
rs7077039	114779067	3	18	0	0.0055736223
rs4132670	114757761	4	32	0	0.0029378345
rs10787472	114771287	5	66	0	0.0013403116
rs11196205	114797037	6	89	0	0.0010264811
rs4074720	114738487	9	63	0	0.0014216117
rs12243326	114778805	10	75	0	0.0012491396
rs2479037	114586715	11	5	0.001	0.0160654034
rs17094393	117981552	12	7	0.001	0.0127614245
rs11006039	59495478	14	11	0.004	0.0068231524
rs293284	52888865	16	6	0.14	0.0130521405
rs7898565	99965717	17	24	0.256	0.0036458166
rs7082404	77116071	21	3	0.371	0.0224677955
rs11190376	101788618	24	74	0.43	0.0012635745
rs7894018	92807259	27	10	0.678	0.0110033719
rs7079515	75702417	31	81	0.752	0.0011615070
rs2813383	1611800	41	35	0.989	0.0026390874
rs1961317	120644012	47	34	0.999	0.0026937406
rs505699	99764066	53	79	0.999	0.0012017055
rs4751522	129487468	59	42	1	0.0022359218
rs12265675	84069725	67	71	1	0.0013029158
rs1566222	70189609	76	19	1	0.0055602542
rs2250544	84437807	78	50	1	0.0017425263

Table 13.3: Type 2 diabetes, Chromosome 10. T-Trees and hybrid approaches.

SNP identifier	Location	T-Trees; rank	hybrid; rank	T-Trees; vi	hybrid; vi
rs2479037	114586715	1	5	0.0186489590	0.0160654034
SNP A-1995641	12837834	2	4	0.0144765300	0.0166075274
rs11006039	59495478	3	11	0.0124704060	0.0068231524
rs17094393	117981552	4	7	0.0124231173	0.0127614245
rs7082404	77116071	5	3	0.0115409548	0.0224677955
rs11001473	77124024	6	21	0.0098293208	0.0047821488
rs293284	52888865	7	6	0.0079004995	0.0130521405
SNP A-4229534	12834342	8	8	0.0072252082	0.0126263904
rs10458641	77076960	9	70	0.0052632180	0.0013107205
rs1300253	70172294	10	17	0.0051541454	0.0057200317
rs7898565	99965717	11	24	0.0049178430	0.0036458166
rs7894018	92807259	12	10	0.0048441347	0.0110033719
rs7077039	114779067	13	18	0.0047124557	0.0055736223
rs1566222	70189609	14	19	0.0042274703	0.0055602542
rs7078534	28163722	15	14	0.0040911775	0.0061282312
rs661882	27808089	16	9	0.0039385052	0.0115830618
rs7901695	114744078	17	26	0.0034539411	0.0035669469
rs10998441	70235782	18	28	0.0034304178	0.0034277016
rs1707275	17049108	19	23	0.0032524130	0.0039492475
rs2813383	1611800	20	35	0.0031712388	0.0026390874
rs7923177	120596880	21	27	0.0027120674	0.0035111446
rs6481484	28163077	22	39	0.0026141961	0.0023734183
rs10490898	28898895	24	13	0.0025442788	0.0063970801
rs1961317	120644012	25	34	0.0025425871	0.0026937406
rs1162961	70167488	26	30	0.0024952683	0.0030517085
rs4506565	114746031	27	22	0.0024009775	0.0043359677
rs17152197	12832547	28	25	0.0021905373	0.0036250076
rs6481485	28163370	29	37	0.0021335432	0.0026245212
rs717287	27819196	31	12	0.0017669186	0.0065604755
rs1875163	77146649	32	2	0.0017474227	0.0225038586
rs11186426	92802986	33	31	0.0017000400	0.0030378642
rs7073805	42401155	34	43	0.0016639702	0.0022160431
rs2250544	84437807	37	50	0.0014933012	0.0017425263
rs4132670	114757761	38	32	0.0014877667	0.0029378345
rs7079515	75702417	39	81	0.0014343174	0.0011615070
rs11190376	101788618	46	74	0.0011934485	0.0012635745
rs1177379	70242013	48	61	0.0011290743	0.0015429621
rs11186914	94052611	49	40	0.0011199417	0.0023342513
rs17099789	84078553	50	53	0.0010566324	0.0017046118
rs10881889	92878888	52	36	0.0010239256	0.0026383238
rs4074720	114738487	53	63	0.0009835015	0.0014216117
rs1408579	101902184	55	72	0.0009636572	0.0012980067
rs12243326	114778805	60	75	0.0008930770	0.0012491396
rs11595274	92806870	61	60	0.0008793893	0.0015565679
rs17659719	120216863	67	46	0.0008599878	0.0017760269
rs12265675	84069725	68	71	0.0008582652	0.0013029158
rs12254584	28491255	72	69	0.0007959385	0.0013155837
rs6583755	92881730	73	56	0.0007935223	0.0016248489
rs10787472	114771287	75	66	0.0007878316	0.0013403116
rs7067899	92881044	77	77	0.0007771569	0.0012204261
rs1977325	44645487	78	49	0.0007688877	0.0017524628
rs10829261	27815294	83	52	0.0007051432	0.0017161673
rs11196205	114797037	84	89	0.0007051230	0.0010264811
rs11254836	6955867	86	29	0.0006856660	0.0031594548
rs505699	99764066	88	79	0.0006645506	0.0012017055
rs7922670	117257300	89	83	0.0006627489	0.0011357387
rs12256867	42445906	90	80	0.0006622081	0.0011786815
rs1300252	70172432	93	68	0.0006370380	0.0013317020
rs6481534	28907403	95	15	0.0006318414	0.0059550486
rs7078615	125853202	100	44	0.0006244355	0.0018547924

Table 13.4: Type 2 diabetes, Chromosome 10. Single-SNP, T-Trees and hybrid approaches.

SNP identifier	Location	single-SNP; rank	T-Trees; rank	hybrid; rank	single-SNP; p	T-Trees; vi	hybrid; vi
rs4506565	114746031	1	27	22	0	0.0024009775	0.0043359677
rs7901695	114744078	2	17	26	0	0.0034539411	0.0035669469
rs7077039	114779067	3	13	18	0	0.0047124557	0.0055736223
rs4132670	114757761	4	38	32	0	0.0014877667	0.0029378345
rs10787472	114771287	5	75	66	0	0.0007878316	0.0013403116
rs11196205	114797037	6	84	89	0	0.0007051230	0.0010264811
rs4074720	114738487	9	53	63	0	0.0009835015	0.0014216117
rs12243326	114778805	10	60	75	0	0.0008930770	0.0012491396
rs2479037	114586715	11	1	5	0.001	0.0186489590	0.0160654034
rs17094393	117981552	12	4	7	0.001	0.0124231173	0.0127614245
rs11006039	59495478	14	3	11	0.004	0.0124704060	0.0068231524
rs293284	52888865	16	7	6	0.14	0.0079004995	0.0130521405
rs7898565	99965717	17	11	24	0.256	0.0049178430	0.0036458166
rs7082404	77116071	21	5	3	0.371	0.0115409548	0.0224677955
rs11190376	101788618	24	46	74	0.43	0.0011934485	0.0012635745
rs7894018	92807259	27	12	10	0.678	0.0048441347	0.0110033719
rs7079515	75702417	31	39	81	0.752	0.0014343174	0.0011615070
rs2813383	1611800	41	20	35	0.989	0.0031712388	0.0026390874
rs1961317	120644012	47	25	34	0.999	0.0025425871	0.0026937406
rs505699	99764066	53	88	79	0.999	0.0006645506	0.0012017055
rs12265675	84069725	67	68	71	1	0.0008582652	0.0013029158
rs1566222	70189609	76	14	19	1	0.0042274703	0.0055602542
rs2250544	84437807	78	37	50	1	0.0014933012	0.0017425263

14 Type 2 diabetes, Chromosome 21

Table 14.1: Type 2 diabetes, Chromosome 21. Single-SNP and T-Trees approaches.

SNP identifier	Location	single-SNP; rank	T-Trees; rank	single-SNP; p	T-Trees; vi
rs226261	26953847	1	1	0	0.2469128707
rs6517434	38109926	2	14	0.036	0.0035406228
rs7276641	25799569	3	52	0.578	0.0007131539
rs8130806	21330548	14	42	1	0.0008236536
rs2823980	16984585	15	44	1	0.0008007034
rs2284642	36573183	16	30	1	0.0011314788
rs16987706	15914939	18	54	1	0.0006963192
rs435260	35816526	20	97	1	0.0004865401
rs7279871	32366101	21	16	1	0.0025876713
rs16994356	36927512	25	48	1	0.0007691225
rs2836071	38259661	29	77	1	0.0005728948
rs468453	26550168	32	95	1	0.0004923763
rs2824392	17901426	35	55	1	0.0006870882
rs220161	42422362	38	50	1	0.0007314639
rs16990063	33310603	56	40	1	0.0008410824
rs3788147	45154097	62	58	1	0.0006588373
rs16997735	39792304	69	41	1	0.0008345043
rs6586252	43149456	70	92	1	0.0005148198
rs16991205	34394871	71	15	1	0.0029788628
rs9980699	34421519	83	23	1	0.0014508845
rs17000913	41730743	83	19	1	0.0018215928
rs764967	35221535	93	20	1	0.0017779083

Table 14.2: Type 2 diabetes, Chromosome 21. Single-SNP and hybrid approaches.

SNP identifier	Location	single-SNP; rank	hybrid; rank	single-SNP; p	hybrid; vi
rs226261	26953847	1	1	0	0.2108330173
rs7276641	25799569	3	53	0.578	0.0009841657
rs440544	35817449	7	96	0.995	0.0005945070
rs8130806	21330548	14	38	1	0.0014054434
rs435260	35816526	20	99	1	0.0005820725
rs16994356	36927512	25	51	1	0.0010050038
rs220161	42422362	38	77	1	0.0007198498
rs11700653	29039190	78	40	1	0.0012953870
rs17000913	41730743	83	78	1	0.0007088186
rs764967	35221535	93	25	1	0.0022457495

Table 14.3: Type 2 diabetes, Chromosome 21. T-Trees and hybrid approaches.

SNP identifier	Location	T-Trees; rank	hybrid; rank	T-Trees; vi	hybrid; vi
rs226261	26953847	1	1	0.2469128707	0.2108330173
rs2830320	26947555	2	23	0.1835595877	0.0023684141
rs458076	26947782	3	4	0.0449797759	0.0628404988
rs2830322	26948370	4	24	0.0177194392	0.0022828664
rs2174536	26947727	5	36	0.0132726310	0.0014300275
rs756166	26953857	6	15	0.0108233688	0.0034370234
rs2830326	26954058	7	27	0.0055137740	0.0021912151
rs2212809	26986061	8	5	0.0049550508	0.0421300663
rs2830414	26994734	9	3	0.0046927236	0.0641143140
rs2837630	40740413	10	9	0.0046009375	0.0095361008
rs996632	26952709	11	48	0.0044744794	0.0011047409
rs2830380	26979808	12	2	0.0043517277	0.0769505288
rs12482676	34892582	17	10	0.0022744763	0.0083298836
rs2837632	40741151	18	16	0.0022394250	0.0033563313
rs17000913	41730743	19	78	0.0018215928	0.0007088186
rs764967	35221535	20	25	0.0017779083	0.0022457495
rs2300390	34891385	21	11	0.0015250640	0.0070028958
rs2822691	14777301	26	14	0.0013243387	0.0035724472
rs2830419	27002006	27	7	0.0012879844	0.0221941641
rs2300391	34891770	36	12	0.0009542241	0.0043043588
rs2830395	26985677	37	8	0.0008715382	0.0169123444
rs9305551	34886954	39	20	0.0008532573	0.0025058114
rs8130806	21330548	42	38	0.0008236536	0.0014054434
rs2822690	14777098	45	17	0.0007909721	0.0032907644
rs16994356	36927512	48	51	0.0007691225	0.0010050038
rs220161	42422362	50	77	0.0007314639	0.0007198498
rs7276641	25799569	52	53	0.0007131539	0.0009841657
rs2822686	14773971	59	35	0.0006587709	0.0017062353
rs6517275	35373435	60	67	0.0006502088	0.0008014935
rs12626309	33810574	73	46	0.0005876859	0.0011695264
rs8133101	35358921	79	71	0.0005635671	0.0007731639
rs1033335	35506939	82	31	0.0005549890	0.0018016561
rs2835263	36352115	85	26	0.0005321157	0.0022290684
rs2834231	33814229	94	30	0.0004941234	0.0019419264
rs435260	35816526	97	99	0.0004865401	0.0005820725
rs2823768	16679197	98	21	0.0004772237	0.0024830213
rs2837654	40776531	100	33	0.0004757734	0.0017586414

Table 14.4: Type 2 diabetes, Chromosome 21. Single-SNP, T-Trees and hybrid approaches.

SNP identifier	Location	single-SNP; rank	T-Trees; rank	hybrid; rank	single-SNP; p	T-Trees; vi	hybrid; vi
rs226261	26953847	1	1	1	0	0.2469128707	0.2108330173
rs7276641	25799569	3	52	53	0.578	0.0007131539	0.0009841657
rs8130806	21330548	14	42	38	1	0.0008236536	0.0014054434
rs435260	35816526	20	97	99	1	0.0004865401	0.0005820725
rs16994356	36927512	25	48	51	1	0.0007691225	0.0010050038
rs220161	42422362	38	50	77	1	0.0007314639	0.0007198498
rs17000913	41730743	83	19	78	1	0.0018215928	0.0007088186
rs764967	35221535	93	20	25	1	0.0017779083	0.0022457495
