# Supplementary material for: A method combining a random forest-based technique with the modeling of linkage disequilibrium through latent variables, to run multilocus genome-wide association studies
Source: BMC Bioinformatics. 2018 Mar 27;19:106. doi: 10.1186/s12859-018-2054-0 (PMC5870262; doi:10.1186/s12859-018-2054-0)
Supplement: Supplementary file 3 — SNPs jointly identified in the top 100s by two or three methods among single-SNP, T-Trees and hybrid FLTM / T-Trees approaches. Venn diagrams. For each of the 14 datasets analyzed, a Venn diagram shows the number of top 100 SNPs jointly identified by any two among the three, or the three methods considered. (PDF 122 kb) [file 12859_2018_2054_MOESM3_ESM.pdf]

Supplementary data
Additional file 3

SNPs jointly identified in the top 100s by two or three methods among Single-SNP, T-Trees and hybrid FLTM / T-Trees approaches

Venn diagrams

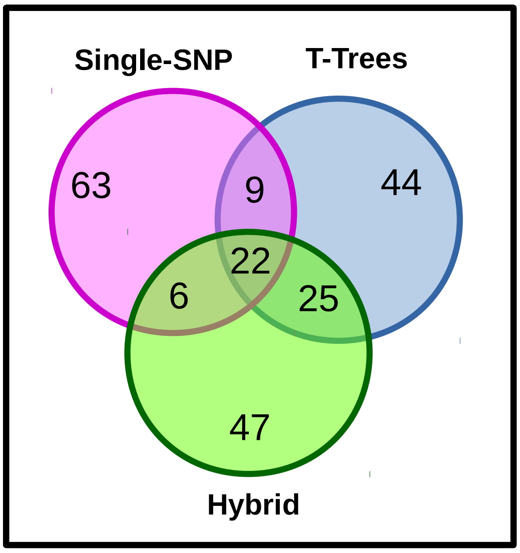

Bipolar disorder, Chromosome 03

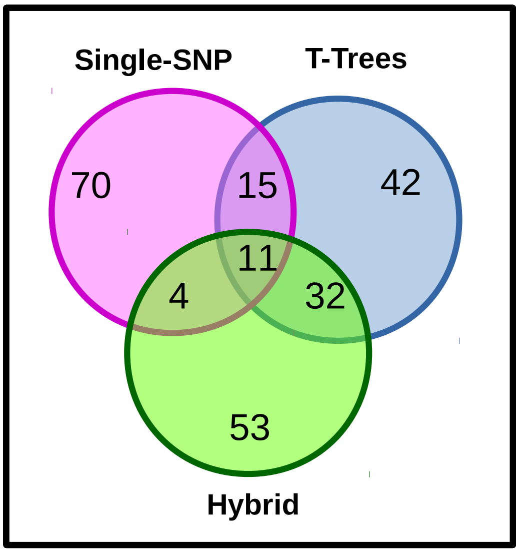

Bipolar disorder, Chromosome 21

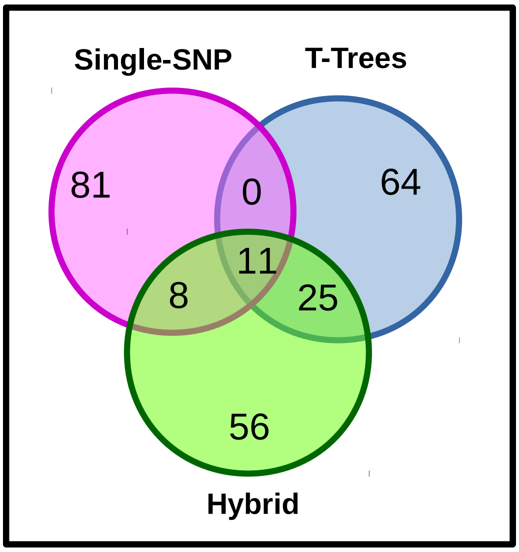

Coronary artery disease, Chromosome 05

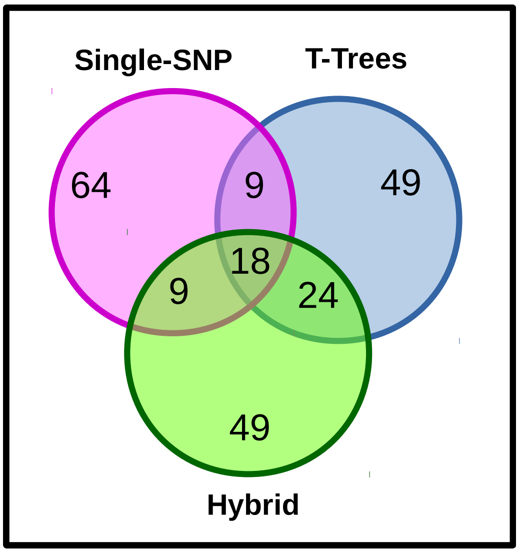

Coronary artery disease, Chromosome 06

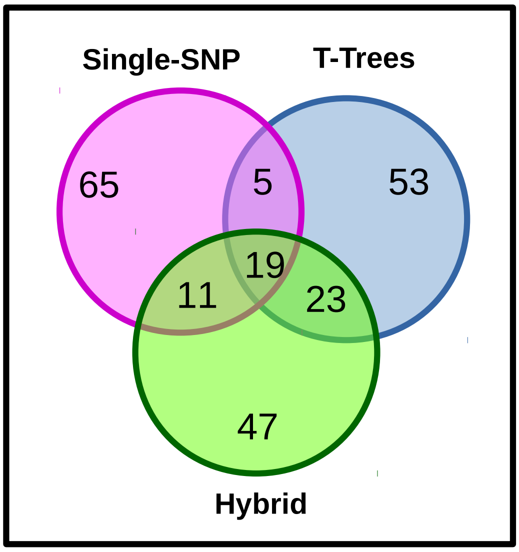

Crohn's disease, Chromosome 01

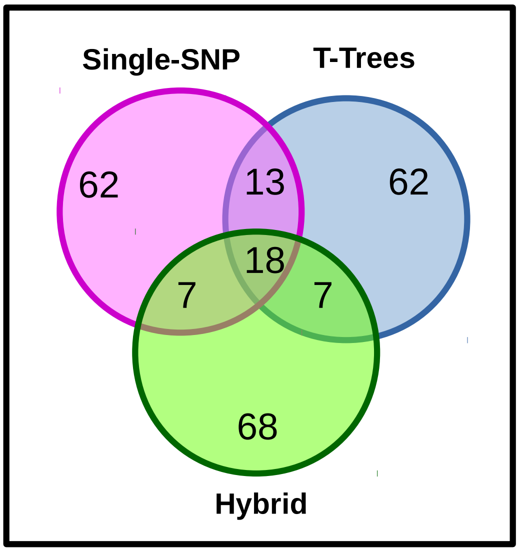

Crohn's disease, Chromosome 20

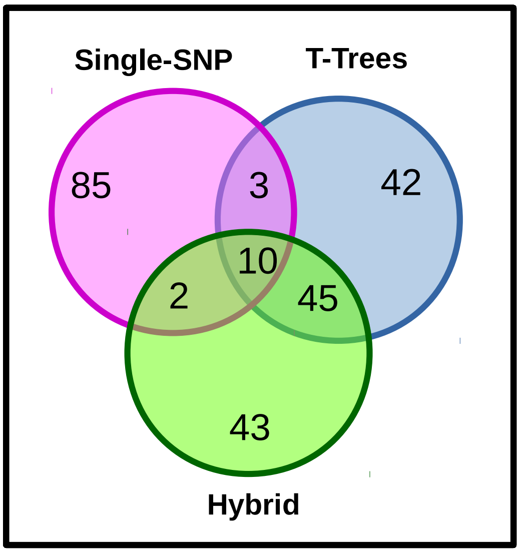

Hypertension,
Chromosome 10

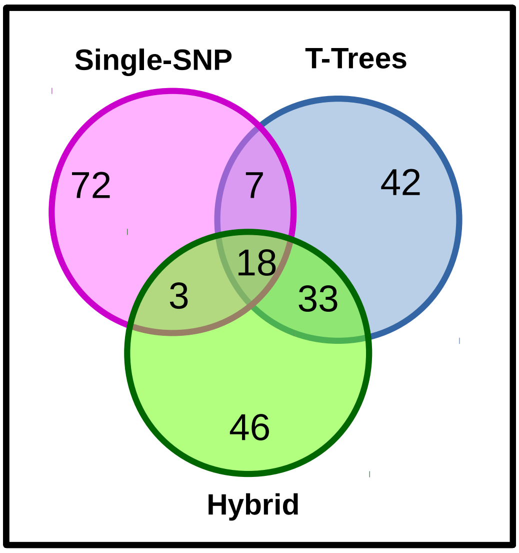

Hypertension,
Chromosome 14

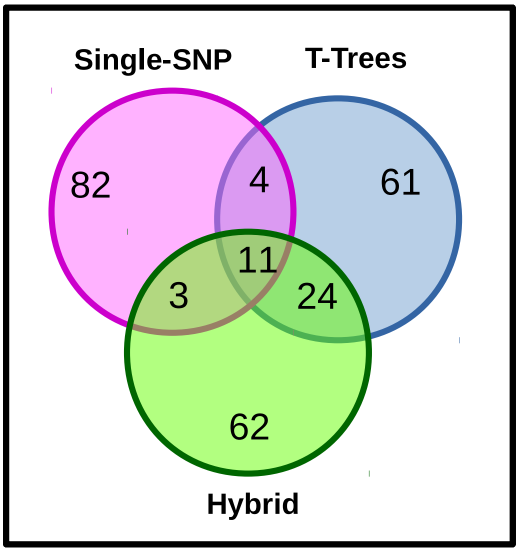

Rheumatoid arthritis,
Chromosome 06

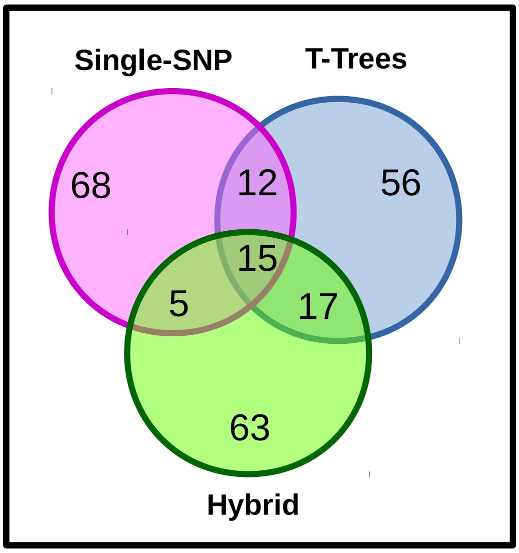

Rheumatoid arthritis,
Chromosome 19

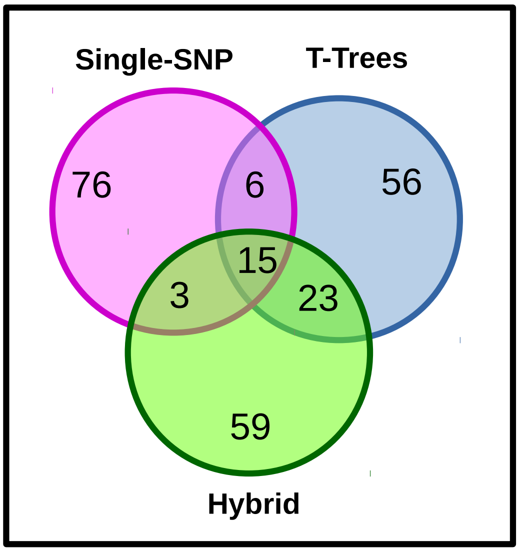

Type 1 diabetes,
Chromosome 02

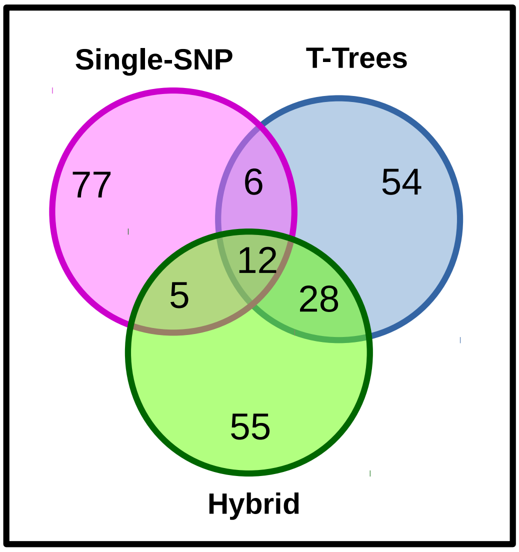

Type 1 diabetes,
Chromosome 13

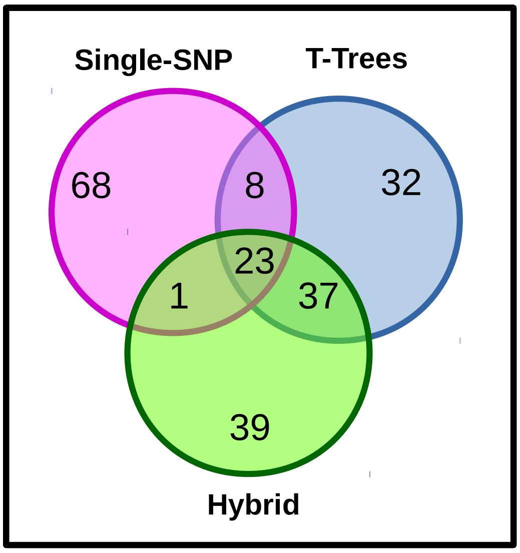

Type 2 diabetes, Chromosome 10

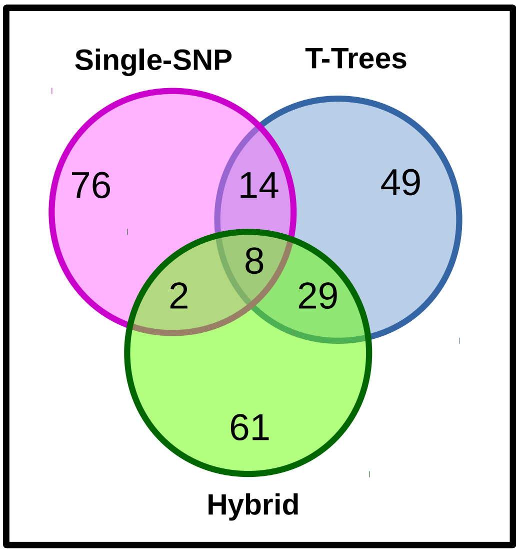

Type 2 diabetes, Chromosome 21

Comments

First, we observe that the intersections between single-SNP GWAS and T-Trees or single-SNP GWAS and the hybrid approach always show relatively small cardinalities (in the range [10,31]). The cardinalities of the intersections between single-SNP GWAS and T-Trees range in [11,31]. The cardinalities of the intersections between single-SNP GWAS and the hybrid approach vary between 10 and 30. In addition, except for three datasets over 14 ((CAD, Chr05), (CAD, Chr06) and (CD, Chr01)), the hybrid approach always shares strictly less top 100 SNPs with the Single-SNP GWAS than T-Trees. In this regard, the largest discrepancy (12) is observed for (T2D, Chr21); the smallest difference (0) is observed for (CAD, Chr06).

Second, the number of top 100 SNPs shared between T-Trees and the hybrid approach is higher than when the Single-SNP GWAS is involved in the comparison: the range is [25,60]. In particular, the two largest values of 55 and 60 are respectively observed for (HT, Chr10) and (TD2, Chr10).

Finally, the number of top 100 SNPs that are common to the three methods range in interval [8,23]. The minimum is observed for (T2D, Chr21). The maximum is observed for (T2D, Chr10).
