# Supplementary material for: A method combining a random forest-based technique with the modeling of linkage disequilibrium through latent variables, to run multilocus genome-wide association studies
Source: BMC Bioinformatics. 2018 Mar 27;19:106. doi: 10.1186/s12859-018-2054-0 (PMC5870262; doi:10.1186/s12859-018-2054-0)
Supplement: Supplementary file 4 — Characteristics of the SNPs jointly identified as top 100s by the Single-SNP, T-Trees and hybrid FLTM / T-Trees approaches. Table 1: Characteristics of the SNPs jointly identified as top 100s by the Single-SNP, T-Trees and hybrid FLTM / T-Trees approaches. (PDF 81.6 kb) [file 12859_2018_2054_MOESM4_ESM.pdf]

Supplementary data
Additional file 4

Characteristics of the SNPs jointly identified as top 100s by the Single-SNP, T-Trees and hybrid FLTM / T-Trees approaches.

Table 1. Characteristics of the SNPs jointly identified as top 100s by the Single-SNP, T-Trees and hybrid FLTM / T-Trees approaches. S : Single-SNP GWAS. T : T-Trees approach. H : hybrid approach.

Pathology, chromosome	SNP identifier	Location	Rank			Corrected p-value (p) or variable importance (vi)		
			S	T	H	Single-SNP p	T-Trees vi	Hybrid vi
BD, Chr03	rs7653441	173374426	1	1	1	0	0.1561931011	0.1783291204
	rs3762678	133781248	2	4	3	0	0.0220474069	0.0303686009
	rs7628245	53247984	3	7	7	0	0.0112056913	0.0119339000
	rs3845903	66600360	4	3	5	0	0.0237618665	0.0150942910
	rs17046143	6848446	5	8	6	0.001	0.0099882401	0.0128369544
	rs17064749	61267686	6	39	25	0.008	0.0012765635	0.0032611066
	rs2202154	26393098	7	6	4	0.022	0.0114248320	0.0173279873
	SNP A-1957995	171063896	11	16	23	0.054	0.0044285995	0.0033596859
	rs2886966	62908574	13	41	22	0.058	0.0012622906	0.0034646266
	rs2019695	42006698	17	24	47	0.181	0.0023423896	0.0016284082
	rs711715	22985394	22	72	88	0.57	0.0007964621	0.0008602317
	rs711716	22985426	23	96	48	0.621	0.0005592881	0.0015439494
	rs33457	42378384	24	51	68	0.638	0.0009790758	0.0011299990
	rs16827563	158581672	25	56	33	0.65	0.0009621968	0.0026326921
	rs295470	140696593	26	87	27	0.692	0.0006224029	0.0031705375
	rs1872108	130722662	27	19	58	0.722	0.0034621434	0.0013304489
	SNP A-2224409	123890289	31	89	79	0.848	0.0006170623	0.0009709353
	rs1485171	7626185	35	33	41	0.888	0.0015581042	0.0019687488
	rs17015351	77696778	36	30	57	0.915	0.0016450010	0.0013339900
	rs11920711	85585246	36	76	74	0.915	0.0007795188	0.0010162714
	rs10212203	80686487	39	31	52	0.921	0.0016212017	0.0014675830
	rs17786145	1480223	80	14	9	1	0.0048801424	0.0090698610
BD, Chr21	rs16997735	39792304	1	1	3	0	0.0376263891	0.0407872915
	rs2837588	40669929	2	4	57	0.002	0.0130988202	0.0015214547
	rs7276641	25799569	3	5	10	0.019	0.0121321338	0.0106955958
	rs13340018	31477667	4	11	21	0.201	0.0080232522	0.0048628810
	rs980184	39978208	8	58	38	0.499	0.0012963971	0.0021083418
	rs999789	39348321	12	3	4	0.616	0.0239925579	0.0292488819
	rs9980699	34421519	16	38	40	0.725	0.0020291073	0.0020450120
	rs724070	33353710	40	21	65	1	0.0043161571	0.0013548513
	rs455304	40886245	46	30	15	1	0.0026384978	0.0062447878
	rs2834630	35048509	47	16	85	1	0.0049274373	0.0011372774
	rs2823980	16984585	93	95	88	1	0.0008542645	0.0011260758

Table 1 continued. Characteristics of the SNPs jointly identified as top 100s by the Single-SNP, T-Trees and hybrid FLTM / T-Trees approaches. S : Single-SNP GWAS. T : T-Trees approach. H : hybrid approach.

Pathology, chromosome	SNP identifier	Location	Rank			Corrected p-value (p) or variable importance (vi)		
			S	T	H	Single-SNP p	T-Trees vi	Hybrid vi
CAD, Chr05	rs2416472	117033845	1	4	4	0	0.0497860206	0.0589159163
	rs17076079	172898758	1	1	1	0	0.2571704213	0.2619770457
	rs159171	21222083	1	3	2	0	0.1439776507	0.1465241779
	rs17136296	113485659	4	6	6	0	0.0209207111	0.0200712296
	rs6451780	44896214	5	85	18	0.003	0.0002783858	0.0022685687
	rs17068248	166645337	6	56	8	0.033	0.0004042143	0.0049100173
	SNP A-1982751	103802011	7	18	10	0.065	0.0032242807	0.0045502730
	rs16884665	10465682	18	72	33	0.748	0.0003168655	0.0010517332
	rs325857	41080373	23	82	44	0.92	0.0002885458	0.0008485956
	rs325870	41087696	24	77	37	0.926	0.0003034803	0.0009675325
	rs11135206	161776793	28	35	76	0.968	0.0010715547	0.0004457188
CAD, Chr06	rs9478945	150205883	1	1	2	0	0.0396467499	0.0426715157
	rs519758	13522579	2	5	4	0	0.0110432778	0.0134622735
	rs17055974	96603273	3	4	7	0	0.0160990372	0.0116332061
	rs16870039	18188344	4	3	19	0.019	0.0245871598	0.0041331297
	rs1738358	49361481	5	14	3	0.037	0.0053064470	0.0140398685
	SNP A-1987732	133950890	8	18	8	0.221	0.0037822355	0.0103470304
	rs7775358	91726062	9	7	6	0.273	0.0090638963	0.0118215472
	rs9372190	108996515	10	44	23	0.322	0.0014008532	0.0036907444
	SNP A-1910247	138615031	13	65	79	0.67	0.0009570504	0.0011065328
	rs10901001	7044907	14	17	59	0.729	0.0040971056	0.0014511133
	rs1488304	94649676	17	33	50	0.771	0.0020878102	0.0018044206
	rs1474787	151295075	20	68	49	0.944	0.0009195968	0.0018602245
	rs6924328	127076726	21	37	46	0.957	0.0017783165	0.0019619287
	rs4398751	156779713	22	27	37	0.974	0.0023841409	0.0023680818
	rs1441891	165576222	24	94	57	0.987	0.0007055782	0.0015049742
	rs217540	108595042	33	47	97	1	0.0013161791	0.0008545309
	rs12529488	106791146	40	99	73	1	0.0006855112	0.0011745208
	rs6924456	49624032	49	29	83	1	0.0022640553	0.0010637405

Table 1 continued. Characteristics of the SNPs jointly identified as top 100s by the Single-SNP, T-Trees and hybrid FLTM / T-Trees approaches. S : Single-SNP GWAS. T : T-Trees approach. H : hybrid approach.

Pathology, chromosome	SNP identifier	Location	Rank			Corrected p-value (p) or variable importance (vi)		
			S	T	H	Single-SNP p	T-Trees vi	Hybrid vi
CD, Chr01	rs12078461	117273572	1	2	2	0	0.1491234057	0.1457913381
	rs1933641	216535584	1	1	1	0	0.2227965124	0.2276438256
	rs11209026	67478546	1	5	5	0	0.0303647416	0.0300032480
	rs10489629	67460937	5	22	19	0	0.0022728981	0.0052229238
	rs2201841	67466790	6	20	40	0	0.0025781386	0.0013295015
	rs12119179	67520003	7	35	65	0	0.0011869244	0.0004862244
	rs17096872	58745260	9	8	7	0	0.0155397910	0.0168155793
	rs17130103	87923130	10	6	6	0	0.0212685584	0.0223331608
	SNP A-2045577	67462196	12	48	35	0	0.0007778134	0.0016893976
	rs17101358	78377669	19	15	14	0.002	0.0047450549	0.0071728527
	rs6677092	238239905	23	11	11	0.017	0.0115811245	0.0097547179
	rs16850854	201223211	24	60	22	0.038	0.0005997367	0.0036465419
	SNP A-2111030	65186205	25	24	25	0.057	0.0021274066	0.0034337091
	rs2492958	28187010	26	83	34	0.179	0.0004693104	0.0017411612
	rs686190	12146426	31	29	29	0.29	0.0017360547	0.0020468331
	rs16847490	225488622	34	28	36	0.517	0.0018723262	0.0015801174
	rs17116805	58303794	37	70	52	0.626	0.0005289743	0.0006797639
	rs12409315	3219118	38	34	63	0.65	0.0011978668	0.0004986035
	rs13375635	219294692	56	67	47	0.972	0.0005583512	0.0008874373
CD, Chr20	rs6044514	16856038	1	1	2	0	0.0296018421	0.0452794977
	rs6125996	35788083	2	3	3	0.15	0.0092730573	0.0174648770
	rs6040103	10642742	4	2	5	0.462	0.0104239022	0.0054486522
	rs2424422	21916219	10	9	20	0.854	0.0031795414	0.0020684729
	rs16996531	9820603	12	10	26	0.912	0.0031713037	0.0018229790
	rs6012081	44992741	15	5	8	0.94	0.0065346687	0.0037916117
	rs13037694	13508308	19	34	18	0.994	0.0012633897	0.0021418804
	rs2207719	7040386	21	64	71	0.996	0.0008112721	0.0010382959
	rs6031477	42315623	26	15	10	1	0.0023139428	0.0032512736
	rs142092	7041432	30	71	45	1	0.0007718263	0.0013375214
	rs6011600	61183435	31	14	34	1	0.0023566532	0.0014966467
	rs16981111	19708553	34	30	50	1	0.0014034738	0.0012974103
	rs12480690	19703487	40	29	81	1	0.0014082033	0.0009364693
	rs16996688	9976725	43	12	76	1	0.0027739249	0.0009822003
	rs6047596	2173481	66	11	40	1	0.0029700860	0.0013643799
	rs6086197	7705151	84	25	24	1	0.0016527316	0.0019267975
	rs6127097	52157302	91	19	37	1	0.0019335780	0.0014499697
	rs844912	23088535	100	59	84	1	0.0008496331	0.0008966419

Table 1 continued. Characteristics of the SNPs jointly identified as top 100s by the Single-SNP, T-Trees and hybrid FLTM / T-Trees approaches. S : Single-SNP GWAS. T : T-Trees approach. H : hybrid approach.

**Pathology, chromosome**	**SNP identifier**	**Location**	**Rank**			**Corrected p-value (p) or variable importance (vi)**		
			**S**	**T**	**H**	**Single-SNP p**	**T-Trees vi**	**Hybrid vi**
HT, Chr10	rs11005510	58202995	1	1	2	0	0.0841503659	0.0865388191
	rs17157250	1913202	2	12	19	0.15	0.0047277850	0.0043408900
	rs11185734	91253844	3	47	63	0.231	0.0013029791	0.0013969302
	rs7905911	107754628	4	15	22	0.319	0.0032399618	0.0036558188
	rs1691907	29165925	27	29	20	0.943	0.0019557906	0.0041493699
	rs7081531	21355069	29	28	42	0.954	0.0020499962	0.0018588073
	rs7894018	92807259	33	43	7	0.984	0.0014401931	0.0089155660
	rs2458705	2531377	41	23	49	0.996	0.0022880667	0.0017362040
	rs6415963	21280349	48	93	88	0.999	0.0006914531	0.0009673225
	rs10881889	92878888	96	21	21	1	0.0024471637	0.0036700870
HT, Chr14	rs8011855	38673786	1	5	5	0	0.0298551353	0.0406287873
	rs1957779	62739400	2	7	13	0	0.0284964121	0.0114005153
	rs1457015	82704632	3	18	10	0.002	0.0092820857	0.0129045232
	rs6574988	87629745	4	9	8	0.004	0.0175690479	0.0234725902
	rs7142143	50473281	5	38	17	0.004	0.0028333437	0.0097743195
	rs2653550	78672810	6	29	14	0.026	0.0038620063	0.0113375370
	rs1253672	51459138	7	74	46	0.03	0.0007985511	0.0018276704
	SNP A-2203090	32214885	8	30	49	0.217	0.0037660919	0.0016873485
	rs6574590	80151227	9	61	44	0.443	0.0012010403	0.0022137415
	rs17098915	61129684	11	41	36	0.486	0.0024481089	0.0038094737
	rs10483456	35105918	13	97	29	0.628	0.0006267321	0.0050535673
	rs1958305	23342964	21	8	12	0.889	0.0187541270	0.0122218227
	rs1241649	24383933	22	68	40	0.916	0.0009770084	0.0027470373
	rs435340	44004257	30	39	25	0.982	0.0027815511	0.0062562922
	rs7149343	67634464	35	40	32	0.998	0.0027254172	0.0048198426
	rs7150537	92331535	52	54	38	1	0.0014284773	0.0030667692
	rs2194622	44358898	62	63	97	1	0.0011662734	0.0007270501
	rs2400997	100796860	66	62	61	1	0.0011882915	0.0013264366

Table 1 continued. Characteristics of the SNPs jointly identified as top 100s by the Single-SNP, T-Trees and hybrid FLTM / T-Trees approaches. S : Single-SNP GWAS. T : T-Trees approach. H : hybrid approach.

**Pathology, chromosome**	**SNP identifier**	**Location**	**Rank**			**Corrected p-value (p) or variable importance (vi)**		
			**S**	**T**	**H**	**Single-SNP p**	**T-Trees vi**	**Hybrid vi**
RA, Chr06	rs10499044	107247988	1	4	4	0	0.0630765559	0.0697904323
	rs2677821	133963704	1	7	3	0	0.0520663246	0.0726410123
	rs2282859	167365129	1	9	10	0	0.0163503373	0.0286484387
	rs9271850	32703038	1	1	1	0	0.1585047003	0.1709058105
	rs3129768	32703061	1	3	5	0	0.0841176743	0.0553963826
	rs9272346	32712350	6	13	14	0	0.0067641638	0.0097011948
	rs9272723	32717405	7	22	28	0.003	0.0024888724	0.0041009373
	rs2876370	137958930	8	45	18	0.006	0.0006254908	0.0064546828
	rs943081	43988645	11	89	34	0.176	0.0002823740	0.0018685358
	rs2523691	31528666	19	12	81	0.735	0.0088735015	0.0005068029
	rs12174860	64974391	30	39	22	0.999	0.0007600187	0.0048832518
RA, Chr19	rs11671119	19147077	1	1	1	0	0.3720436110	0.3901345027
	rs8112647	37846151	2	2	3	0	0.0275005327	0.0257925134
	rs7260239	51127649	3	5	10	0.071	0.0055668845	0.0045807162
	rs16968393	38869515	4	4	22	0.096	0.0060452119	0.0029202441
	rs10403038	24032496	5	35	95	0.187	0.0011845548	0.0005818320
	rs4646530	15596238	7	18	7	0.454	0.0018113494	0.0092447061
	rs443239	47197884	11	15	45	0.847	0.0021266099	0.0013825310
	rs1045354	9134300	12	56	60	0.874	0.0009004796	0.0010616469
	rs1076404	37384171	20	9	86	0.994	0.0046168709	0.0006455583
	rs7254234	60701027	21	41	89	0.996	0.0010862642	0.0006222316
	rs184583	35063430	23	50	85	0.996	0.0009567363	0.0006490994
	rs10418932	45492128	31	53	56	1	0.0009272069	0.0011442688
	rs8112226	6098939	34	46	33	1	0.0010408028	0.0021449315
	rs279223	11852638	50	75	74	1	0.0006980925	0.0007193191
	rs1673894	57667055	53	24	24	1	0.0015184478	0.0027686316

Table 1 continued. Characteristics of the SNPs jointly identified as top 100s by the Single-SNP, T-Trees and hybrid FLTM / T-Trees approaches. S : Single-SNP GWAS. T : T-Trees approach. H : hybrid approach.

Pathology, chromosome	SNP identifier	Location	Rank			Corrected p-value (p) or variable importance (vi)		
			S	T	H	Single-SNP p	T-Trees vi	Hybrid vi
T1D, Chr02	rs17007623	61334969	1	5	2	0.001	0.0133742298	0.0195961864
	rs934776	85858744	2	11	4	0.005	0.0071723776	0.0172738101
	rs12476453	4118090	3	60	15	0.014	0.0006280073	0.0027831733
	rs903228	53545553	4	7	64	0.19	0.0110726358	0.0011338601
	rs17595217	205261744	7	96	21	0.633	0.0004820676	0.0023279291
	rs16857192	133613479	10	20	20	0.738	0.0019886588	0.0023510407
	SNP A-2312906	145636667	15	15	36	0.869	0.0031340497	0.0015337774
	rs6750794	117897430	21	26	51	0.967	0.0014973507	0.0012443585
	rs2681035	23087764	25	17	38	0.979	0.0029656365	0.0015269817
	rs4848687	121639597	28	18	83	0.988	0.0024862257	0.0008971243
	rs951840	49814673	46	43	91	1	0.0007557003	0.0008458140
	rs4266033	194870695	47	40	59	1	0.0008232962	0.0011828586
	rs6546086	64625650	62	67	77	1	0.0005941744	0.0009348953
	rs4553871	134182116	82	68	79	1	0.0005913888	0.0009172388
	rs2218549	46291323	91	13	9	1	0.0042034781	0.0065853168
T1D, Chr13	rs4254200	82213653	1	2	1	0	0.2538874379	0.3727425083
	rs16946406	91101609	2	15	8	0.069	0.0028103972	0.0061699275
	rs7317472	36700636	4	16	17	0.13	0.0025256870	0.0029329278
	rs3002099	18999964	5	57	22	0.13	0.0003716672	0.0020762461
	rs9514597	106408722	6	18	19	0.336	0.0020581485	0.0027848160
	rs12708382	101444814	7	25	20	0.344	0.0008447923	0.0027549027
	rs391286	92654382	10	95	55	0.673	0.0002249988	0.0005268828
	rs9315704	39038215	12	53	30	0.927	0.0004241478	0.0011241682
	rs2325562	73245093	20	33	45	0.999	0.0006724809	0.0007147068
	rs1764773	104213047	24	41	50	1	0.0005124660	0.0005994729
	rs288700	106336283	30	19	65	1	0.0014049832	0.0004403043
	rs17068485	36799218	85	23	81	1	0.0010976141	0.0003465394

Table 1 continued. Characteristics of the SNPs jointly identified as top 100s by the Single-SNP, T-Trees and hybrid FLTM / T-Trees approaches. S : Single-SNP GWAS. T : T-Trees approach. H : hybrid approach.

Pathology, chromosome	SNP identifier	Location	Rank			Corrected p-value (p) or variable importance (vi)		
			S	T	H	Single-SNP p	T-Trees vi	Hybrid vi
T2D, Chr10	rs4506565	114746031	1	27	22	0	0.0024009775	0.0043359677
	rs7901695	114744078	2	17	26	0	0.0034539411	0.0035669469
	rs7077039	114779067	3	13	18	0	0.0047124557	0.0055736223
	rs4132670	114757761	4	38	32	0	0.0014877667	0.0029378345
	rs10787472	114771287	5	75	66	0	0.0007878316	0.0013403116
	rs11196205	114797037	6	84	89	0	0.0007051230	0.0010264811
	rs4074720	114738487	9	53	63	0	0.0009835015	0.0014216117
	rs12243326	114778805	10	60	75	0	0.0008930770	0.0012491396
	rs2479037	114586715	11	1	5	0.001	0.0186489590	0.0160654034
	rs17094393	117981552	12	4	7	0.001	0.0124231173	0.0127614245
	rs11006039	59495478	14	3	11	0.004	0.0124704060	0.0068231524
	rs293284	52888865	16	7	6	0.14	0.0079004995	0.0130521405
	rs7898565	99965717	17	11	24	0.256	0.0049178430	0.0036458166
	rs7082404	77116071	21	5	3	0.371	0.0115409548	0.0224677955
	rs11190376	101788618	24	46	74	0.43	0.0011934485	0.0012635745
	rs7894018	92807259	27	12	10	0.678	0.0048441347	0.0110033719
	rs7079515	75702417	31	39	81	0.752	0.0014343174	0.0011615070
	rs2813383	1611800	41	20	35	0.989	0.0031712388	0.0026390874
	rs1961317	120644012	47	25	34	0.999	0.0025425871	0.0026937406
	rs505699	99764066	53	88	79	0.999	0.0006645506	0.0012017055
	rs12265675	84069725	67	68	71	1	0.0008582652	0.0013029158
	rs1566222	70189609	76	14	19	1	0.0042274703	0.0055602542
	rs2250544	84437807	78	37	50	1	0.0014933012	0.0017425263
T2D, Chr21	rs226261	26953847	1	1	1	0	0.2469128707	0.2108330173
	rs7276641	25799569	3	52	53	0.578	0.0007131539	0.0009841657
	rs8130806	21330548	14	42	38	1	0.0008236536	0.0014054434
	rs435260	35816526	20	97	99	1	0.0004865401	0.0005820725
	rs16994356	36927512	25	48	51	1	0.0007691225	0.0010050038
	rs220161	42422362	38	50	77	1	0.0007314639	0.0007198498
	rs17000913	41730743	83	19	78	1	0.0018215928	0.0007088186
	rs764967	35221535	93	20	25	1	0.0017779083	0.0022457495
